# Fasting, but Not Aging, Dramatically Alters the Redox Status of Cysteine Residues on Proteins in *Drosophila melanogaster*

**DOI:** 10.1016/j.celrep.2015.05.033

**Published:** 2015-06-18

**Authors:** Katja E. Menger, Andrew M. James, Helena M. Cochemé, Michael E. Harbour, Edward T. Chouchani, Shujing Ding, Ian M. Fearnley, Linda Partridge, Michael P. Murphy

**Affiliations:** 1 MRC Mitochondrial Biology Unit, Cambridge CB2 0XY, UK; 2 Institute of Ophthalmology, University College London, London EC1V 9EL, UK; 3 Institute of Healthy Ageing and GEE, University College London, London WC1E 6BT, UK; 4 Max Planck Institute for Biology of Ageing, Cologne 50931, Germany; 5 MRC Clinical Sciences Centre, Imperial College London, London W12 0NN, UK; 6 Department of Medicine, University of Cambridge, Cambridge CB2 0QQ, UK; 7 Department of Cancer Biology, Dana-Farber Cancer Institute, Boston, MA 02215, USA; 8 Department of Cell Biology, Harvard Medical School, Boston, MA 02115-5730, USA

## Abstract

Altering the redox state of cysteine residues on protein surfaces is an important response to environmental challenges. Although aging and fasting alter many redox processes, the role of cysteine residues is uncertain. To address this, we used a redox proteomic technique, oxidative isotope-coded affinity tags (OxICAT), to assess cysteine-residue redox changes in *Drosophila melanogaster* during aging and fasting. This approach enabled us to simultaneously identify and quantify the redox state of several hundred cysteine residues in vivo. Cysteine residues within young flies had a bimodal distribution with peaks at ∼10% and ∼85% reversibly oxidized. Surprisingly, these cysteine residues did not become more oxidized with age. In contrast, 24 hr of fasting dramatically oxidized cysteine residues that were reduced under fed conditions while also reducing cysteine residues that were initially oxidized. We conclude that fasting, but not aging, dramatically alters cysteine-residue redox status in *D. melanogaster*.

## Highlights

• The redox state and identity of cysteine residues in flies can be determined by OxICAT
• Overall cysteine-residue redox state does not change with age
• H_2_O_2_ and paraquat have surprisingly distinct effects on cysteine-residue redox state
• Fasting for 24 hr dramatically alters the redox state of cysteine residues

## Graphical Abstract

**Figure:**
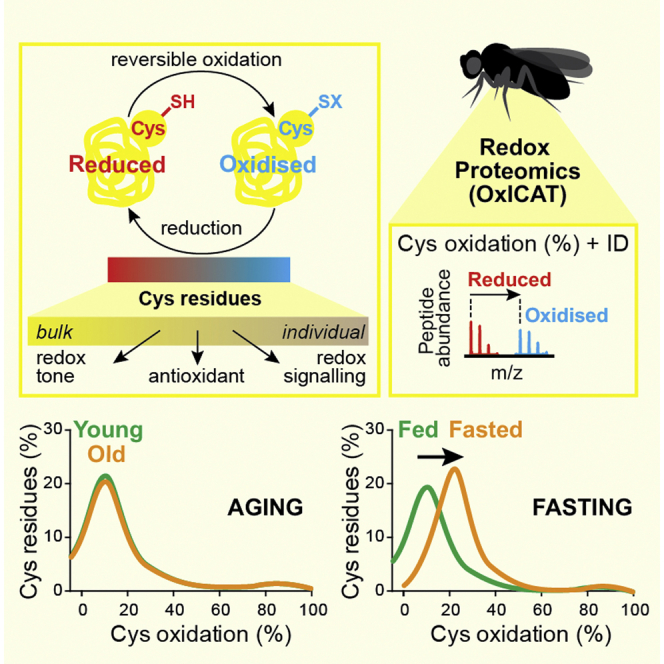

## Teaser

Using a redox proteomic mass spectrometry technique, oxidative isotope-coded affinity tags (OxICAT), Menger et al measured cysteine residue redox changes in *Drosophila melanogaster* during aging and fasting. Surprisingly, aging had no impact on cysteine-residue redox state. In contrast, fasting dramatically affected cysteine residues, suggesting a role for cysteine-residue redox status in fasting.

## Introduction

Organisms are continually exposed to environmental challenges that dramatically alter redox processes, changing the reduction potential of redox couples as well as the production of evanescent reactive species (Go and Jones, 2013; Murphy, 2012). These redox changes can disrupt the molecular machinery of the organism, and consequently, cells contain short-term adaptive mechanisms and a parallel capacity for activating gene expression to maintain resilience. Two important environmental challenges that involve redox changes are aging and fasting.

Aging correlates with changes in redox couples, increases in reactive species, and oxidative damage (Go and Jones, 2013; Cochemé et al., 2011), although their relationship with the mechanisms underlying aging has proven elusive. Fasting for 12–48 hr dramatically alters metabolic processes and is protective against ischemia-reperfusion injury (Robertson and Mitchell, 2013) and alters signaling pathways in flies (Le Bourg, 2013; Webster et al., 2014). Intermittent starvation can be particularly effective in improving health and extending lifespan, and it may mediate some of the effects of dietary restriction (DR) (Fontana and Partridge, 2015). In addition, it is not clear if DR slows changes that occur during aging or instead protects against their consequences for health and mortality (Fontana and Partridge, 2015). While the molecular mechanisms underlying the benefits of fasting are obscure (Robertson and Mitchell, 2013), redox alterations are likely to be central.

**Figure 1:**
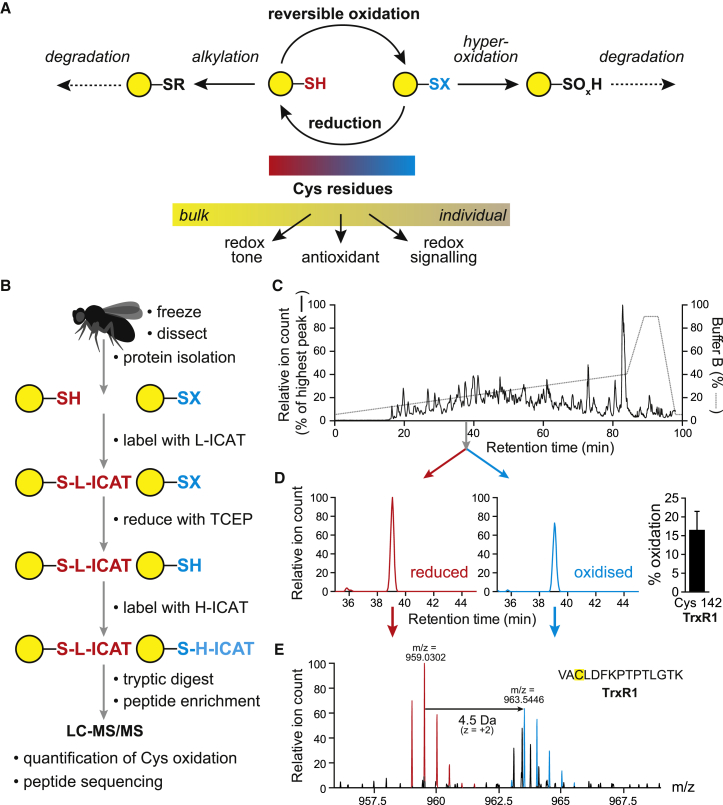
Assessment of Protein Cysteine-Residue Redox State in Flies (A) Schematic showing how exposed cysteine residue can be reversibly oxidized and reduced by GSH/glutaredoxin (Grx) and Trx. (B) OxICAT methodology. Flies are rapidly frozen, and the heads and thoraces are homogenized in 100% TCA to separate solubilized protein from the exoskeleton and then diluted to 20% TCA to precipitate proteins. The protein homogenate is then reacted with the Light ICAT reagent (L-ICAT, red) to label reduced cysteine residues (Pr-SH). After reduction of reversibly oxidized cysteine residues (Pr-SX), these thiols are reacted with the heavy ICAT reagent (H-ICAT, blue). After tryptic digestion and enrichment of labeled peptides, the biotin tags are cleaved off before separation by liquid chromatography and analysis by mass spectrometry, enabling the peptide sequence and the ratio of heavy and light labeled cysteine-containing peptides to be determined simultaneously. (C) A typical chromatogram from control flies (UAS-cat/+). A cysteine peptide oxidized and reduced pair (retention time = 39 min) is highlighted. (D) Chromatograms for the heavy and light labeled peptide eluting at 39 min are shown. The percentage oxidation of that cysteine residue was determined (bar chart). (E) The peptide eluting at 39 min was identified by mass spectrometry as a component of thioredoxin reductase-1 (TrxR1). This gene encodes both a mitochondrial and a shorter cytoplasmic splice variant. The peptide could arise from either isoform but has been numbered as Cys142 from the mitochondrial isoform. See also Figure S1.

To explore how aging and fasting affect redox state, we used the fruit fly *Drosophila melanogaster* and focused on reversible redox alterations to exposed cysteine residues. These often lack a clear structural or catalytic role and are a major, but underappreciated, component of the integrated response of the cell to redox alterations (Go and Jones, 2013; Murphy, 2012). Cysteine residues are the most abundant cellular thiol, and in the mitochondrial matrix, the concentration is ∼20- to 30-fold greater than glutathione (GSH) (Go and Jones, 2013; Requejo et al., 2010). A proportion of protein thiols are particularly reactive due to changes in pK_a_, accessibility and orientation wrought by the local environment (Go and Jones, 2013; Held and Gibson, 2012). Potential modifications to cysteine residues include disulfides, S-nitrosothiols, sulfenic acids, S-acylation, and S-thiolation, all of which can be reversed by the GSH/glutaredoxin and thioredoxin (Trx) systems (Murphy, 2012; Go and Jones, 2013; Held and Gibson, 2012). These changes are part of the bulk redox tone, and small changes to a large number of different cysteine residues are likely to buffer the cellular redox environment to cope with changes in redox couples and reactive species (Go and Jones, 2013). Protein cysteine residues can also prevent local damage by sequestering reactive species (Go and Jones, 2013). Finally, a proportion of protein cysteine residues will undergo reversible modifications that can alter protein activity, location, or function and thereby coordinate the transmission of redox signals (D’Autréaux and Toledano, 2007; Sobotta et al., 2015). Therefore, cysteine residues are central to the cellular response to environmental challenges through the bulk redox tone or by more specific contributions to antioxidant defenses and redox signaling (Go and Jones, 2013; Held and Gibson, 2012; Leichert et al., 2008; Murphy, 2012). Consequently, assessing shifts in redox state as well as the identities of individual cysteine residues that change will contribute to our understanding of how organisms respond to aging and fasting (Figure 1A).

Assessing protein cysteine-residue redox changes is technically demanding, due to the range and evanescent nature of reversible redox changes and to the large number of residues involved, and because specific modifications to particular residues as well as small shifts in the population are both important. To address this, the ICAT (isotope-coded affinity tags) method was adapted for redox proteomics as the oxidative isotope-coded affinity tags (OxICAT) approach (Leichert et al., 2008) (Figure 1B). This enables the redox state of a large number of cysteine residues to be determined simultaneously, as well as the identification of the individual cysteines involved. The OxICAT approach has been used to investigate reversible cysteine residue oxidation within *Escherichia coli* (Leichert et al., 2008), *Saccharomyces cerevisiae* (Brandes et al., 2011), *Caenorhabditis elegans* (Knoefler et al., 2012), rat sperm (Baker et al., 2015), and mammalian cells (Go et al., 2011). Here, we have extended the OxICAT approach to *D. melanogaster* (Figure 1B) to assess reversible redox changes to cysteine residues during aging and fasting.

## Results

### Using OxICAT to Measure Cysteine Residue Redox State in Flies

We used cohorts of ten control female flies to reduce biological variation and rapidly froze these before separating the heads and thoraces from the abdomens (Figure 1B). We focused on non-reproductive tissues to isolate the effects of age and fasting on similar tissue types, as the female abdomen changes markedly with age. Cysteine residues were stabilized by homogenization in trichloroacetic acid (TCA) to prevent artifactual thiol oxidation and disulfide shuffling (Held and Gibson, 2012; Leichert et al., 2008), then proteins were precipitated and processed for OxICAT analysis (Figure 1B). Example chromatograms and mass spectra are shown in Figures 1C–1E. Overall, peaks were observed for ∼1,191 cysteine residues on ∼1,082 peptides, which were labeled by both the heavy and light ICAT reagents, corresponding to ∼424 proteins (Tables S1 and S2).

**Figure 2:**
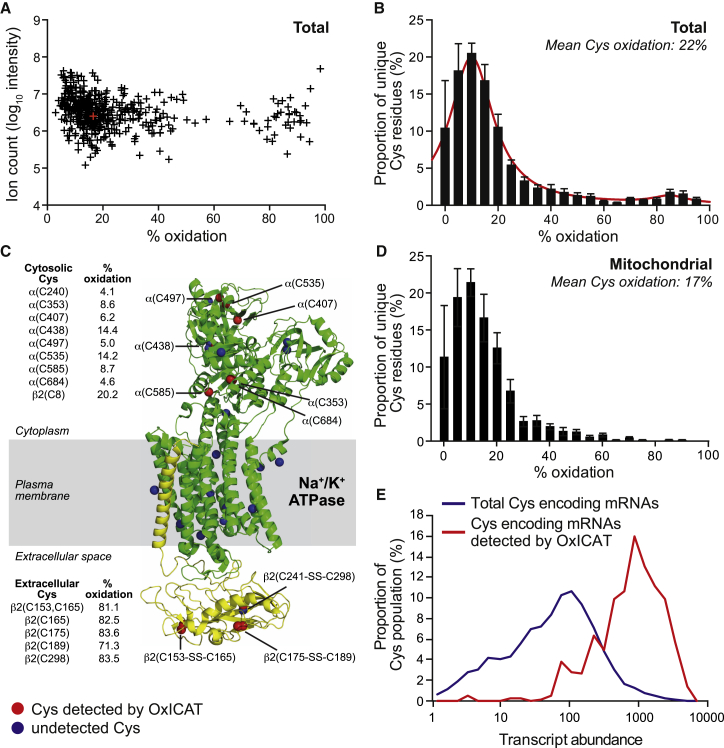
OxICAT Analysis of Control Young Female *D. melanogaster* (A) Ion count for peptides plotted against percentage oxidation of the cysteine residue. The ion count is the log_10_ intensity of the sum of the heavy and light peptides. Data are the averages over three to five biological replicates. Red cross is cysteine residue 142 from TrxR1 (Figure 1C). (B) Distribution of total cysteine residue oxidation levels. Plotted are the means of the proportion of the total number of peptides containing unique cysteine residues in each 5% quantile of percentage oxidation across five biological replicates (mean ± SEM). Total unique peptides = 491. (C) Plasma membrane Na^+^/K^+^ ATPase. *D. melanogaster* Na^+^/K^+^ ATPase contains an α subunit and a β subunit with multiple isoforms. The monomeric structure from *S. acanthias* containing subunit α (green) and subunit β1 (yellow) is 77% and 25% homologous to the α and β2 subunits of *D. melanogaster*, which were detected by OxICAT. Cysteine residues on the *S. acanthias* structure present in homologous positions in the *D. melanogaster* α and β2 subunits are numbered. Cysteines observed by OxICAT are shown in red, and those not detected are blue. Disulfide cysteine partners are also labeled. The table shows the oxidation state of each cysteine in young control untreated flies. (D) Oxidation state of protein cysteine residues in mitochondria. Peptides from Figure 2B that are mitochondrial are plotted as the mean of the proportion of the total number of peptides in each 5% quantile of percentage oxidation across five biological replicates (mean ± SEM). Of 214 proteins identified in Figure 2A, 87 are mitochondrial, corresponding to 214 unique cysteine residues. (E) Comparison of peptides detected by OxICAT with transcript abundance. Whole-fly transcript intensity data were annotated to the head and thorax OxICAT dataset to characterize fly cysteines observable by mass spectrometry. Transcripts are subdivided by abundance into blocks that are √2 of the upper and lower bounds of the block immediately to the left. The percentage of both the observed OxICAT cysteine population (mean transcript abundance = 1,119; n = 849) and the total cysteine population (mean transcript abundance = 134; n = ∼135,000) falling within each transcript abundance block are on the y axis. See also Figure S2.

In Figure 2A, the log_10_ intensity of the ion count for the peptide is plotted against the percentage oxidation of that cysteine residue. This shows there is no correlation between abundance and oxidation state that could indicate systematic bias in the methodology. We only considered cysteine residues that were labeled with both light and heavy ICAT labels in at least three biological replicates out of five. In control flies, we quantified the percentage oxidation of ∼537 cysteine residues, on 491 peptides, corresponding to 214 proteins (Figure 2A; Table S1). This compares favorably with previous OxICAT analyses, which based conclusions on ∼400 peptides from 290 proteins in yeast (Brandes et al., 2011), 170 peptides from 137 proteins in *C. elegans* (Knoefler et al., 2012), and 641 peptides from 333 proteins in mammalian cells (Go et al., 2011).

When the cysteine population of each replicate was grouped into 5% quantiles (Figure 2B), the level of reversible oxidation was clustered around a mode of ∼10%, with a small number ∼85% redox modified (Figure 2B). This was in agreement with other OxICAT studies, which found that the majority of cysteine residues are partially (∼5%–25%) oxidized (Brandes et al., 2011; Go et al., 2011; Knoefler et al., 2012; Leichert et al., 2008).

Cysteine residues with low signal intensity were frequently measured as 0% and 100% oxidized because only the light or the heavy labeled peptide was detected. We have discarded these data points to minimize distortions due to peptide abundance. However, this may exclude cysteine residues that are fully reduced or oxidized in vivo. When we reassessed the data in Figure 2A to include peptides labeled by only one ICAT reagent, we expanded the number of peptides to 862 (Figure S2A). However, we only detected 6 fully reduced (red symbols; Figure S2A) and 14 fully oxidized cysteine residues (blue symbols; Figure S2A). Thus, most cysteine residues are partially oxidized, and excluding singly labeled cysteine-containing peptides does not distort our analysis.

To assess the reliability of OxICAT labeling, we treated tissue homogenates with the reducing agent tris(2-carboxyethyl)phosphine (TCEP) prior to analysis and this lowered the oxidation state of the cysteine residues (Figure S2B). Similarly, oxidation of homogenates with H_2_O_2_ greatly increased cysteine residue oxidation (Figure S2C). Therefore our analyses accurately reflect protein thiol redox states in fly homogenates.

The weighted arithmetic mean of the % oxidation of cysteine residues in Figure 2B is ∼22%. To assess the average cysteine residue redox state by an orthogonal technique, we quantified protein thiols with DTNB, giving 144 ± 25 nmol thiol/mg protein (n = 3 ± SEM), then treated the sample with DTT, which increased the thiols detected to 195 ± 12 nmol thiol/mg protein (n = 3 ± SEM), implying a percentage protein thiol oxidation of ∼26%, similar to that obtained from OxICAT (Figure 2B).

As expected, some of the highly oxidized cysteine residues identified in control flies were on extracellular proteins such as transferrin 1 and general odorant binding protein 99a (Table S1). In contrast, intracellular proteins such as heat shock protein 83 and GAPDH II had more reduced cysteine residues. To further illustrate compartmentalization of redox state, we considered the Na^+^/K^+^-ATPase, which has intracellular and extracellular cysteine residues (Shinoda et al., 2009) (Figure 2C). The β-subunit of the Na^+^/K^+^-ATPase (Q24048) has six cysteine residues in the relatively oxidized extracellular space that form three disulfides in the molecular structure (2ZXE). Five of the extracellular cysteine residues from the β_2_ isoform that we observed by OxICAT were oxidized (Figure 2C; 80.4% ± 2.3%). This β_2_ isoform also contains a cytoplasmic cysteine residue, and this was largely reduced (20.2%). An additional eight cysteine residues on the cytoplasmic domain of the α-subunit (E1JIR4) were largely reduced (8.2% ± 1.5%). Therefore, OxICAT reports oxidation states consistent with the known redox state and location of cysteine residues and can detect differences within a protein.

Next, we assessed cysteine residues from Figure 2B that were from the 87 proteins annotated as mitochondrial (Figure 2D). The distribution for mitochondria was similar to that for the whole fly with a mode of ∼10% oxidized but with far fewer highly oxidized cysteine residues, presumably due to exclusion of extracellular and ER proteins (Figure 2B).

Our next goal was to determine what proportion of cysteine residues in transcribed proteins were represented in the OxICAT datasets. Based on an in silico digest, the total number of unique cysteines in the whole fly genome is ∼135,000 on ∼98,000 unique peptides. We clearly observe only a small fraction of the total; 849 tryptic peptides that contain 966 cysteine residues were observed at least once in control flies, and of these, 491 peptides were observed at least three times. Thus, we observe ∼0.72% of cysteine residues in the fly genome, although many of these are not expressed in the head and thorax of the adult female fly. Furthermore, the OxICAT method will predominantly reflect the redox state of abundant proteins. To assess this, we used literature levels of mRNA transcript intensity within whole adult flies (Chintapalli et al., 2007) to assign each cysteine residue a relative abundance (Figure 2E, blue line). We then compared this with the transcript abundance of the mRNA subset encoding those peptides detected by OxICAT (Figure 2E, red line). The mean abundance for transcripts encoding cysteine residues detected by OxICAT is ∼8.4-fold higher than that of the whole genome; thus, OxICAT detects redox changes in the most abundant proteins (Figure 2E). Even though we observed only ∼0.72% of the potentially observable cysteine residues, these are the most abundant ones and thus contribute comparatively more to the cell redox state. If transcription reflects translation, then the cysteine residues that we assessed represent ∼6% of cysteine residues by concentration within an adult fly. This is comparable with yeast studies, where OxICAT detected the oxidation state of ∼5% of yeast protein thiols (Brandes et al., 2011). Even so, OxICAT should reflect the redox state of the cysteine-residue population well enough to assess biologically important questions.

### No Change in Cysteine-Residue Redox State with Age

**Figure 3:**
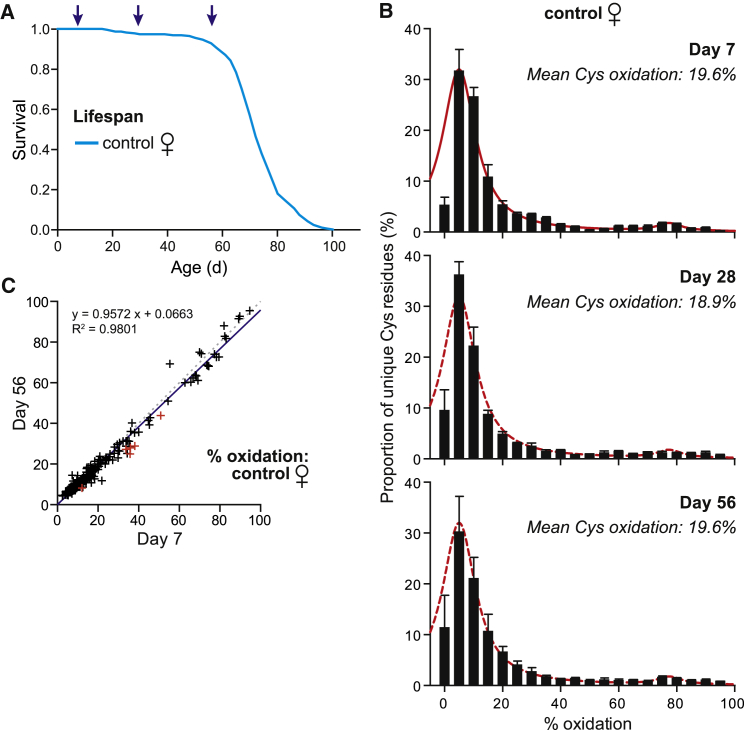
Reversible Oxidation Levels of Cysteine Residues in Aging *D. melanogaster* (A) Lifespan of control female flies. Cohorts of flies were taken to analyze protein cysteine residue redox state of young (7 days), middle-aged (28 days), and old (56 days) flies. (B) Distribution of cysteine peptides plotted against their redox states for 7-, 28-, and 56-day-old control flies. Data are means ± SEM. The red curve is for 7-day-old control flies. (C) Oxidation state of cysteine residues present in 56-day-old flies plotted against 7-day-old flies. The dotted line slope = 1, while the continuous line is the least-squares best-fit line to the data. Data from 263 unique peptides identified at least three times under both conditions are plotted. Red symbols (n = 6) indicate low-stringency significance with p < 0.05 assessed by a non-paired, two-tailed Student’s t test. See also Figure S3.

H_2_O_2_ is a key mediator of thiol redox state that increases with age in flies (Cochemé et al., 2011). Aging has also been correlated with an increase in oxidative damage in flies (Jacobson et al., 2010), and protein thiols become oxidized upon chronological aging in yeast (Magherini et al., 2009). We used OxICAT to quantify the effect of aging on the oxidation of cysteine residues (Figure 3A). Surprisingly, despite increases in H_2_O_2_ (Cochemé et al., 2011) and oxidative damage with age, the cysteine-residue oxidation state did not shift between young (7 days), middle-aged (28 days), and old (56 days) control flies, and the weighted mean percentage oxidation was also almost unaffected (Figure 3B). To see if there were shifts in the redox state of individual proteins with age that were masked by the overall trend, we plotted the redox state of individual cysteine residues detected in both the young and old control flies and again observed no change in redox state (Figure 3C). Similarly, there were no changes between 7 days and 28 days (Figure S3A) or between 28 days and 56 days (Figure S3B; Table S3).

### Effects of H_2_O_2_ and Paraquat on Cysteine-Residue Redox State

**Figure 4:**
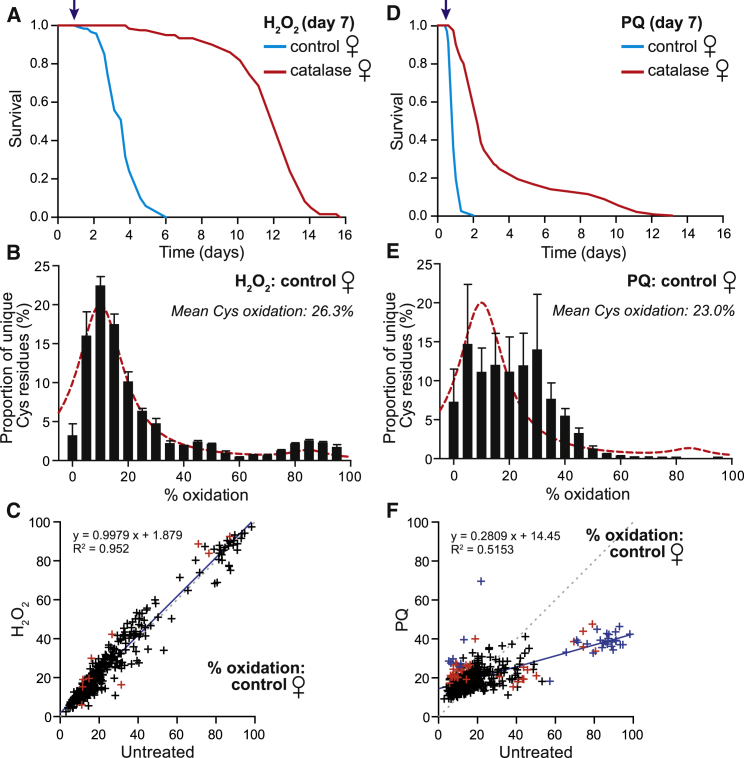
Effect of Exogenous Oxidants on Protein Cysteine-Residue Redox State and Fly Survival (A) Survival of young (7 days) control and catalase-overexpressing flies after exposure to H_2_O_2_. Arrow indicates when cohorts are collected (24-hr treatment). (B) Distribution of cysteine peptides plotted against redox states of the cysteine residues for control flies after exposure to H_2_O_2_. Data show the mean of five biological samples where each cysteine residue identified is sorted into corresponding 5% quantiles, and the resulting distributions are averaged (mean ± SEM). Dashed line indicates the untreated control (cf. Figure 2B). (C) Oxidation state of cysteine residues present in at least three biological replicates exposed to H_2_O_2_ plotted against the same cysteine residues present in at least three biological replicates of controls. Dotted line slope = 1, whereas the continuous line is the best fit to the data. Red symbols (n = 12) indicate cysteine residues significantly different following a non-paired, two-tailed Student’s t test (p < 0.05). Total unique peptides = 452. (D) Survival of young (7 days) control and catalase overexpressing flies after exposure to PQ. Arrow indicates where cohorts are sampled (24-hr treatment). (E) Distribution of cysteine-containing peptides plotted against redox states of the cysteine residues for control flies after exposure to PQ. Means are across five biological replicates of the relative number of cysteine residues within each 5% quantile. The dashed line is the untreated control cohort (cf. Figure 2B). (F) Oxidation state of cysteine residues in control flies exposed to PQ plotted against untreated flies. Dotted line slope = 1, while the continuous line is the line of best fit. Each symbol represents a cysteine residue identified in at least three biological replicates of the untreated as well as the PQ-treated cohort. Red symbols identify cysteine residues (n = 68; p < 0.05; non-paired, two-tailed Student’s t test with low-stringency significance). The blue symbols (n = 33) indicate a high-stringency significance (Benjamini-Hochberg test). Total unique peptides = 452. See also Figure S4.

Given that a lack of cysteine-residue oxidation with age was surprising, we investigated whether cysteine-residue oxidation responded to H_2_O_2_ in vivo. Dietary H_2_O_2_ dramatically decreased survival of control flies, and overexpressing catalase conferred resistance (Figure 4A). Next, we analyzed control untreated and H_2_O_2_-treated flies by OxICAT, and we found there was a marginal oxidation of the cysteine residues (26.3%; Figure 4B) in comparison to untreated control flies (22%; Figure 2B). The redox state of the individual cysteine residues following H_2_O_2_ treatment was then plotted against those in untreated control flies (Figure 4C). If H_2_O_2_ treatment did not affect cysteine-residue redox state, then the points would lie on the dotted line, and H_2_O_2_ shifted very few peptides above this line (Figure 4C). Overexpressing catalase had little effect on cysteine residue oxidation by H_2_O_2_ (Figures S4A and S4B). Thus, surprisingly, H_2_O_2_ levels that dramatically decrease survival did so without oxidizing cysteine residues (Figure 4C).

The redox cycler paraquat (PQ) also drastically decreased fly survival, and this was partially rescued by catalase (Figure 4D). However, in contrast to H_2_O_2_, PQ toxicity was associated with a dramatic oxidation of cysteine residues (Figure 4E). The weighted arithmetic mean of the cysteine-residue oxidation state was not increased by PQ due to the parallel loss of highly oxidized cysteine residues (23%; Figure 4E), reinforcing the usefulness of OxICAT relative to other bulk sampling techniques. When we plotted the effect of PQ on the redox state of individual cysteine residues, many that were <20% oxidized in the untreated control became more oxidized upon PQ treatment, moving above the dotted line (Figure 4F). In contrast, PQ exposure decreased the oxidation of those cysteine residues that were >20% oxidized in the untreated control, moving them below the dotted line (Figure 4F). These contrasting effects meant that there was only a weak correlation between untreated control and PQ exposure. In addition, the oxidation of cysteine residues by PQ was attenuated by catalase overexpression (Figures S4C–S4E). Interestingly, catalase overexpression had no effect on the PQ-induced decrease in oxidation of cysteine residues. Those cysteine residues that showed significant differences (Benjamini-Hochberg test) are identified by blue crosses in Figure 4F and given in Table S4.

### Effect of Fasting on Cysteine Residue Redox State

**Figure 5:**
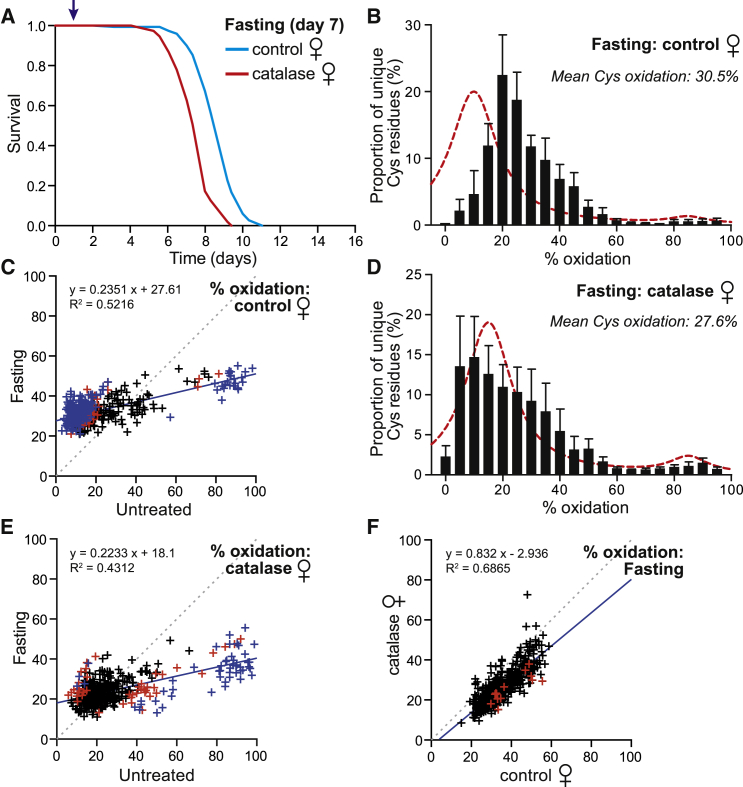
Effect of Fasting on Protein Cysteine-Residue Oxidation and Survival (A) Survival of young (7 days) control and catalase-overexpressing flies during fasting. Arrow indicates where cohorts are sampled (24-hr treatment). (B) Distribution of cysteine peptides plotted against redox states of the cysteine residues for control flies after 24-hr fasting. Shown is the mean for the relative numbers of cysteine residues in each 5% quantile of the five biological replicates. The dashed line is the curve for the untreated control (cf. Figure 2B) cohort. (C) Oxidation state of cysteine residues present in control flies upon 24-hr fasting compared to untreated cohorts. Dotted line slope = 1, while the continuous line is the best fit to the data. Each symbol represents a cysteine residue identified in at least three biological replicates of both the control untreated as well as the fasted cohort. Red symbols identify cysteine residues (n = 252) with p < 0.05 (non-paired, two-tailed Student’s t test), while blue symbols (n = 200) indicate a high-stringency significance (Benjamini-Hochberg test). Total unique peptides = 387. (D) Distribution of cysteine peptides plotted against redox states of the cysteine residues for catalase-overexpressing flies after 24-hr fasting. Shown is the mean for the relative numbers of cysteine residues in each 5% quantile of the five biological replicates. Dashed line is the distribution for untreated catalase-overexpressing flies on control food. (E) Oxidation state of cysteine residues in catalase-overexpressing flies upon 24 hr fasting against untreated cohorts. Dotted line slope = 1, while the continuous line is the best fit to the data. Each symbol represents a cysteine residue identified in at least three biological replicates of both the untreated and fasted cohorts. Red symbols identify cysteine residues (n = 96) with p < 0.05 (non-paired, two-tailed Student’s t test). Blue symbols (n = 51) indicate high-stringency significance assessed (Benjamini-Hochberg test). Total unique peptides = 440. (F) Oxidation state of cysteine residues present upon 24-hr fasting in catalase-overexpressing flies plotted against control flies. Dotted line slope = 1, while the continuous line is best fit to the data. Each symbol represents a cysteine residue that was identified in at least three biological replicates of both the fasted control and catalase-overexpressing flies. Red symbols identify cysteine residues (n = 13) p < 0.05 (non-paired, two-tailed Student’s t test). Total unique peptides = 601. See also Figures S5–S8.

To explore the effects of fasting on cysteine residue redox state, we fasted flies for 24 hr. Because the flies survived 7–10 days of fasting (Figure 5A), any redox events within 24 hr are an early adaptive response. Starting from young (7 days) control flies, fasting led to a substantial oxidation of cysteine residues (30.5%; Figure 5B). Comparing the redox state of individual cysteines after 24 hr fasting showed that there was a dramatic difference compared to fed flies (Figure 5C). This was due to oxidation of those cysteine residues that were largely reduced in fed, untreated controls, along with the reduction of cysteine residues that were oxidized in fed, untreated controls (Table S5).

The shifts in cysteine residue redox state during fasting could reflect changes in a particular cellular compartment. To investigate this, we looked at how cysteine residues on four complexes that span three different membranes responded to fasting. The cytoplasmic cysteine residues of the plasma membrane Na^+^/K^+^-ATPase became more oxidized during fasting (29.3% ± 1.2% versus 9.7% ± 1.3%), while the residues facing the extracellular environment became more reduced (46% ± 3.6% versus 80.4% ± 2.3%; Figure S5). Mitochondrial matrix cysteine residues on cytochrome *bc*-1 complex and cytochrome oxidase became more oxidized during fasting (37.1% ± 3.8% versus 9.5% ± 3.1%), whereas three cysteine residues that were observed as disulfides in the protein structures became more reduced (52.7% ± 0.6% versus 93.5% ± 3.2%; Figure S6). Finally, cytoplasmic cysteine residues of the sarcoplasmic/ER Ca^2+^-ATPase (SERCA) became more oxidized during fasting (31.2% ± 4.1% versus 17.7% ± 5.6%), while a cysteine residue that forms part of a disulfide in the ER lumen became more reduced (Figure S7). Taken together, it is clear that the redox changes observed during fasting were not confined to one compartment.

Because the cysteine-residue alterations suggested that redox changes occur during fasting, we next looked at the effect of catalase. Catalase overexpression slightly decreased survival in response to fasting compared to controls (Figure 4A). Most interestingly, catalase overexpression also reduced cysteine residues after 24 hr fasting (Figure 4D), and the weighted arithmetic mean of the cysteine-residue redox state (27.6%) was lower than for fasted controls. By plotting the redox state of individual cysteine residues under fasted conditions against fed, it was clear that catalase protected cysteine residues from oxidation during fasting, suggesting a role for H_2_O_2_ (Figures 4C, 4E, and 5F). In contrast, the reduction of oxidized cysteine residues was catalase insensitive, suggesting that this is H_2_O_2_ independent (Figures 4C, 5E, and 5F). Interestingly, fasting altered cysteine redox state to a greater extent than PQ treatment (Figure S8), although the patterns were qualitatively similar. Overall, these data are consistent with dramatic redox changes occurring rapidly upon fasting that lead to the cysteine-residue oxidation.

## Discussion

We assessed how cysteine-residue redox state changes within fruit flies in two situations: aging and fasting. The former is associated with physiological decline, while fasting for short periods (∼24 hr) leads to major metabolic changes. However, in both cases, the mechanisms are obscure and the role of redox changes to cysteine residues was not known. To address this, we used OxICAT to assess reversible redox changes in cysteine residues, enabling us to both assess the redox state of hundreds of cysteine residues simultaneously while also identifying the residues. To our surprise, we found that aging has no effect on cysteine-residue redox state. In stark contrast, fasting led to a dramatic reversible oxidation of protein thiols. These findings suggest that modulation of the redox state of cysteine residues is an early critical stage in the organism’s response to fasting.

The OxICAT approach gives a reasonable snapshot of the redox state of cysteine residues within living flies. From this, we can infer that the majority of cysteine residues are predominantly present as the free thiol (∼90% reduced), with a small number having undergone reversible oxidation so that they are ∼80%–90% oxidized. Many of the cysteine residues in the oxidized population are internal protein disulfides as they are in this form in the structures investigated (Figures S5–S7) or are on secreted proteins where we expect internal disulfides. However, the OxICAT approach cannot determine the nature of the modification, and many other reversible cysteine residue modifications are possible. Furthermore, this approach will not be able to pick up irreversibly oxidized or alkylated cysteine residues (e.g., sulfinic acids or thioethers), although there was no substantial loss of intensity as would be expected with widespread irreversible modification in PQ treatment and fasting. This distribution agrees with other redox proteomic studies, but it is possible that in vivo the cysteine residues are on average more reduced and that some oxidation occurs during preparation. Even if this is the case, the validity of relative changes in redox state remains.

The investigation of the effects of two exogenous forms of oxidative stress on protein cysteine redox state by OxICAT, H_2_O_2_ and PQ, provided intriguing and surprising results. The first was that even toxic levels of H_2_O_2_ did not alter protein thiol redox state. The interaction of H_2_O_2_ with protein thiols is an emerging area of redox signaling. Recently, it has become clear that this signaling is quite selective, consistent with the view that H_2_O_2_ reacts too slowly to affect many proteins but instead modifies highly reactive proteins such as peroxiredoxins (Prx) that then relay the change by thiol-disulfide exchange with target proteins (D’Autréaux and Toledano, 2007; Sobotta et al., 2015). This suggests, surprisingly, that even large amounts of H_2_O_2_ may have little impact on overall protein thiols, which are maintained in a reduced state even if the organism has undergone a fatal oxidative insult. However, we cannot exclude alternative possibilities, such as that the effect of H_2_O_2_ on the gut has a life-shortening impact that is not reflected in the bulk redox state, or that the cysteine-residue redox state recovers but the “damage” has been done, setting in motion the life-limiting processes.

In contrast to H_2_O_2_, the redox cycler PQ led to extensive oxidation of initially reduced cysteine residues, while at the same time reducing a substantial number of reversibly oxidized cysteine residues. This may in part be due to appetite suppression or inhibition of food consumption by PQ (Ja et al., 2007), which may mimic the increase in cysteine residue oxidation during fasting. In any case, these results contrast with the tacit assumption that different methods of increasing oxidative damage operate through broadly similar pathways. Therefore, it is clear that these two forms of oxidative stress cannot be used interchangeably nor their effects interpreted as being on the same pathway in studies of aging or oxidative damage.

There is a large body of evidence showing a correlation between oxidative damage and aging. Cysteine redox state has not been measured during aging in a multicellular organism. Despite the lack of evidence, there has been the unstated assumption that protein thiols would become more oxidized upon aging in parallel with other markers of oxidative damage. Surprisingly, when we measured cysteine-residue redox state there was no change with age. This suggests that the changes in oxidative damage that correlate with aging are not associated with changes in cysteine residues.

Fasting was markedly different from aging, as we found a dramatic increase in protein thiol oxidation after 24 hr, although the flies were perfectly viable for several days of fasting. The many changes that occur during fasting presumably arise from effects on the pentose phosphate shunt, NADP-dependent isocitrate dehydrogenase, and the NADH/NADPH transhydrogenase that supply electrons to maintain GSH and protein thiols reduced (Webster et al., 2014). However, the cysteine redox state and survival were both affected by catalase, suggesting that there was a component of H_2_O_2_ signaling involved. This could occur through upregulation of autophagy as fasting is known to induce autophagy, and this process is regulated by redox pathways (Aquilano et al., 2014). Thus, the dramatic shift in the redox state of cysteine residues during fasting may be associated with the activation of autophagy that provides nutrients to prolong survival.

This work extends our understanding of redox changes in major life processes—aging and fasting—in surprising ways and shows that redox processes are more subtle and complicated than suspected. It also opens up new technical approaches to investigate these changes in flies. Our results will surprise many in the aging field who have tacitly assumed that all forms of oxidative stress increase with age. There are also considerable implication for our understanding of the mechanistic details by which fasting and DR impact on health and lifespan. Our findings now redirect the field toward investigating the evanescent changes in protein redox state in response to diet and fasting, and future work will investigate the nature of the reversible modifications, their significance, and the cysteine residues affected. It will be particularly interesting to see whether these are associated with the dramatic and reversible shift in mortality with DR in flies (Mair et al., 2003; Robertson and Mitchell, 2013).

## Experimental Procedures

### Fly Husbandry

All experiments were performed with *white Dahomey* as wild-type background. The UAS-cat (Bloomington #24621) and da-GAL4 lines were backcrossed into the *white Dahomey* background for ten generations. Control (UAS-cat/+) and catalase-overexpressing (da-GAL4 > UAS-cat) females were used for experiments. For stress experiments, flies were maintained on standard sugar-yeast-agar food (SYA) for 7 days, then transferred to PQ medium, H_2_O_2_ medium, or fasting medium. Flies were collected by transferring to pre-chilled microtubes and flash freezing in liquid nitrogen, then stored at −80°C. Further details are provided in the Supplemental Experimental Procedures.

### Protein Isolation and OxICAT Peptide Preparation

To measure the redox state of protein cysteine residues using OxICAT, we used cohorts of ten female flies and rapidly froze them in liquid N_2_ (Figure 1B) and separated the frozen heads and thoraces from the abdomens on dry ice. Protein isolation, cysteine-residue labeling, peptide preparation, and protein thiol assays are described in the Supplemental Experimental Procedures.

### LC-MS/MS Analysis of Peptides

Liquid chromatography-tandem mass spectrometry (LC-MS/MS) analysis of the OxICAT-labeled peptides was carried out using an Orbitrap LTQ XL (Thermo) after chromatography on a nanoscale reverse-phase column (see Figure 1B). Each sample was run twice as a technical replicate, and five biological replicates were processed per experiment. Data analysis is described in the Supplemental Experimental Procedures.

## Author Contributions

K.E.M. performed the majority of the experiments and data analysis and drafted the manuscript with M.P.M. A.M.J. developed the procedures used and assisted with data analysis and manuscript preparation. H.M.C. carried out most of the fly work and assisted with manuscript preparation. M.E.H. helped with the analysis of the mass spectrometry data. E.T.C. helped optimize aspects of the OxICAT approach. S.D. carried out the mass spectrometric experiments under the supervision of I.M.F. L.P. and M.P.M. were the grant holders and provided leadership throughout. M.P.M. and L.P. directed the project. M.P.M. oversaw manuscript preparation.

## Accession Numbers

The mass spectrometry proteomics data have been deposited to the ProteomeXchange Consortium (Vizcaíno et al., 2014) via the PRIDE partner repository with the dataset identifier PXD002195.

## Supplemental Information

Document S1. Supplemental Experimental Procedures, Figures S1–S8, and Table S6

Table S1. List of Cysteine Residues Identified in This Study

Table S2. List of Cysteine Residues Whose Redox State Altered Significantly following the Indicated Interventions

Table S3. Cysteine Residues that Significantly Change Their Redox State upon Catalase Overexpression with Age, Assessed by a Benjamini-Hochberg Test

Table S4. Cysteine Residues that Significantly Change Their Redox State following Exposure to PQ, Assessed by a Benjamini-Hochberg Test

Table S5. Cysteine Residues that Significantly Change Their Redox State upon Fasting, Assessed by a Benjamini-Hochberg Test

Document S2. Article plus Supplemental Information
