# Fasting, but Not Aging, Dramatically Alters the Redox Status of Cysteine Residues on Proteins in *Drosophila melanogaster*

**DOI:** 10.1016/j.celrep.2015.10.048

**Published:** 2015-11-10

**Authors:** Katja E. Menger, Andrew M. James, Helena M. Cochemé, Michael E. Harbour, Edward T. Chouchani, Shujing Ding, Ian M. Fearnley, Linda Partridge, Michael P. Murphy

(Cell Reports *11*, 1856–1865; June 18, 2015)

In the Supplemental Information for the originally published version of this manuscript, Table S3 contained unrelated data for flies overexpressing catalase. The data have been removed, and the corrected version of the table now appears online with the paper.

The authors apologize for any confusion caused by this error.

