# Supplementary material for: Fasting, but Not Aging, Dramatically Alters the Redox Status of Cysteine Residues on Proteins in Drosophila melanogaster
Source: Cell Rep. 2015 Jun 18;11(12):1856–65. doi: 10.1016/j.celrep.2015.05.033 (PMC4508341; doi:10.1016/j.celrep.2015.05.033)
Supplement: Document S1. Supplemental Experimental Procedures, Figures S1–S8, and Table S6 [file mmc1.pdf]

Cell Reports

Supplemental Information

Fasting, but Not Aging, Dramatically Alters the Redox Status of Cysteine Residues on Proteins in *Drosophila melanogaster*

Katja E. Menger, Andrew M. James, Helena M. Cochemé, Michael Harbour, Edward T. Chouchani, Shujing Ding, Ian M. Fearnley, Linda Partridge, and Michael P. Murphy

SUPPLEMENTAL INFORMATION

SUPPLEMENTAL EXPERIMENTAL PROCEDURES

Fly Husbandry

All experiments were performed with *white Dahomey* as the wild-type background (negative for the endosymbiont *Wolbachia*), maintained in large population cages with outbreeding and overlapping generations at 25˚C on a 12 h light/12 h dark cycle. The UAS-cat (Bloomington #24621) and da-GAL4 (Wodarz et al., 1995) lines were back-crossed into the *white Dahomey* background for 10 generations. Flies were raised on standard sugar-yeast-agar medium (SYA) consisting of: 5% (w/v) sucrose (granulated sugar, Tate & Lyle), 10% (w/v) autolysed yeast (903312, MP Biomedicals), 1.5% (w/v) agar (Sigma), supplemented with 3% (v/v) nipagin and 0.3% (v/v) propionic acid as mould inhibitors, added once the food had cooled down to 60˚C (Grandison et al., 2009). Control (UAS-cat/+) and catalase over-expressing (da-GAL4 > UAS-cat) female flies were used for experiments. The control UAS-cat/+ flies have wild-type levels of catalase (mRNA and protein) and are equivalent to *white Dahomey*, whereas the ubiquitous catalase over-expressors showed ~6-fold up-regulation of catalase levels (parallel manuscript in preparation). All flies were reared at standard larval density in 200 ml bottles and eclosing adults were collected over a 12 h period. Flies were mated for 48 h before sorting into females, maintained at a density of 15 per vial and transferred to new food every 2-3 d. All experiments were incubated at 25˚C, 65% humidity on a 12 h light/12 h dark cycle.

For stress experiments, flies were maintained on standard SYA for 7 d, then transferred to paraquat (PQ) medium (20 mM PQ in SYA), H_2O_2 medium (5% H_2O_2 in 5% (w/v) sugar, 1.5% (w/v) agar), or fasting medium (1.5% (w/v) agar). Survival was scored by regularly counting the number of dead flies in each vial. In parallel, live flies were sampled after 24 h stress exposure. Flies were collected by transferring to pre-chilled microtubes and flash freezing in liquid nitrogen, and then stored at -80˚C until the time of experiment.

Protein Isolation and OxICAT Peptide Preparation

To measure the redox state of protein thiols within flies using OxICAT, we used cohorts of ten female flies, to allow for biological variation, and rapidly froze them in liquid N_2 (Figure 1B). We then separated the frozen heads and thoraces from the abdomens on dry ice (Cocheme et al., 2012) to avoid variation in egg production with age. Protein isolation is usually achieved by addition of 10-20% trichloroacetic acid (TCA) to prevent artifactual thiol oxidation and disulphide shuffling, while also precipitating proteins (Held and Gibson, 2012; Leichert et al., 2008; Zander et al., 1998). However, this was not feasible in flies due to the difficulty of separating the chitin exoskeleton from the precipitated protein. To overcome this, we homogenised frozen fly heads and thoraces in 100% (w/v) TCA to stabilise thiols and solubilise proteins (Rajalingam et al., 2009), which can then be separated from the insoluble exoskeleton. Heads and thoraces of ten flies in replicates of 5 were homogenised with 10 x 5 s pulses using a pestle to fit the microtubes in conjunction with a cordless pestle motor (VWR) in 200 μl 100% (w/v) ice-cold TCA. The homogenate was incubated on ice for 5 min, and chitin and other insoluble components were pelleted at 16000 g for 5 min. A tenth of the supernatant, equivalent to the protein present in the head and thorax of one fly, was transferred to a fresh microtube and the TCA concentration was reduced to 20% (w/v) by addition of H_2O to initiate protein precipitation. The homogenate was incubated on ice for 30 min and then pelleted for 30 min at 16000 g at 4˚C. This protein precipitation was achieved with minimal protein losses or distortion of the protein complement (Figure S1A). The pellet was washed with 10% and 5% (w/v) TCA and then resuspended in 80 μl denaturing alkylating buffer (DAB; 6 M urea, 2% (w/v) SDS, 200 mM Tris-HCl, 10 mM EDTA, 100 μM DTPA, 10 μM neocuprine). The contents of one vial of light ICAT reagent (AB SCIEX, USA 4339036) in 20 μl acetonitrile (ACN) was added to label reduced cysteine residues at 37˚C and 1400 rpm on an Eppendorf Thermomixer for 2 h. Proteins were then precipitated with 5 vols. of ice-cold acetone, incubated at -20˚C for 2 h, and pelleted at 4˚C and 16000 g for 30 min. The amount of protein to be processed for OxICAT analysis (~30-40 μg protein) was optimised to ensure saturation of thiol labelling by the light ICAT reagent (Figure S1B). The pellet was washed twice with 90%

ice-cold acetone and then solubilised in 80 µl DAB. The reducing agent *tris*(2-carboxyethyl)phosphine (TCEP) (1 mM, final concentration, 2 µl of a 50 mM stock, provided with the ICAT kit) was added, reducing previously reversibly oxidised cysteine residues, which were then labelled with one vial of heavy ICAT reagent (AB SCIEX, USA 4339036) dissolved in 20 µl ACN. Proteins were incubated at 37˚C and 1400 rpm on an Eppendorf Thermomixer for 2 h, and then precipitated with 5 vol ice-cold acetone, stored at -20˚C for 2 h and pelleted at 4˚C at 16000g for 30 min. The pellet was washed twice with 90% ice-cold acetone and then resuspended in denaturing buffer and 1 vol trypsin (resuspended in H_2O) was added (both provided by the ICAT kit, AB SCIEX, USA 4339036). Proteins were digested at 37˚C and 1400 rpm on an Eppendorf Thermomixer overnight. Digested peptides were enriched for cysteine-containing peptides first on a cation exchange cartridge and then subsequently on an avidin affinity cartridge (both provided with the ICAT kit, AB SCIEX, USA 4339036). Briefly, trypsinolysis is terminated by addition of 4 ml cation exchange buffer-*load* (10 mM KH_2PO_4, 25% ACN, pH 3.0) and the sample loaded onto the pre-equilibrated cation exchange cartridge. The flow-through was retained for potential trouble-shooting and the cartridge washed with 1 ml cation exchange buffer-*load*, with the flow through collected into the same tube as the loading flow through. The sample was eluted using 500 µl of cation exchange buffer-*elute* (10 mM KH_2PO_4, 25% ACN, 350 mM KCl, pH 3.0). The pH of the eluted sample was adjusted through the addition of 500 µl affinity buffer-*load* (2x PBS, pH 7.2 [1x PBS = 10 mM NaH_2PO_4, 150 mM NaCl]) and loaded onto a pre-equlibrated affinity cartridge. The flow through was collected for potential trouble-shooting and the cartridge washed with an additional 500 µl affinity buffer-*load* combined with the initial flow-through. To remove additional salts 1ml affinity buffer-*wash I* (1x PBS, pH 7.2) was added slowly and the flow-through discarded. Unlabelled peptides were eluted from the cartridge with 1 ml affinity buffer-*wash II* (50 mM ammonium bicarbonate [NH_4HCO_3]/ 20% methanol, pH 8.3) and the flow-through collected. ICAT labelled peptides are eluted after washing the cartridge with 1 ml H_2O using 800 µl affinity buffer-*elute*, (30% (v/v) ACN, 0.4% (w/v) trifluoroacetic acid (TFA), with the first 50 µl eluate being discarded. The eluted peptides were dried down overnight in a Speed vac and the biotin moiety of the ICAT label was removed by

incubation with the cleaving reagents provided in the ICAT kit (2 h at 37˚C and 1400 rpm on an Eppendorf Thermomixer). The isolated peptides were then dried down in a speed vac.

Mass Spectrometric Data Analysis

Raw files for each LC-MS/MS run were analysed using MaxQuant software to determine the ratio of heavy over light OxICAT-labelled peptides in our experiments (Cox and Mann, 2008). Raw files from the two technical replicates obtained during two LC-MS/MS runs of the same biological replicate were grouped to provide a dataset for that biological sample. As possible modifications, besides the labelling of cysteine residues with light or heavy ICAT label, only methionine oxidation was included and two possible missed cleavages were allowed in the MaxQuant search parameters (listed in Table S6). As a reference sequence database, a FASTA file containing all protein sequences associated with *D. melanogaster* was downloaded from www.uniprot.org. (July 2011). This reference sequence database was used by the search engine Andromeda associated with MaxQuant to identify peptides detected during the LC-MS/MS run. In addition, the reference sequence database was used to create a list of all cysteine-containing tryptic peptides within the *D. melanogaster* proteome. To do this, an *in silico* tryptic digest of the complete proteome was performed using a modified proteogest script (Cagney et al., 2003), generating a file that listed all cysteine-containing tryptic peptides, with none, one or two missed cleavages, the cysteine residue number and the Uniprot accession number of the protein (Cagney et al., 2003). The proteogest script modification was done by Dr Alan Robinson, MRC Mitochondrial Biology Unit. Ratios of heavy over light ICAT labelled peptides listed in the evidence file obtained from MaxQuant were converted to % of the cysteine residue that had been reversibly oxidised. Using the SQL database software pgAdmin3, the peptide list generated by proteogest was used together with the modified evidence file to combine intensity from all peptide signals containing the cysteine residue of interest (i.e. miscleaved, methionine oxidation, different *z* values). This generated a mean % reversible oxidation for each unique cysteine residue identified within the biological replicates. We used the $\log_{10}$ intensity to generate averages of the abundance of the peptides with unique cysteine residues across 3 to 5 biological

replicates and across all amino acid sequences associated with the cysteine residue identified. The threshold of log_{10} intensity ~5-6 for peptide pair identification is due to the limit for detection by the mass spectrometer and is variable because the detection of individual peptides depends on sequence, abundance and co-eluting peptides.
The % reversible oxidation and the summed eXtracted Ion Current (XIC: defined as the summed eXtracted Ion Current of all isotopic clusters associated with the identified peptide sequence, averaged across the biological replicates, also used for the log_{10} intensity) remained associated during information processing. Information on the subcellular localisation of the proteins identified and the biological processes they participated in was obtained using MitoMiner (http://mitominer.mrc-mbu.cam.ac.uk/) (Smith and Robinson, 2009).

For bar charts, the data for distribution of proportion of cysteine residues against % oxidation were generated by counting the number of cysteine residues in each 5% quantile for each individual biological replicate and then averaging the relative values for each quantile over the 5 biological replicates. The fitted red curves are the data from related experiments fitted manually using a Lorentizian equation to the distribution that is re-plotted to facilitate assessment of changes.

For plots of the % oxidation of a given cysteine under one condition against its % oxidation under another condition, those peptides for which there was a shift in % oxidation assessed by an uncorrected Student's t test were plotted in red. While many of these differences will be real because of the large dataset there will also be a significant number of false positives. Therefore this gives a low-stringency visual indication of shifts in % oxidation between conditions as well as to allow other studies to definitively corroborate them by orthogonal techniques. To assess those shifts in % oxidation that can be more strongly interpreted as a shift for that cysteine residue solely from our data, we used the Benjamini-Hochberg procedure (Benjamini and Hochberg, 1995; Hochberg and Benjamini, 1990) to correct for multiple comparisons and these data are shown in blue. It has to be noted that every cysteine residue that is found to change significantly according to the Benjamini-Hochberg procedure will also have passed the level of significance in the Student's t-test.

The program pgAdmin3 was used to link existing transcriptional abundance datasets (Chintapalli et al., 2007) with the list of cysteines observed by OxICAT. Briefly, Uniprot tags (Affymetrix; Drosophila_2.na33.annot.csv) were annotated to whole fly microarray data (FlyAtlas; 20090519.txt) using probe identifications common between the two datasets. Common Uniprot tags and sequence data were then used to annotate the transcriptional information in this dataset to a theoretical trypsin digest of the whole fly proteome as well as the subset of cysteines we observed by OxICAT. This provided expression levels for each cysteine observed by OxICAT that could be compared to that of the complete fly proteome.

Protein Thiol Assays

To measure bulk protein thiols, the heads and thoraces from 20 flies were homogenised using a pestle fitted to a microtube and a cordless pestle motor (VWR) in 100 µl 100% (w/v) ice-cold TCA. The homogenate was incubated on ice for 5 min, and then chitin and other insoluble components were pelleted at 16000 g and 4˚C for 5 min. The supernatant was transferred to a fresh tube and the TCA concentration was reduced to 20% (w/v) through the addition of H_2O and the sample was incubated on ice for 30 min. The protein precipitate was pelleted by centrifugation at 16000 g and 4˚C for 30 min. The supernatant was removed and the pellet washed with 500 µl 10 % and 5 % (w/v) TCA. The pellet was resuspended in 250 µl sample buffer (20 mM Tris, 10 mM EDTA, pH 7.3 with 1 % (w/v) n-dodecyl-maltoside (DDM)), incubated on ice for 30 min and the sample was split into aliquots of 75 µl and 150 µl. The 150 µl aliquot was reduced by treating with 1 mM dithiothreitol (DTT), then split further into two 75 µl aliquots, and SDS (final concentration 2% (w/v)) was added to one of them. All aliquots were then incubated at RT for 10 min. To remove small thiol-containing molecules all aliquots were passed twice through MicroBioSpin6 columns (6 kDa; BioRad), pre-equilibrated with sample buffer. Then 10 µl of the sample or GSH standards were mixed with 160 µl assay buffer (80 mM NaH_2PO_4, 1 mM EDTA, pH 8) ± 200 µM 5,5'-dithiobis-(2-nitrobenzoic acid) (DTNB). The samples were incubated at RT for 30 min and difference in absorbance between the ± DTNB samples and standards was measured at λ_{412}. The thiol concentration of the protein samples was determined from the GSH standard curve. All

measurements were done in triplicate. In parallel the protein concentration of each aliquot was determined using the BCA assay. This allows the calculation of thiols per mg protein for either the reduced exposed protein thiols (not treated with DTT or SDS), the total exposed thiols (treated with DTT but not with SDS) or the total thiols (treated with DTT and SDS) (Requejo et al., 2010).

SUPPLEMENTAL REFERENCES

Benjamini, Y., and Hochberg, Y. (1995). Controlling the false discovery rate: a practical and powerful approach to multiple testing. J. Royal Stat. Soc., Series B *57*, 289-300.

Cagney, G., Amiri, S., Premawaradena, T., Lindo, M., and Emili, A. (2003). *In silico* proteome analysis to facilitate proteomics experiments using mass spectrometry. Proteome Sci. *1*, 5.

Chintapalli, V.R., Wang, J., and Dow, J.A. (2007). Using FlyAtlas to identify better *Drosophila melanogaster* models of human disease. Nat. Genet. *39*, 715-720.

Cochemé, H.M., Logan, A., Prime, T.A., Abakumova, I., Quin, C., McQuaker, S.J., Patel, J.V., Fearnley, I.M., James, A.M., Porteous, C.M., *et al.* (2012). Using the mitochondria-targeted ratiometric mass spectrometry probe MitoB to measure H_2O_2 in living *Drosophila*. Nat. Prot. *7*, 946-958.

Cox, J., and Mann, M. (2008). MaxQuant enables high peptide identification rates, individualized p.p.b.-range mass accuracies and proteome-wide protein quantification. Nat. Biotech. *26*, 1367-1372.

Grandison, R.C., Wong, R., Bass, T.M., Partridge, L., and Piper, M.D. (2009). Effect of a standardised dietary restriction protocol on multiple laboratory strains of *Drosophila melanogaster*. PLoS One *4*, e4067.

Held, J.M., and Gibson, B.W. (2012). Regulatory control or oxidative damage? Proteomic approaches to interrogate the role of cysteine oxidation status in biological processes. Mol. Cell. Proteom. *11*, R111 013037.

Hochberg, Y., and Benjamini, Y. (1990). More powerful procedures for multiple significance testing. Stat. Med. *9*, 811-818.

Leichert, L.I., Gehrke, F., Gudiseva, H.V., Blackwell, T., Ilbert, M., Walker, A.K., Strahler, J.R., Andrews, P.C., and Jakob, U. (2008). Quantifying changes in the thiol redox proteome upon oxidative stress *in vivo*. Proc. Natl. Acad. Sci. USA *105*, 8197-8202.

Rajalingam, D., Loftis, C., Xu, J.J., and Kumar, T.K. (2009). Trichloroacetic acid-induced protein precipitation involves the reversible association of a stable partially structured intermediate. Protein Sci. *18*, 980-993.

Requejo, R., Hurd, T.R., Costa, N.J., and Murphy, M.P. (2010). Cysteine residues exposed on protein surfaces are the dominant intramitochondrial thiol and may protect against oxidative damage. FEBS J. *277*, 1465-1480.

Smith, A.C., and Robinson, A.J. (2009). MitoMiner: an integrated database for the storage and analysis of mitochondrial proteomics data Mol. Cell Proteom.

Wodarz, A., Hinz, U., Engelbert, M., and Knust, E. (1995). Expression of crumbs confers apical character on plasma membrane domains of ectodermal epithelia of *Drosophila*. Cell *82*, 67-76.

Zander, T., Phadke, N.D., and Bardwell, J.C. (1998). Disulfide bond catalysts in *Escherichia coli*. Meth. Enzymol. *290*, 59-74.

Figure S1

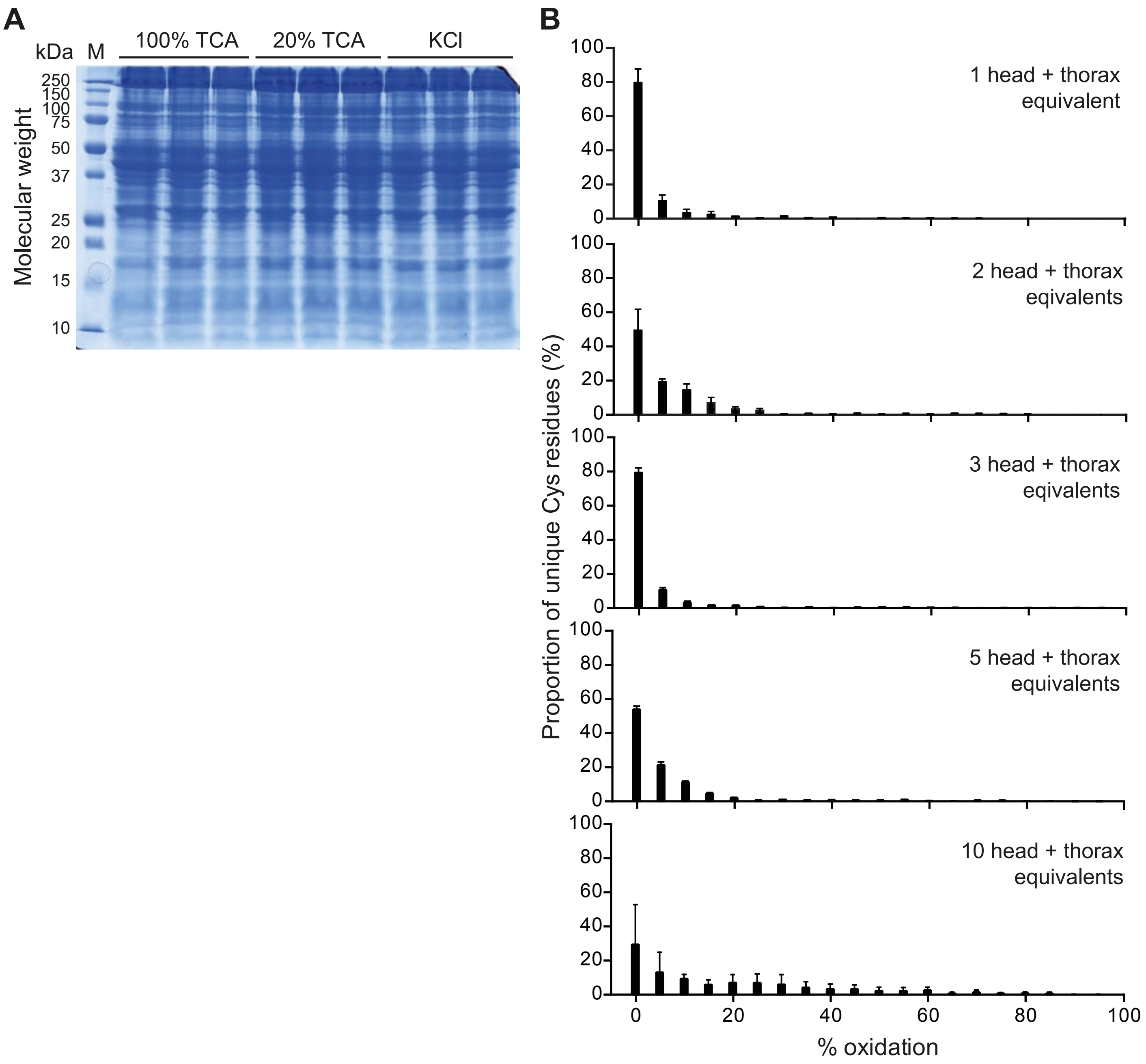
A
kDa
M
100% TCA
20% TCA
KCl
250
150
100
75
50
37
25
20
15
10
Molecular weight
B
1 head + thorax equivalent
2 head + thorax eqivalents
3 head + thorax eqivalents
5 head + thorax equivalents
10 head + thorax equivalents
Proportion of unique Cys residues (%)
% oxidation
0
20
40
60
80
100

Figure S1. Assessment of Protein Yield During OxICAT Experiments

(A) Determination of protein yield upon TCA precipitation of fly homogenates. Cohorts of young (7 d) female control flies were split into nine groups of 10 heads and thoraces. Samples were then homogenised in triplicate in either 100% (w/v) TCA, 20% (w/v) TCA, or KCl buffer (120 mM KCl, 3 mM Hepes-KOH, 1 mM EGTA, pH 7.5). The 100% TCA sample was incubated on ice for 5 min and insoluble components were pelleted by centrifugation at 16000 g, 4ºC for 5 min. The supernatant was transferred to a fresh tube and the TCA concentration lowered to 20% through the addition of H_2O. Insoluble components in the KCl buffer were pelleted by centrifugation at 16000 g, 4ºC for 5 min, and the supernatant transferred to a fresh tube where 100% TCA was added to result in a final concentration of 20% TCA. Homogenisation of the sample in 20% TCA caused protein precipitation straight away so the pellet was dissolved in loading buffer but chitin was largely retained in the bottom. All samples were incubated on ice for 30 min and the protein pelleted by centrifugation at 16000 g, 4ºC for 30 min. The pellets were washed with 500 µl 10% TCA followed by a 5% TCA wash. Proteins were resuspended in 100 µl SDS loading buffer without bromophenol blue and the protein concentration determined with the BCA assay. The protein concentration in 100% TCA, 20% TCA and KCl was 2.37 ± 0.15 mg/ml, 3.02 ± 0.04 mg/ml and 2.82 ± 0.04 mg/ml, respectively (n = 3). The 100% TCA showed a 16-21% lower amount of protein than the other methods, probably due to difficulty of eliminating keratin carryover in the 20% TCA and KCl experiments. After bromophenol was added equal amounts of 40 µg protein in 20 µl were loaded in triplicate onto a pre-cast 12% gel. The gel was run at 110 V at RT until the dye front reached the bottom. The gel was stained with coomassie at RT for 30 min and then destained, showing very similar patterns of protein distribution.

(B) Optimisation of the number of flies needed. In order to determine how efficient the ICAT labelling is with different amounts of protein a preliminary labelling with different levels of fly protein was performed. For this 10 heads and thoraces were homogenised in 100% (w/v) TCA and incubated on ice for 5 min. Insoluble components like chitin were pelleted by centrifugation and the equivalent of (top to bottom) one, two, three, five, or ten heads and thoraces was taken from the supernatant and transferred to a fresh tube with the appropriate amount of H_2O to lower the TCA concentration to 20% (w/v) TCA. The protein was pelleted and then solubilised in denaturing buffer with the reducing agent TCEP present. This reduces all previously oxidised cysteine residues. Full labelling should therefore be achieved where the majority of thiols are labelled with light ICAT reagent. After reaction with the light ICAT label, proteins were precipitated with acetone and subsequently solubilised in denaturing buffer with reducing agent TCEP and heavy ICAT label present to label all non-labelled cysteine residues. To remove excess label the proteins were precipitated by acetone and then digested by trypsin. Enrichment of the ICAT labelled peptides with an avidin column was followed by LC-MS/MS analysis. The fact that the labelling with light ICAT reagent decreases with increasing number of flies indicates that there is not enough light ICAT reagent to saturate all available thiols and therefore the protein equivalent of one fly head and thorax was used but maintaining the praxis of homogenising ten heads and thoraxes to preserve biological variability.

SFig 1 is linked to Experimental procedures.

Figure S2

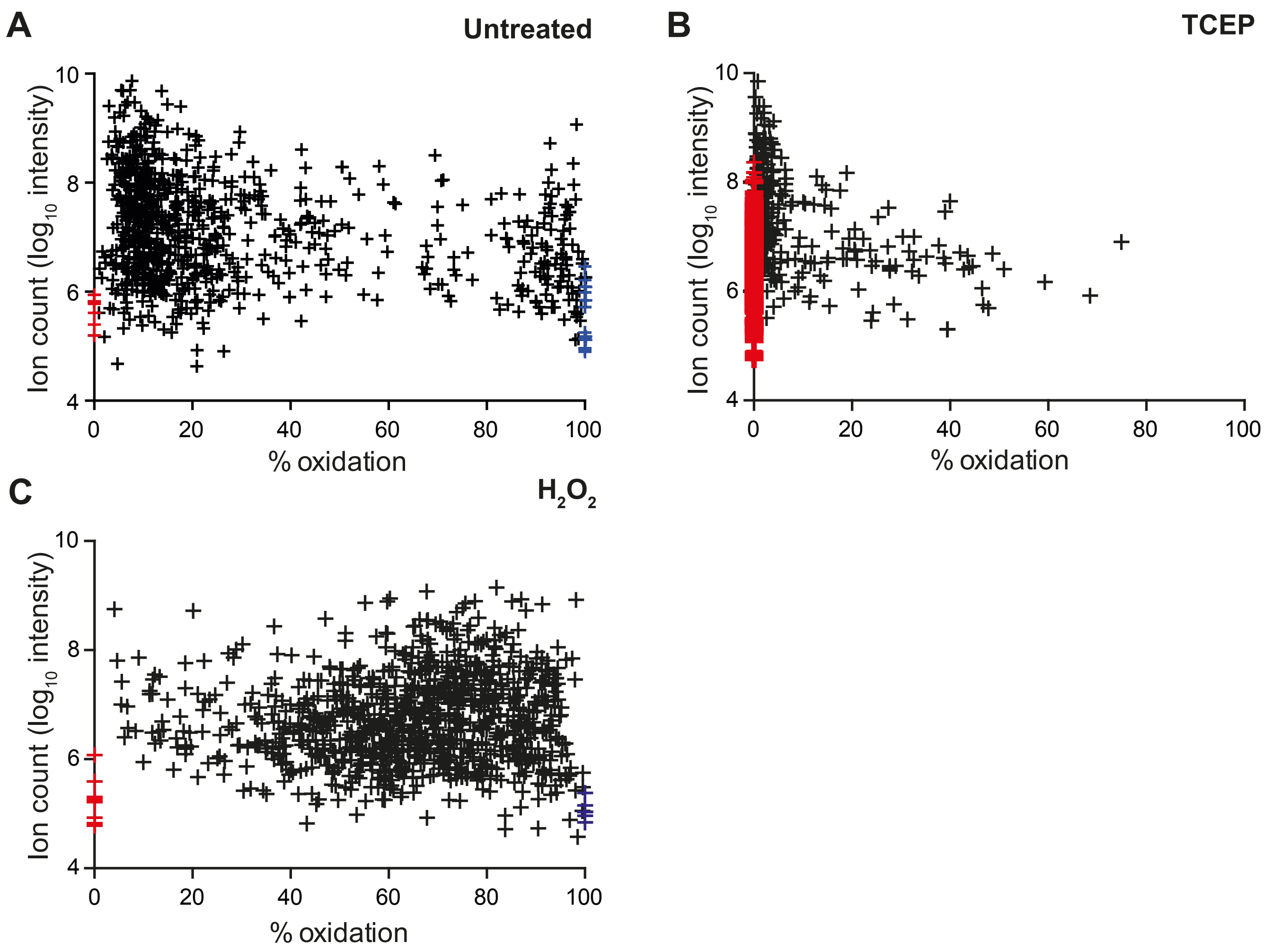

Figure S2. OxICAT Analysis of Young (7 d) Female Control *D. melanogaster*

(A) The data from Fig 2A are re-plotted to include peptides for which only the heavy labelled peptide (blue = 100% oxidised; n = 14) or light labelled peptide (red = 100% reduced; n = 6) was observed. For this the ion count for particular peptides is plotted against the % oxidation of the cysteine residue. Total unique peptides = 862.

(B) The fly homogenate prepared in the same way as in Figure 2A was treated with 1 mM TCEP in the presence of light ICAT regent (for 2 h at 37ºC) to reduce all reversible thiols, and then processed for OxICAT as usual. Red = 100% reduced; n = 434. Total unique peptides = 748.

(C) The fly homogenate prepared in the same way as in Fig 2A was treated with 5 mM H_2O_2 for 10 min to increase the oxidation of protein thiols, and then processed for OxICAT as usual. Blue = 100% oxidised; n = 6. Red = 100% reduced; n = 12. Total unique peptides = 1016.

SFig 2 is linked to Fig 2.

Figure S3

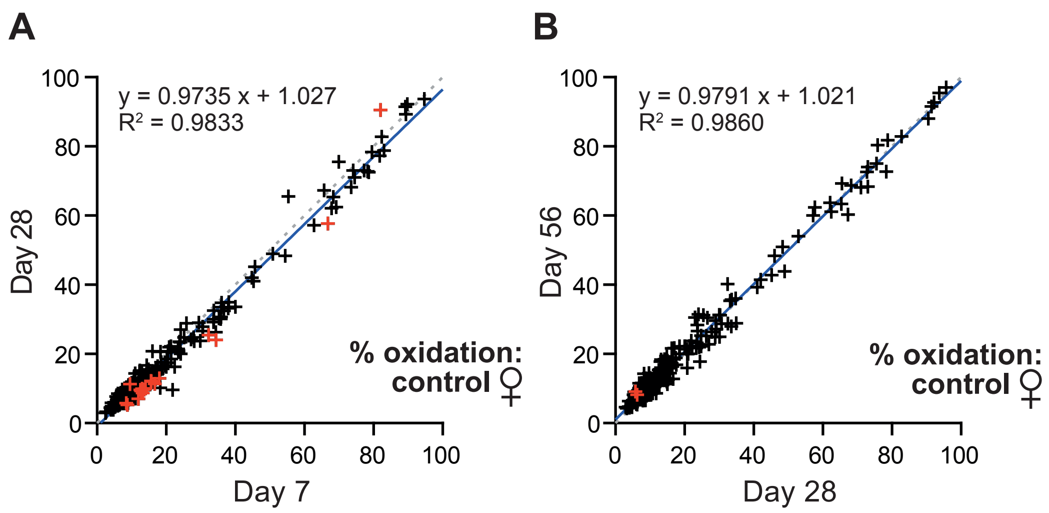

Figure S3. No Change in Protein Cysteine Residue Redox State with Age in Control *D. melanogaster*

(A) Oxidation state of cysteine residues present in control middle aged (28 d) flies plotted against young (7 d) flies. The dotted line has a slope of 1, while the continuous line is the least squares best-fit line to the data, with the equation for the line and Pearson's correlation coefficient indicated. Red symbols (n = 18) indicate a low stringency significance with a p-value smaller than 0.05 assessed by a two-tailed, non-paired Student's t-test. No data points had a high stringency significance assessed by a Benjamini-Hochberg test. Total unique peptides = 268.

(B) Oxidation state of cysteine residues present in control old (56 d) flies plotted against middle aged (28 d) flies. The dotted line has a slope of 1, while the continuous line is the least squares best-fit line to the data, with the equation for the line and Pearson's correlation coefficient indicated. Red symbols (n = 2) indicate a low stringency significance with a p-value smaller than 0.05 assessed by a two-tailed, non-paired Student's t-test. No data points had a high stringency significance assessed by a Benjamini-Hochberg test. Total unique peptides = 276.

SFig 3 is linked to Fig 3.

Figure S4

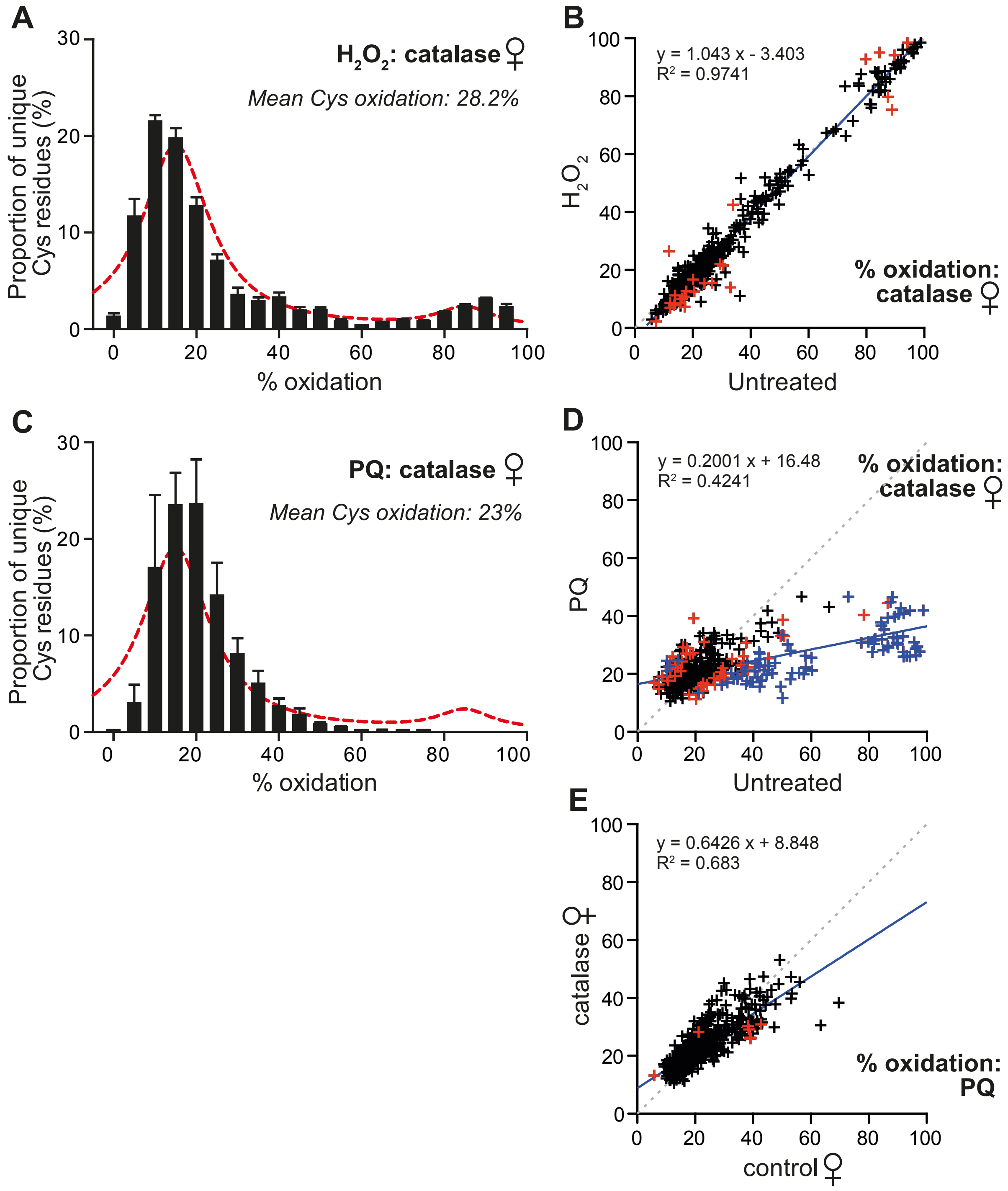

A
H2O2: catalase ♀
Mean Cys oxidation: 28.2%
Proportion of unique Cys residues (%)
% oxidation
B
y = 1.043 x - 3.403
R² = 0.9741
H2O2
Untreated
% oxidation: catalase ♀
C
PQ: catalase ♀
Mean Cys oxidation: 23%
Proportion of unique Cys residues (%)
% oxidation
D
y = 0.2001 x + 16.48
R² = 0.4241
% oxidation: catalase ♀
PQ
Untreated
E
y = 0.6426 x + 8.848
R² = 0.683
catalase ♀
% oxidation: PQ
control ♀

Figure S4. Effect of H_2O_2 and PQ Treatment on Cysteine Residue Redox State in Catalase Over-expressing Flies

(A) Distribution of total cysteine residue oxidation levels in catalase over-expressing flies exposed to dietary H_2O_2 (24 h treatment). Plotted is the proportion of the total number of peptides containing unique cysteine residues in each 5% quantile of % oxidation (mean ± SEM). The weighted arithmetic mean of the protein cysteine residue oxidation is indicated. The dashed red line is the manually fitted distribution using a Lorentizian equation for the untreated catalase over-expressing cohort.

(B) Oxidation state of cysteine residues present in catalase over-expressing flies exposed to H_2O_2 plotted against untreated flies. The dotted line has a slope of 1, while the continuous line is the least squares best-fit line to the data, with the equation for the line and Pearson's correlation coefficient indicated. The red symbols (n = 26) indicate cysteine residues with a p value smaller than 0.05 following a non-paired, two-tailed Student's t-test. No data points had a high stringency significance assessed by a Benjamini-Hochberg test. Total unique peptides = 485.

(C) Distribution of total protein thiol oxidation levels in young (7 d) catalase over-expressing flies exposed to PQ. Plotted is the proportion of the total number of peptides containing unique cysteine residues in each 5% quantile of % oxidation (mean ± SEM). The weighted arithmetic mean of the protein thiol oxidation is indicated. The dashed red line is the manually fitted distribution using a Lorentizian equation for the untreated catalase over-expressing cohort.

(D) Oxidation state of cysteine residues present in catalase over-expressing flies exposed to PQ plotted against untreated flies. The dotted line has a slope of 1, while the continuous line is the least squares best-fit line to the data, with the equation for the line and Pearson's correlation coefficient indicated. The red symbols (n = 139) indicate cysteine residues found to be significantly different using the less stringent non-paired two-tailed Student's t-test with a p value < 0.05. Blue symbols (n = 85) indicate cysteine residues found to be significantly different using the more stringent Benjamini-Hochberg-test. Total unique peptides = 429.

(E) Oxidation state of cysteine residues upon PQ treatment of catalase over-expressing flies plotted against control flies. The dotted line has a slope of 1, while the continuous line is the least squares best-fit line to the data, with the equation for the line and Pearson's correlation coefficient indicated. The red symbols (n = 8) indicate cysteine residues with a p value smaller than 0.05 following a non-paired, two-tailed Student's t-test. No data points had a high stringency significance assessed by a Benjamini-Hochberg test. Total unique peptides = 534.

SFig 4 is linked to Fig 4.

Figure S5

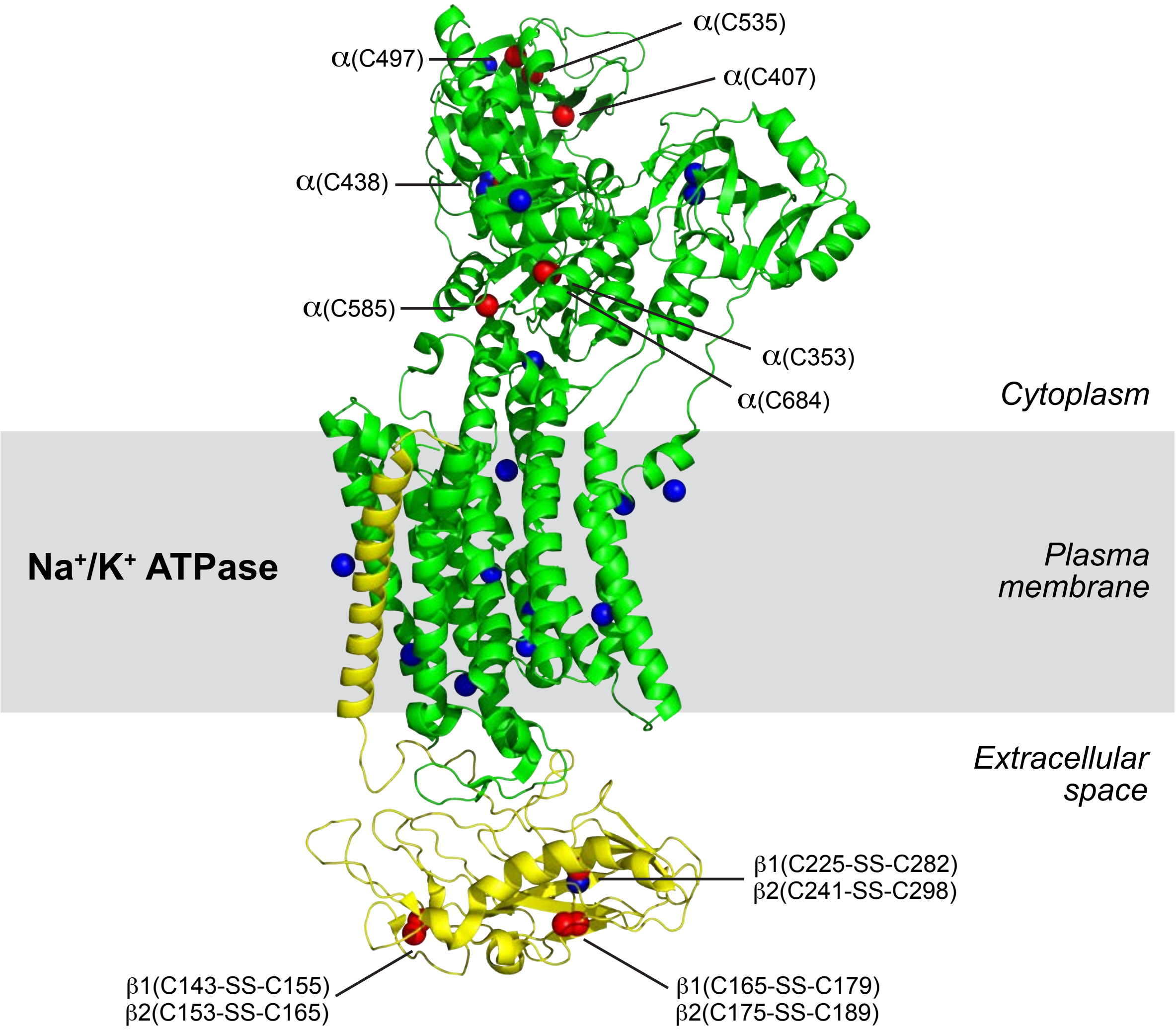

Subunit (Uniprot)	Cys residue	Control (% oxidation)				Catalase (% oxidation)			
		Untreated	H_2O_2	PQ	Fasting	Untreated	H_2O_2	PQ	Fasting
α(E1JIR4)	240	4.1 (5)	4.6 (5)	15.3 (5)	28.7 (5)	8.5 (5)	6.3 (5)	19.2 (5)	24.6 (5)
α(E1JIR4)	353	8.6 (5)	10.6 (5)	17.3 (5)	26.2 (5)	14.4 (5)	13.8 (5)	18.4 (5)	22.9 (5)
α(E1JIR4)	407	6.2 (5)	7.0 (5)	10.9 (5)	27.2 (5)	18.6 (5)	11.0 (5)	17.3 (5)	16.1 (5)
α(E1JIR4)	438	14.4 (5)	15.3 (5)	15.3 (5)	28.1 (4)	14.9 (5)	20.8 (5)	17.2 (4)	25.8 (5)
α(E1JIR4)	497	5.0 (5)	4.6 (4)	13.0 (5)	33.4 (5)	7.3 (5)	5.0 (3)	14.8 (5)	23.3 (5)
α(E1JIR4)	535	14.2 (5)	15.7 (5)	19.2 (4)	35.9 (5)	14.7 (5)	15.1 (5)	18.5 (2)	21.7 (4)
α(E1JIR4)	585	8.7 (1)	8.5 (3)					22.4 (2)	33.9 (1)
α(E1JIR4)	684	4.6 (5)	5.9 (5)	13.5 (5)	25.3 (5)	12.2 (5)	7.0 (5)	15.6 (5)	21.2 (5)
β1(Q24046)	143,155,165		81.3 (3)		30.4 (1)	77.1 (2)	69.7 (4)	18.0 (2)	15.8 (1)
β1(Q24046)	179			32.8 (5)	39.0 (5)		79.1 (1)	29.9 (1)	24.5 (2)
β1(Q24046)	282		88.0 (2)	39.9 (3)	75.8 (1)	87.0 (2)	80.3 (3)		
β2(Q24048)	8	20.2 (5)	18.2 (5)	32.4 (2)	29.8 (3)	23.9 (5)	15.1 (5)	27.7 (4)	33.6 (5)
β2(Q24048)	153			36.1 (5)	42.6 (4)		83.2 (1)	26.8 (2)	27.7 (2)
β2(Q24048)	153,165	81.1 (1)	78.7 (4)	48.9 (3)	55.2 (2)	87.4 (3)	79.8 (2)	44.8 (4)	41.3 (4)
β2(Q24048)	165	82.5 (1)	82.2 (1)	33.0 (5)	46.4 (5)	77.3 (3)	84.5 (4)	30.8 (4)	23.4 (3)
β2(Q24048)	175	83.6 (2)	76.1 (3)	32.8 (4)	30.0 (1)			29.0 (4)	30.9 (3)
β2(Q24048)	189	71.3 (1)	70.1 (5)		41.7 (3)	69.6 (4)	68.7 (5)	26.5 (2)	
β2(Q24048)	298	83.5 (1)	83.4 (3)	38.6 (1)	56.8 (3)	86.3 (2)	72.6 (2)	47.0 (2)	37.9 (4)
β3(Q7JS69)	34	23.3 (2)	35.8 (2)	19.4 (5)	29.9 (5)	26.3 (2)		20.6 (3)	17.6 (4)

Figure S5. The Redox Changes Observed During Stress Occur Across the Plasma Membrane

Plasma membrane Na^+/K^+ ATPase. *D. melanogaster* Na^+/K^+ ATPase contains two subunits, an α and a β subunit, with the β subunit having three isoforms, β1, β2 and β3. The monomeric structure of Na^+/K^+ ATPase from *S. acanthias* (2ZXE) contains two subunits: subunit α (green) is 77% homologous to the α subunit of *D. melanogaster* and subunit β1 (yellow) is 31%, 26% and 30% homologous to the β1, β2 and β3 subunits, respectively, of *D. melanogaster*. The sulphur atom of cysteine residues on the *S. acanthias* structure that are observed by OxICAT are shown as red spheres and numbered according to the *D. melanogaster* sequence. The sulphur atom of cysteines that are not detected by OxICAT are blue spheres. If they are present as a disulphide, their cysteine partner is also labelled. The table shows the oxidation state of each cysteine under different conditions with the number of times it was observed in parentheses.

SFig 5 is linked to Fig 5.

Figure S6

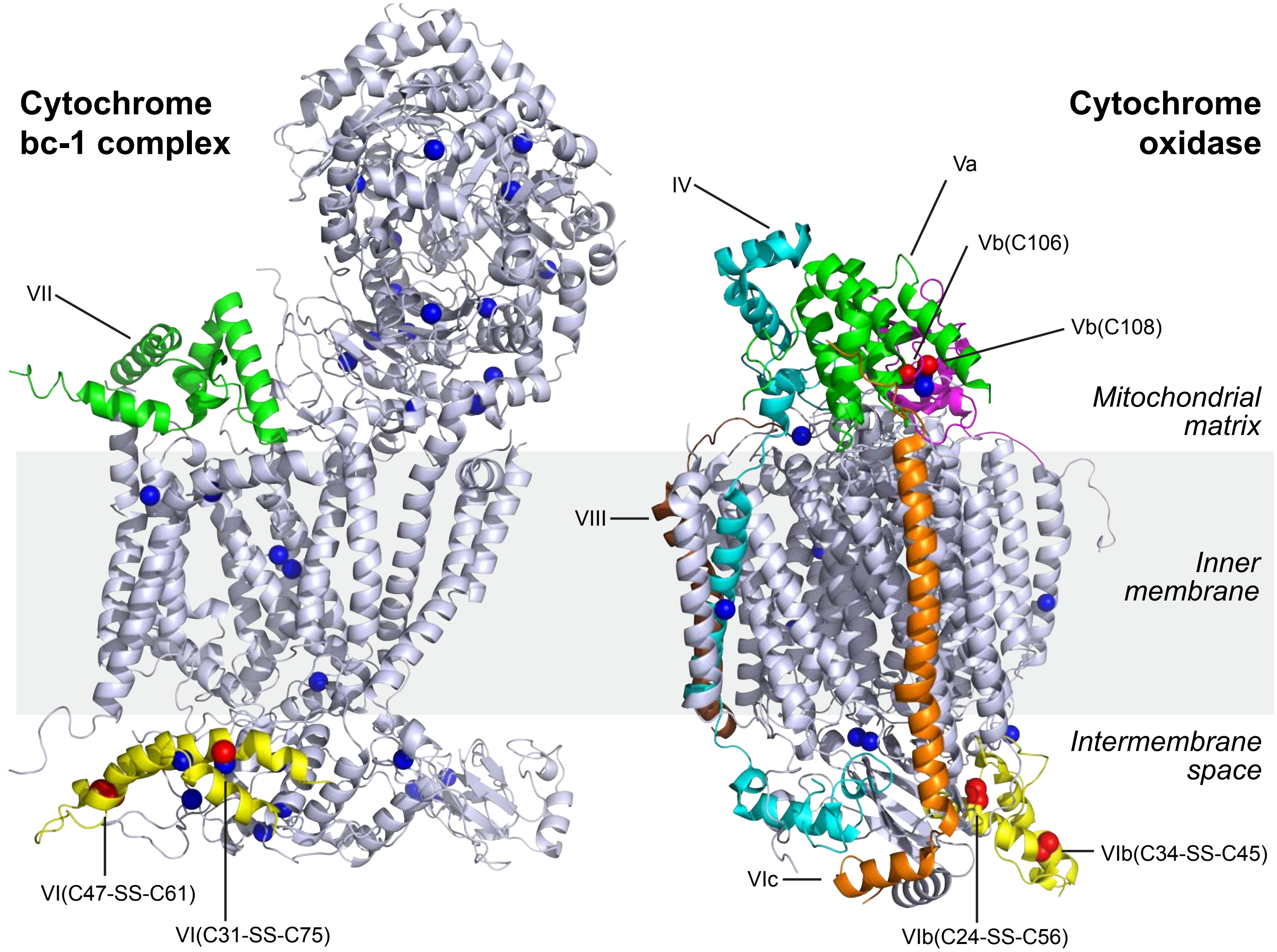

	Subunit (Uniprot)	Cys residue	Control (% oxidation)				Catalase (% oxidation)			
			Untreated	H_2O_2	PQ	Fasting	Untreated	H_2O_2	PQ	Fasting
**Cytochrome bc-1 complex**	VI (A8Y535)	47	94.7 (4)	94.2 (5)	37.1 (5)	52.1 (5)	91.9 (5)	92.7 (5)	40.9 (5)	50.0 (5)
	VI (A8Y535)	61 75		95.4 (2)			92.5 (3)	96.1 (4)		
	VII (Q9VXI6)	36	7.2 (5)	7.8 (5)	21.3 (5)	37.6 (5)	10.9 (5)	9.7 (4)	22.5 (4)	28.2 (4)
**Cytochrome oxidase**	IV (Q9VIQ8)	51	5.7 (5)	6.7 (5)	17.3 (5)	30.3 (5)	13.0 (5)	15.6 (5)	23.2 (5)	19.9 (5)
	IV (Q9VIQ8)	100			38.1 (4)	29.7 (3)			57.5 (2)	37.0 (4)
	Va (Q94514)	100	15.7 (3)	18.6 (4)	22.5 (5)	43.3 (5)	21.5 (5)	18.4 (5)	33.0 (5)	32.4 (4)
	Va (Q94514)	100 104	18.4 (5)	14.7 (5)	27.5 (4)		19.3 (5)	17.6 (5)	39.2 (3)	41.3 (4)
	Vb (Q9VMB9)	106 108	26.0 (3)	29.4 (4)			46.2 (1)			
	VIb (Q8IQW2)	45	87.5 (4)	89.6 (5)	42.3 (5)	52.1 (5)	90.2 (5)	89.5 (5)	40.7 (5)	47.7 (5)
	VIb (Q8IQW2)	24 34	93.4 (2)	86.1 (4)			72.6 (2)	79.9 (1)	50.0 (1)	78.6 (1)
	VIb (Q8IQW2)	24	98.3 (4)	97.4 (5)	42.5 (5)	53.9 (5)	98.8 (5)	98.6 (5)	42.0 (5)	47.4 (5)
	VIb (Q8IQW2)	56			43.5 (4)				42.7 (1)	44.1 (3)
	Vic (Q9VMS1)	77	6.6 (5)	4.9 (5)			6.6 (3)	4.4 (2)	28.9 (2)	59.3 (1)
	VIII (Q9VP19)	47		23.1 (4)			26.7 (3)	22.1 (1)		

Figure S6. The Redox Changes Observed During Stress Occur Within Mitochondria

Bos taurus cytochrome bc1 complex (1BE3) spans the mitochondrial inner membrane and contains 11 subunits. In the OxICAT experiments *D. melanogaster* cysteine-containing peptides were observed from subunits VI (yellow) and VII (green) which are 38% and 56% homologous to the bovine sequences. The structure of *B. taurus* cytochrome oxidase (3ASO) also spans the mitochondrial inner membrane and contains 13 subunits with cysteine-containing peptides observed from subunits IV (cyan), Va (green), Vb (magenta), VIb (yellow), VIc (orange) and VIII (brown) which are 34%, 53%, 44%, 56%, 37% and 23% homologous to their respective subunits in *D. melanogaster*. The sulphur atom of cysteine residues on the *B. taurus* structure that are observed by OxICAT are shown as red spheres and numbered according to the *D. melanogaster* sequence. The sulphur atom of cysteines that are not detected by OxICAT are blue spheres. If they are present as a disulphide, their cysteine partner is also labelled. The table shows the oxidation state of each cysteine under different conditions with the number of times it was observed in parentheses.

SFig 6 is linked to Fig 5.

Figure S7

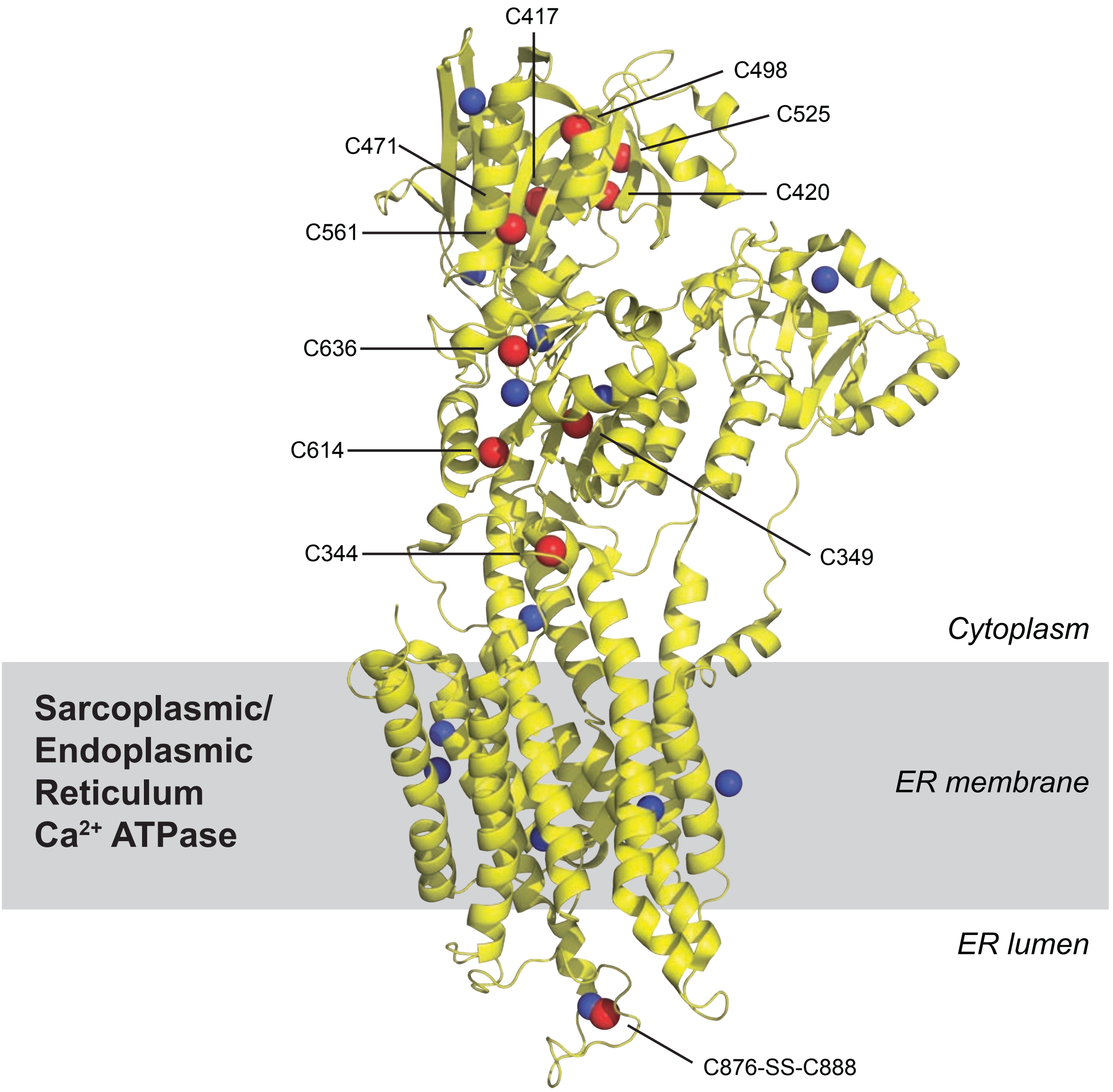

Subunit (Uniprot)	Cys residue	Control (% oxidation) Untreated	Control H_2O_2	Control PQ	Control Fasting	Catalase (% oxidation) Untreated	Catalase H_2O_2	Catalase PQ	Catalase Fasting
(P22700)	344 349	27.8 (4)	19.7 (5)	23.0 (5)	41.0 (5)	23.0 (5)	23.9 (5)	31.1 (5)	32.2 (5)
(P22700)	417 420	30.8 (4)	32.5 (4)	24.4 (5)	38.2 (4)	35.9 (5)	34.6 (5)	32.3 (4)	31.4 (5)
(P22700)	471	6.1 (5)	6.9 (5)	14.4 (5)	26.7 (5)	10.6 (5)	9.1 (5)	17.0 (5)	22.8 (5)
(P22700)	498	18.2 (5)	13.7 (5)	13.7 (5)	27.0 (5)	17.7 (5)	13.8 (5)	17.5 (5)	23.9 (5)
(P22700)	525	6.3 (4)	8.5 (4)			12.1 (3)	10.3 (3)	20.4 (1)	18.1 (1)
(P22700)	561	17.8 (2)	20.5 (4)	17.0 (5)	31.8 (5)	20.2 (4)	16.7 (4)	21.7 (5)	23.3 (4)
(P22700)	614	2.7 (1)						11.3 (1)	
(P22700)	636	5.3 (5)	7.5 (5)	9.4 (5)	22.6 (5)	9.4 (4)	6.3 (5)	16.3 (5)	15.1 (5)
(P22700)	876	68.2 (1)	62.2 (1)	26.5 (4)	44.7 (5)	71.3 (2)	68.3 (1)	27.8 (4)	30.2 (4)

Figure S7. The Redox Changes Observed During Stress Occur Within the ER

Oryctolagus cuniculus sarcoplasmic/endoplasmic reticulum Ca^{2+} ATPase (SERCA) spans the ER membrane and contains a single subunit (3W5C, yellow) and is 71% homologous to *D. melanogaster*. The sulphur atom of cysteine residues on the *O. cuniculus* structure that are observed by OxICAT are shown as red spheres and numbered according to the *D. melanogaster* sequence. The sulphur atom of cysteines that are not detected by OxICAT are blue spheres. If they are present as a disulphide, their cysteine partner is also labelled. The table shows the oxidation state of each cysteine under different conditions with the number of times it was observed in parentheses.

SFig 7 is linked to Fig 5.

Figure S8

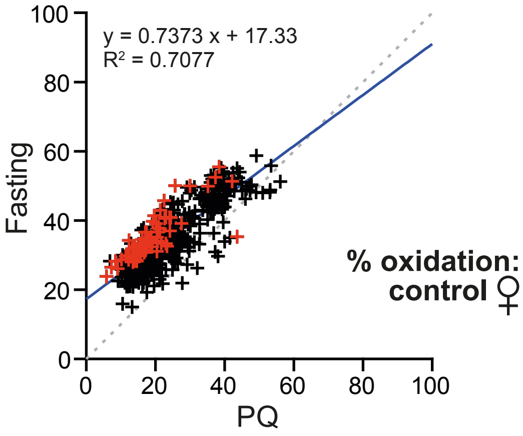

Figure S8. Comparison Between Effect of Fasting and PQ on Cysteine Residue Oxidation in Young (d 7) Control *D. melanogaster*

Oxidation state of cysteine residues present in control flies upon fasting plotted against PQ-treated flies. The dotted line has a slope of 1, while the continuous line is the least squares best-fit line to the data, with the equation for the line and Pearson's correlation coefficient indicated. The red symbols (n = 69) indicate cysteine residues with a p value smaller than 0.05 following a non-paired, two-tailed Student's t-test. No data points had a high stringency significance assessed by a Benjamini-Hochberg test. Total unique peptides = 616.

SFig 8 is linked to Fig 5.
