# Supplementary material for: Fasting, but Not Aging, Dramatically Alters the Redox Status of Cysteine Residues on Proteins in Drosophila melanogaster
Source: Cell Rep. 2015 Jun 18;11(12):1856–65. doi: 10.1016/j.celrep.2015.05.033 (PMC4508341; doi:10.1016/j.celrep.2015.05.033)
Supplement: Document S2. Article plus Supplemental Information [file mmc7.pdf]

Cell Reports

Report

Fasting, but Not Aging, Dramatically Alters the Redox Status of Cysteine Residues on Proteins in *Drosophila melanogaster*

Graphical Abstract

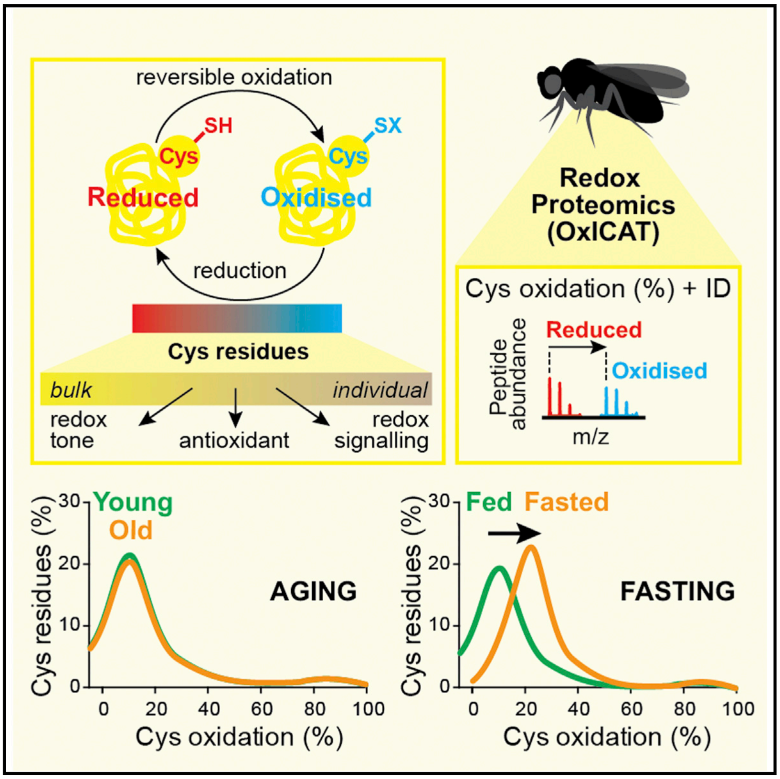

Highlights

- The redox state and identity of cysteine residues in flies can be determined by OxICAT
- Overall cysteine-residue redox state does not change with age
- H_2O_2 and paraquat have surprisingly distinct effects on cysteine-residue redox state
- Fasting for 24 hr dramatically alters the redox state of cysteine residues

Authors

Katja E. Menger, Andrew M. James, Helena M. Cochemé, ..., Ian M. Fearnley, Linda Partridge, Michael P. Murphy

Correspondence

mpm@mrc-mbu.cam.ac.uk

In Brief

Using a redox proteomic mass spectrometry technique, oxidative isotope-coded affinity tags (OxICAT), Menger et al measured cysteine residue redox changes in *Drosophila melanogaster* during aging and fasting. Surprisingly, aging had no impact on cysteine-residue redox state. In contrast, fasting dramatically affected cysteine residues, suggesting a role for cysteine-residue redox status in fasting.

Menger et al., 2015, Cell Reports *11*, 1856–1865
June 30, 2015 ©2015 The Authors
http://dx.doi.org/10.1016/j.celrep.2015.05.033

OPEN ACCESS
CellPress

Fasting, but Not Aging, Dramatically Alters the Redox Status of Cysteine Residues on Proteins in *Drosophila melanogaster*

Katja E. Menger,[1,2] Andrew M. James,[1] Helena M. Cochemé,[3,4,5] Michael E. Harbour,[1] Edward T. Chouchani,[1,6,7,8] Shujing Ding,[1] Ian M. Fearnley,[1] Linda Partridge,[3,4] and Michael P. Murphy[1,*]
[1]MRC Mitochondrial Biology Unit, Cambridge CB2 0XY, UK
[2]Institute of Ophthalmology, University College London, London EC1V 9EL, UK
[3]Institute of Healthy Ageing and GEE, University College London, London WC1E 6BT, UK
[4]Max Planck Institute for Biology of Ageing, Cologne 50931, Germany
[5]MRC Clinical Sciences Centre, Imperial College London, London W12 0NN, UK
[6]Department of Medicine, University of Cambridge, Cambridge CB2 0QQ, UK
[7]Department of Cancer Biology, Dana-Farber Cancer Institute, Boston, MA 02215, USA
[8]Department of Cell Biology, Harvard Medical School, Boston, MA 02115-5730, USA
*Correspondence: mpm@mrc-mbu.cam.ac.uk
http://dx.doi.org/10.1016/j.celrep.2015.05.033
This is an open access article under the CC BY license (http://creativecommons.org/licenses/by/4.0/).

SUMMARY

Altering the redox state of cysteine residues on protein surfaces is an important response to environmental challenges. Although aging and fasting alter many redox processes, the role of cysteine residues is uncertain. To address this, we used a redox proteomic technique, oxidative isotope-coded affinity tags (OxICAT), to assess cysteine-residue redox changes in *Drosophila melanogaster* during aging and fasting. This approach enabled us to simultaneously identify and quantify the redox state of several hundred cysteine residues in vivo. Cysteine residues within young flies had a bimodal distribution with peaks at ~10% and ~85% reversibly oxidized. Surprisingly, these cysteine residues did not become more oxidized with age. In contrast, 24 hr of fasting dramatically oxidized cysteine residues that were reduced under fed conditions while also reducing cysteine residues that were initially oxidized. We conclude that fasting, but not aging, dramatically alters cysteine-residue redox status in *D. melanogaster*.

INTRODUCTION

Organisms are continually exposed to environmental challenges that dramatically alter redox processes, changing the reduction potential of redox couples as well as the production of evanescent reactive species (Go and Jones, 2013; Murphy, 2012). These redox changes can disrupt the molecular machinery of the organism, and consequently, cells contain short-term adaptive mechanisms and a parallel capacity for activating gene expression to maintain resilience. Two important environmental challenges that involve redox changes are aging and fasting.

Aging correlates with changes in redox couples, increases in reactive species, and oxidative damage (Go and Jones, 2013; Cochemé et al., 2011), although their relationship with the mechanisms underlying aging has proven elusive. Fasting for 12–48 hr dramatically alters metabolic processes and is protective against ischemia-reperfusion injury (Robertson and Mitchell, 2013) and alters signaling pathways in flies (Le Bourg, 2013; Webster et al., 2014). Intermittent starvation can be particularly effective in improving health and extending lifespan, and it may mediate some of the effects of dietary restriction (DR) (Fontana and Partridge, 2015). In addition, it is not clear if DR slows changes that occur during aging or instead protects against their consequences for health and mortality (Fontana and Partridge, 2015). While the molecular mechanisms underlying the benefits of fasting are obscure (Robertson and Mitchell, 2013), redox alterations are likely to be central.

To explore how aging and fasting affect redox state, we used the fruit fly *Drosophila melanogaster* and focused on reversible redox alterations to exposed cysteine residues. These often lack a clear structural or catalytic role and are a major, but underappreciated, component of the integrated response of the cell to redox alterations (Go and Jones, 2013; Murphy, 2012). Cysteine residues are the most abundant cellular thiol, and in the mitochondrial matrix, the concentration is ~20- to 30-fold greater than glutathione (GSH) (Go and Jones, 2013; Requejo et al., 2010). A proportion of protein thiols are particularly reactive due to changes in pK_a, accessibility and orientation wrought by the local environment (Go and Jones, 2013; Held and Gibson, 2012). Potential modifications to cysteine residues include disulfides, S-nitrosothiols, sulfenic acids, S-acylation, and S-thiolation, all of which can be reversed by the GSH/glutaredoxin and thioredoxin (Trx) systems (Murphy, 2012; Go and Jones, 2013; Held and Gibson, 2012). These changes are part of the bulk redox tone, and small changes to a large number of different cysteine residues are likely to buffer the cellular redox environment to cope with changes in redox couples and reactive species (Go and Jones, 2013). Protein cysteine residues can also

CrossMark

prevent local damage by sequestering reactive species (Go and Jones, 2013). Finally, a proportion of protein cysteine residues will undergo reversible modifications that can alter protein activity, location, or function and thereby coordinate the transmission of redox signals (D'Autréaux and Toledano, 2007; Sobotta et al., 2015). Therefore, cysteine residues are central to the cellular response to environmental challenges through the bulk redox tone or by more specific contributions to antioxidant defenses and redox signaling (Go and Jones, 2013; Held and Gibson, 2012; Leichert et al., 2008; Murphy, 2012). Consequently, assessing shifts in redox state as well as the identities of individual cysteine residues that change will contribute to our understanding of how organisms respond to aging and fasting (Figure 1A).

Assessing protein cysteine-residue redox changes is technically demanding, due to the range and evanescent nature of reversible redox changes and to the large number of residues involved, and because specific modifications to particular residues as well as small shifts in the population are both important. To address this, the ICAT (isotope-coded affinity tags) method was adapted for redox proteomics as the oxidative isotope-coded affinity tags (OxICAT) approach (Leichert et al., 2008) (Figure 1B). This enables the redox state of a large number of cysteine residues to be determined simultaneously, as well as the identification of the individual cysteines involved. The OxICAT approach has been used to investigate reversible cysteine residue oxidation within *Escherichia coli* (Leichert et al., 2008), *Saccharomyces cerevisiae* (Brandes et al., 2011), *Caenorhabditis elegans* (Knoefler et al., 2012), rat sperm (Baker et al., 2015), and mammalian cells (Go et al., 2011). Here, we have extended the OxICAT approach to *D. melanogaster* (Figure 1B) to assess reversible redox changes to cysteine residues during aging and fasting.

RESULTS

Using OxICAT to Measure Cysteine Residue Redox State in Flies

We used cohorts of ten control female flies to reduce biological variation and rapidly froze these before separating the heads and thoraces from the abdomens (Figure 1B). We focused on non-reproductive tissues to isolate the effects of age and fasting on similar tissue types, as the female abdomen changes markedly with age. Cysteine residues were stabilized by homogenization in trichloroacetic acid (TCA) to prevent artifactual thiol oxidation and disulfide shuffling (Held and Gibson, 2012; Leichert et al., 2008), then proteins were precipitated and processed for OxICAT analysis (Figure 1B). Example chromatograms and mass spectra are shown in Figures 1C–1E. Overall, peaks were observed for ~1,191 cysteine residues on ~1,082 peptides, which were labeled by both the heavy and light ICAT reagents, corresponding to ~424 proteins (Tables S1 and S2).

In Figure 2A, the $\log_{10}$ intensity of the ion count for the peptide is plotted against the percentage oxidation of that cysteine residue. This shows there is no correlation between abundance and oxidation state that could indicate systematic bias in the methodology. We only considered cysteine residues that were labeled with both light and heavy ICAT labels in at least three biological replicates out of five. In control flies, we quantified the percentage oxidation of ~537 cysteine residues, on 491 peptides, corresponding to 214 proteins (Figure 2A; Table S1). This compares favorably with previous OxICAT analyses, which based conclusions on ~400 peptides from 290 proteins in yeast (Brandes et al., 2011), 170 peptides from 137 proteins in *C. elegans* (Knoefler et al., 2012), and 641 peptides from 333 proteins in mammalian cells (Go et al., 2011).

When the cysteine population of each replicate was grouped into 5% quantiles (Figure 2B), the level of reversible oxidation was clustered around a mode of ~10%, with a small number ~85% redox modified (Figure 2B). This was in agreement with other OxICAT studies, which found that the majority of cysteine residues are partially (~5%–25%) oxidized (Brandes et al., 2011; Go et al., 2011; Knoefler et al., 2012; Leichert et al., 2008).

Cysteine residues with low signal intensity were frequently measured as 0% and 100% oxidized because only the light or the heavy labeled peptide was detected. We have discarded these data points to minimize distortions due to peptide abundance. However, this may exclude cysteine residues that are fully reduced or oxidized in vivo. When we reassessed the data in Figure 2A to include peptides labeled by only one ICAT reagent, we expanded the number of peptides to 862 (Figure S2A). However, we only detected 6 fully reduced (red symbols; Figure S2A) and 14 fully oxidized cysteine residues (blue symbols; Figure S2A). Thus, most cysteine residues are partially oxidized, and excluding singly labeled cysteine-containing peptides does not distort our analysis.

To assess the reliability of OxICAT labeling, we treated tissue homogenates with the reducing agent tris(2-carboxyethyl)phosphine (TCEP) prior to analysis and this lowered the oxidation state of the cysteine residues (Figure S2B). Similarly, oxidation of homogenates with H_2O_2 greatly increased cysteine residue oxidation (Figure S2C). Therefore our analyses accurately reflect protein thiol redox states in fly homogenates.

The weighted arithmetic mean of the % oxidation of cysteine residues in Figure 2B is ~22%. To assess the average cysteine residue redox state by an orthogonal technique, we quantified protein thiols with DTNB, giving 144 ± 25 nmol thiol/mg protein (n = 3 ± SEM), then treated the sample with DTT, which increased the thiols detected to 195 ± 12 nmol thiol/mg protein (n = 3 ± SEM), implying a percentage protein thiol oxidation of ~26%, similar to that obtained from OxICAT (Figure 2B).

As expected, some of the highly oxidized cysteine residues identified in control flies were on extracellular proteins such as transferrin 1 and general odorant binding protein 99a (Table S1). In contrast, intracellular proteins such as heat shock protein 83 and GAPDH II had more reduced cysteine residues. To further illustrate compartmentalization of redox state, we considered the Na^+/K^+-ATPase, which has intracellular and extracellular cysteine residues (Shinoda et al., 2009) (Figure 2C). The β-subunit of the Na^+/K^+-ATPase (Q24048) has six cysteine residues in the relatively oxidized extracellular space that form three disulfides in the molecular structure (2ZXE). Five of the extracellular cysteine residues from the β_2 isoform that we observed by OxICAT were oxidized (Figure 2C; 80.4% ± 2.3%). This β_2 isoform also contains a cytoplasmic cysteine residue, and this was largely reduced (20.2%). An additional eight cysteine residues on the cytoplasmic domain of the α-subunit (E1JIR4) were

OPEN
ACCESS
CellPress

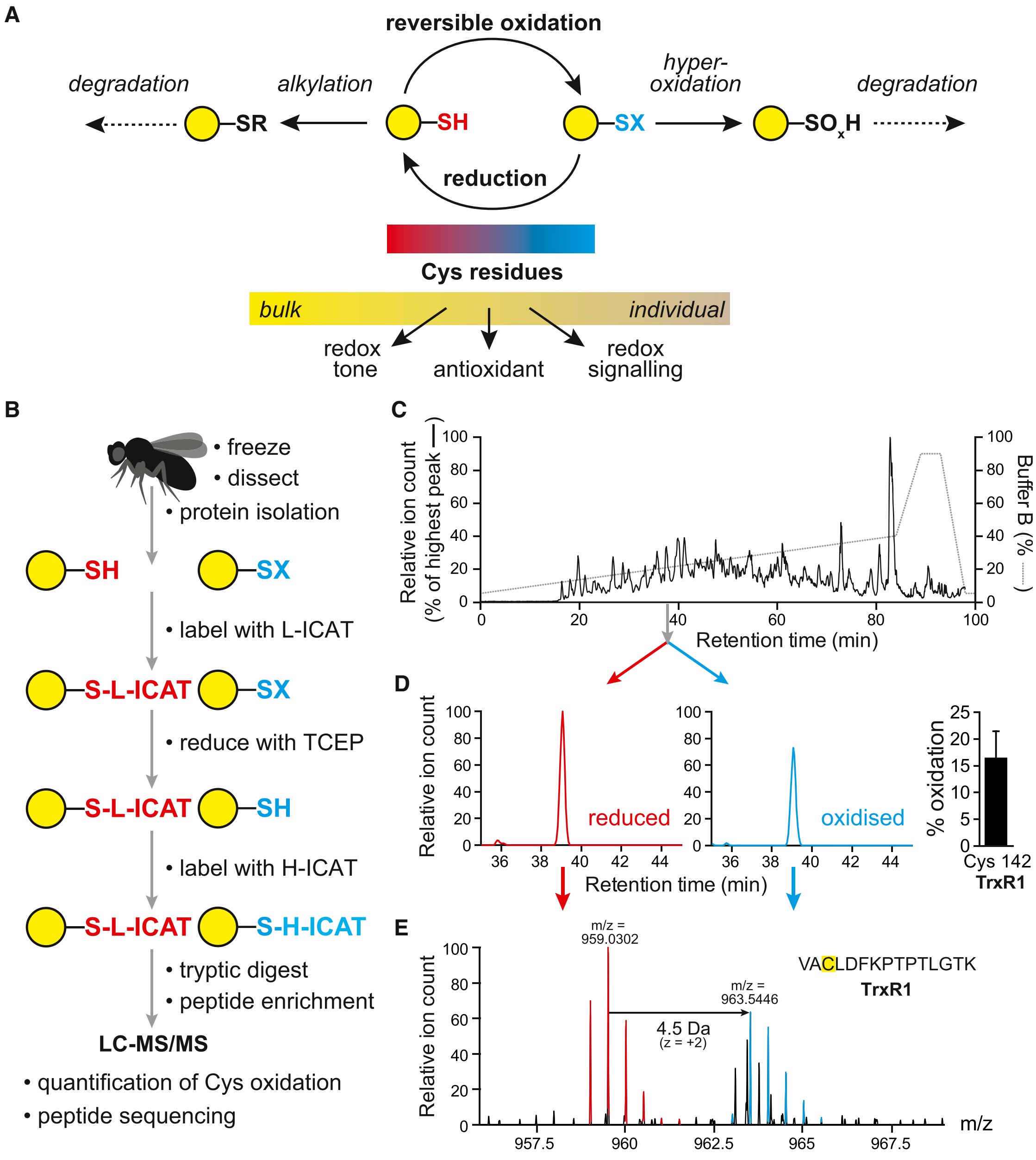

Figure 1. Assessment of Protein Cysteine-Residue Redox State in Flies

(A) Schematic showing how exposed cysteine residue can be reversibly oxidized and reduced by GSH/glutaredoxin (Grx) and Trx.

(B) OxICAT methodology. Flies are rapidly frozen, and the heads and thoraces are homogenized in 100% TCA to separate solubilized protein from the exoskeleton and then diluted to 20% TCA to precipitate proteins. The protein homogenate is then reacted with the Light ICAT reagent (L-ICAT, red) to label reduced cysteine residues (Pr-SH). After reduction of reversibly oxidized cysteine residues (Pr-SX), these thiols are reacted with the heavy ICAT reagent (H-ICAT, blue). After tryptic digestion and enrichment of labeled peptides, the biotin tags are cleaved off before separation by liquid chromatography and analysis by mass spectrometry, enabling the peptide sequence and the ratio of heavy and light labeled cysteine-containing peptides to be determined simultaneously.

(C) A typical chromatogram from control flies (UAS-cat/+). A cysteine peptide oxidized and reduced pair (retention time = 39 min) is highlighted.

(D) Chromatograms for the heavy and light labeled peptide eluting at 39 min are shown. The percentage oxidation of that cysteine residue was determined (bar chart).

(E) The peptide eluting at 39 min was identified by mass spectrometry as a component of thioredoxin reductase-1 (TrxR1). This gene encodes both a mitochondrial and a shorter cytoplasmic splice variant. The peptide could arise from either isoform but has been numbered as Cys142 from the mitochondrial isoform. See also Figure S1.

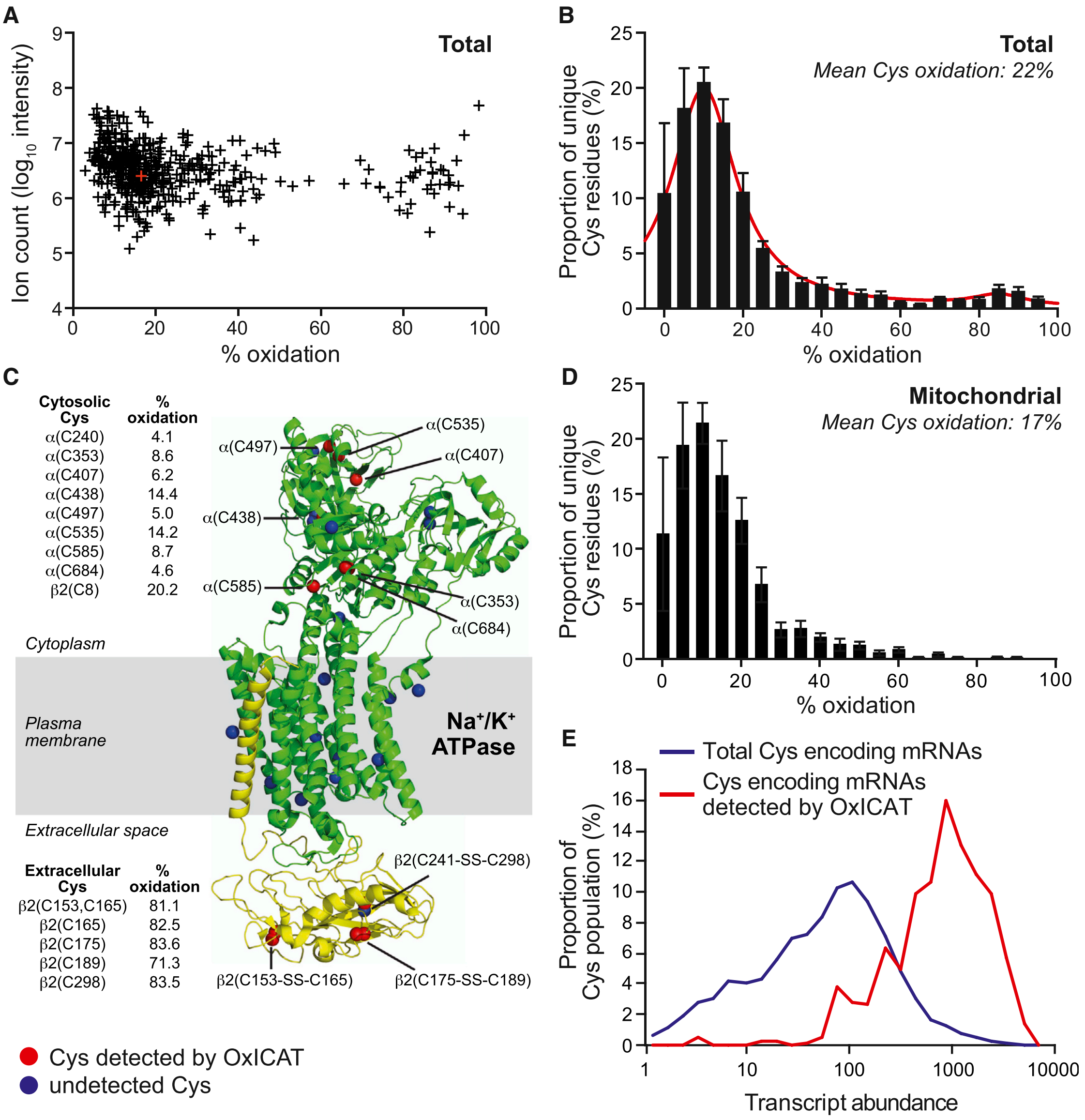

Figure 2. OxICAT Analysis of Control Young Female *D. melanogaster*

(A) Ion count for peptides plotted against percentage oxidation of the cysteine residue. The ion count is the $\log_{10}$ intensity of the sum of the heavy and light peptides. Data are the averages over three to five biological replicates. Red cross is cysteine residue 142 from TrxR1 (Figure 1C).

(B) Distribution of total cysteine residue oxidation levels. Plotted are the means of the proportion of the total number of peptides containing unique cysteine residues in each 5% quantile of percentage oxidation across five biological replicates (mean ± SEM). Total unique peptides = 491.

(C) Plasma membrane Na^+/K^+ ATPase. *D. melanogaster* Na^+/K^+ ATPase contains an α subunit and a β subunit with multiple isoforms. The monomeric structure from *S. acanthias* containing subunit α (green) and subunit β1 (yellow) is 77% and 25% homologous to the α and β2 subunits of *D. melanogaster*, which were detected by OxICAT. Cysteine residues on the *S. acanthias* structure present in homologous positions in the *D. melanogaster* α and β2 subunits are numbered. Cysteines observed by OxICAT are shown in red, and those not detected are blue. Disulfide cysteine partners are also labeled. The table shows the oxidation state of each cysteine in young control untreated flies.

(D) Oxidation state of protein cysteine residues in mitochondria. Peptides from Figure 2B that are mitochondrial are plotted as the mean of the proportion of the total number of peptides in each 5% quantile of percentage oxidation across five biological replicates (mean ± SEM). Of 214 proteins identified in Figure 2A, 87 are mitochondrial, corresponding to 214 unique cysteine residues.

(E) Comparison of peptides detected by OxICAT with transcript abundance. Whole-fly transcript intensity data were annotated to the head and thorax OxICAT dataset to characterize fly cysteines observable by mass spectrometry. Transcripts are subdivided by abundance into blocks that are $\sqrt{2}$ of the upper and lower bounds of the block immediately to the left. The percentage of both the observed OxICAT cysteine population (mean transcript abundance = 1,119; n = 849) and the total cysteine population (mean transcript abundance = 134; n = ~135,000) falling within each transcript abundance block are on the y axis.

See also Figure S2.

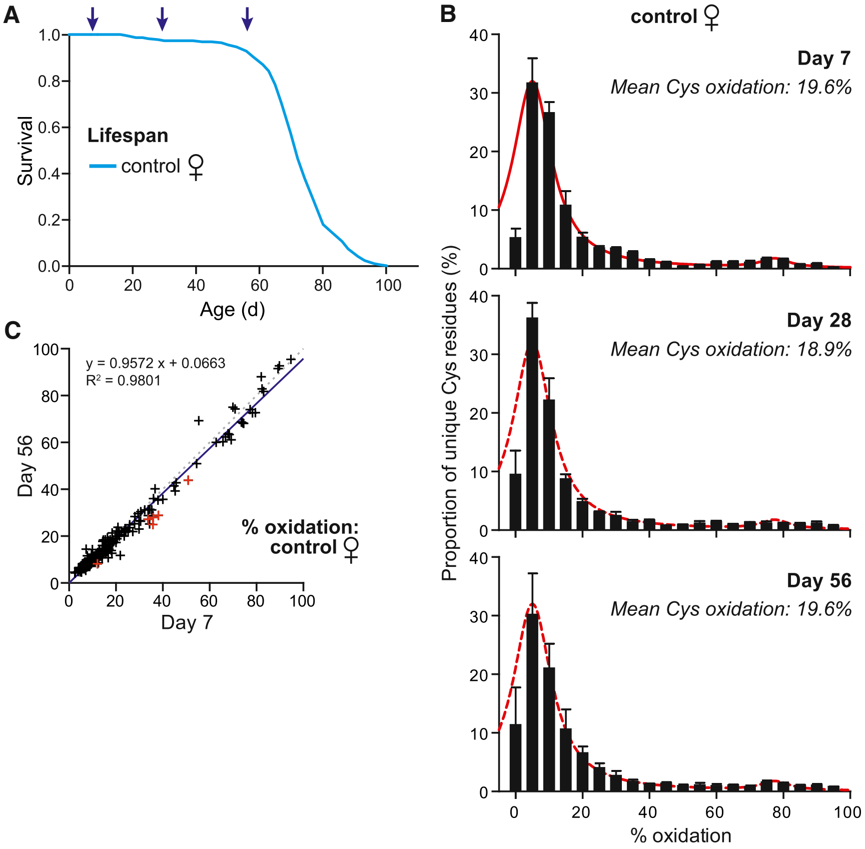

Figure 3. Reversible Oxidation Levels of Cysteine Residues in Aging *D. melanogaster*

(A) Lifespan of control female flies. Cohorts of flies were taken to analyze protein cysteine residue redox state of young (7 days), middle-aged (28 days), and old (56 days) flies.

(B) Distribution of cysteine peptides plotted against their redox states for 7-, 28-, and 56-day-old control flies. Data are means ± SEM. The red curve is for 7-day-old control flies.

(C) Oxidation state of cysteine residues present in 56-day-old flies plotted against 7-day-old flies. The dotted line slope = 1, while the continuous line is the least-squares best-fit line to the data. Data from 263 unique peptides identified at least three times under both conditions are plotted. Red symbols (n = 6) indicate low-stringency significance with $p < 0.05$ assessed by a non-paired, two-tailed Student's t test.

See also Figure S3.

largely reduced (8.2% ± 1.5%). Therefore, OxICAT reports oxidation states consistent with the known redox state and location of cysteine residues and can detect differences within a protein.

Next, we assessed cysteine residues from Figure 2B that were from the 87 proteins annotated as mitochondrial (Figure 2D). The distribution for mitochondria was similar to that for the whole fly with a mode of ~10% oxidized but with far fewer highly oxidized cysteine residues, presumably due to exclusion of extracellular and ER proteins (Figure 2B).

Our next goal was to determine what proportion of cysteine residues in transcribed proteins were represented in the OxICAT datasets. Based on an in silico digest, the total number of unique cysteines in the whole fly genome is ~135,000 on ~98,000 unique peptides. We clearly observe only a small fraction of the total; 849 tryptic peptides that contain 966 cysteine residues were observed at least once in control flies, and of these, 491 peptides were observed at least three times. Thus, we observe ~0.72% of cysteine residues in the fly genome, although many of these are not expressed in the head and thorax of the adult female fly. Furthermore, the OxICAT method will predominantly reflect the redox state of abundant proteins. To assess this, we used literature levels of mRNA transcript intensity within whole adult flies (Chintapalli et al., 2007) to assign each cysteine residue a relative abundance (Figure 2E, blue line). We then compared this with the transcript abundance of the mRNA subset encoding those peptides detected by OxICAT (Figure 2E, red line). The mean abundance for transcripts encoding cysteine residues detected by OxICAT is ~8.4-fold higher than that of the whole genome; thus, OxICAT detects redox changes in the most abundant proteins (Figure 2E). Even though we observed only ~0.72% of the potentially observable cysteine residues, these are the most abundant ones and thus contribute comparatively more to the cell redox state. If transcription reflects translation, then the cysteine residues that we assessed represent ~6% of cysteine residues by concentration within an adult fly. This is comparable with yeast studies, where OxICAT detected the oxidation state of ~5% of yeast protein thiols (Brandes et al., 2011). Even so, OxICAT should reflect the redox state of the cysteine-residue population well enough to assess biologically important questions.

No Change in Cysteine-Residue Redox State with Age

H_2O_2 is a key mediator of thiol redox state that increases with age in flies (Cochemé et al., 2011). Aging has also been correlated with an increase in oxidative damage in flies (Jacobson et al., 2010), and protein thiols become oxidized upon chronological aging in yeast (Magherini et al., 2009). We used OxICAT to quantify the effect of aging on the oxidation of cysteine residues (Figure 3A). Surprisingly, despite increases in H_2O_2 (Cochemé et al., 2011) and oxidative damage with age, the cysteine-residue oxidation state did not shift between young (7 days), middle-aged (28 days), and old (56 days) control flies, and the weighted mean percentage oxidation was also almost unaffected (Figure 3B). To see if there were shifts in the redox state of individual proteins with age that were masked by the overall trend, we plotted the redox state of individual cysteine residues detected in both the young and old control flies and again observed no change in redox state (Figure 3C). Similarly, there were no changes between 7 days and 28 days (Figure S3A) or between 28 days and 56 days (Figure S3B; Table S3).

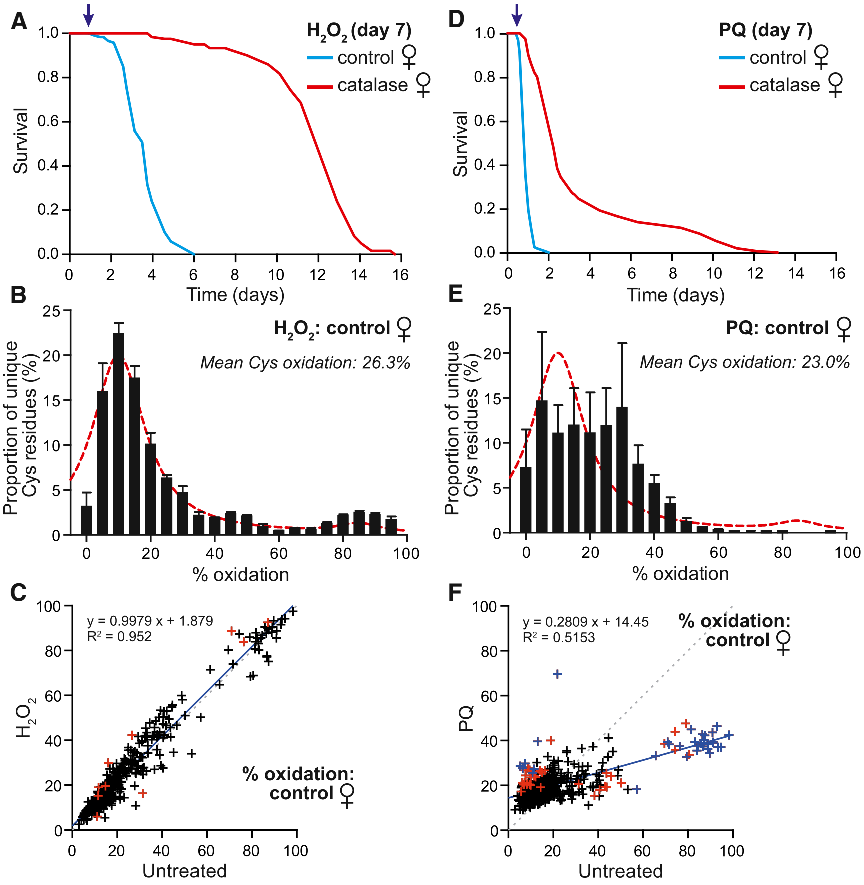

Figure 4. Effect of Exogenous Oxidants on Protein Cysteine-Residue Redox State and Fly Survival

(A) Survival of young (7 days) control and catalase-overexpressing flies after exposure to H_2O_2. Arrow indicates when cohorts are collected (24-hr treatment).

(B) Distribution of cysteine peptides plotted against redox states of the cysteine residues for control flies after exposure to H_2O_2. Data show the mean of five biological samples where each cysteine residue identified is sorted into corresponding 5% quantiles, and the resulting distributions are averaged (mean ± SEM). Dashed line indicates the untreated control (cf. Figure 2B).

(C) Oxidation state of cysteine residues present in at least three biological replicates exposed to H_2O_2 plotted against the same cysteine residues present in at least three biological replicates of controls. Dotted line slope = 1, whereas the continuous line is the best fit to the data. Red symbols (n = 12) indicate cysteine residues significantly different following a non-paired, two-tailed Student's t test ($p < 0.05$). Total unique peptides = 452.

(D) Survival of young (7 days) control and catalase overexpressing flies after exposure to PQ. Arrow indicates where cohorts are sampled (24-hr treatment).

(E) Distribution of cysteine-containing peptides plotted against redox states of the cysteine residues for control flies after exposure to PQ. Means are across five biological replicates of the relative number of cysteine residues within each 5% quantile. The dashed line is the untreated control cohort (cf. Figure 2B).

(F) Oxidation state of cysteine residues in control flies exposed to PQ plotted against untreated flies. Dotted line slope = 1, while the continuous line is the line of best fit. Each symbol represents a cysteine residue identified in at least three biological replicates of the untreated as well as the PQ-treated cohort. Red symbols identify cysteine residues (n = 68; $p < 0.05$; non-paired, two-tailed Student's t test with low-stringency significance). The blue symbols (n = 33) indicate a high-stringency significance (Benjamini-Hochberg test). Total unique peptides = 452.

See also Figure S4.

Effects of H_2O_2 and Paraquat on Cysteine-Residue Redox State

Given that a lack of cysteine-residue oxidation with age was surprising, we investigated whether cysteine-residue oxidation responded to H_2O_2 in vivo. Dietary H_2O_2 dramatically decreased survival of control flies, and overexpressing catalase conferred resistance (Figure 4A). Next, we analyzed control untreated and H_2O_2-treated flies by OxICAT, and we found there was a marginal oxidation of the cysteine residues (26.3%; Figure 4B) in comparison to untreated control flies (22%; Figure 2B). The redox state of the individual cysteine residues following H_2O_2 treatment was then plotted against those in untreated control flies (Figure 4C). If H_2O_2 treatment did not affect cysteine-residue redox state, then the points would lie on the dotted line, and H_2O_2 shifted very few peptides above this line (Figure 4C). Overexpressing catalase had little effect on cysteine residue oxidation by H_2O_2 (Figures S4A and S4B). Thus, surprisingly, H_2O_2 levels that dramatically decrease survival did so without oxidizing cysteine residues (Figure 4C).

The redox cycler paraquat (PQ) also drastically decreased fly survival, and this was partially rescued by catalase (Figure 4D). However, in contrast to H_2O_2, PQ toxicity was associated with a dramatic oxidation of cysteine residues (Figure 4E). The weighted arithmetic mean of the cysteine-residue oxidation state was not increased by PQ due to the parallel loss of highly oxidized cysteine residues (23%; Figure 4E), reinforcing the usefulness of OxICAT relative to other bulk sampling techniques. When we plotted the effect of PQ on the redox state of individual cysteine residues, many that were <20% oxidized in the untreated control became more oxidized upon PQ treatment, moving above the dotted line (Figure 4F). In contrast, PQ exposure decreased the oxidation of those cysteine residues that were >20% oxidized in the untreated control, moving them below the dotted line (Figure 4F). These contrasting effects meant that there was only a weak correlation between untreated control and PQ exposure. In addition, the oxidation of cysteine residues by PQ was attenuated by catalase overexpression (Figures S4C–S4E). Interestingly, catalase overexpression had no effect on the PQ-induced decrease in oxidation of cysteine residues. Those cysteine residues that showed significant differences (Benjamini-Hochberg test) are identified by blue crosses in Figure 4F and given in Table S4.

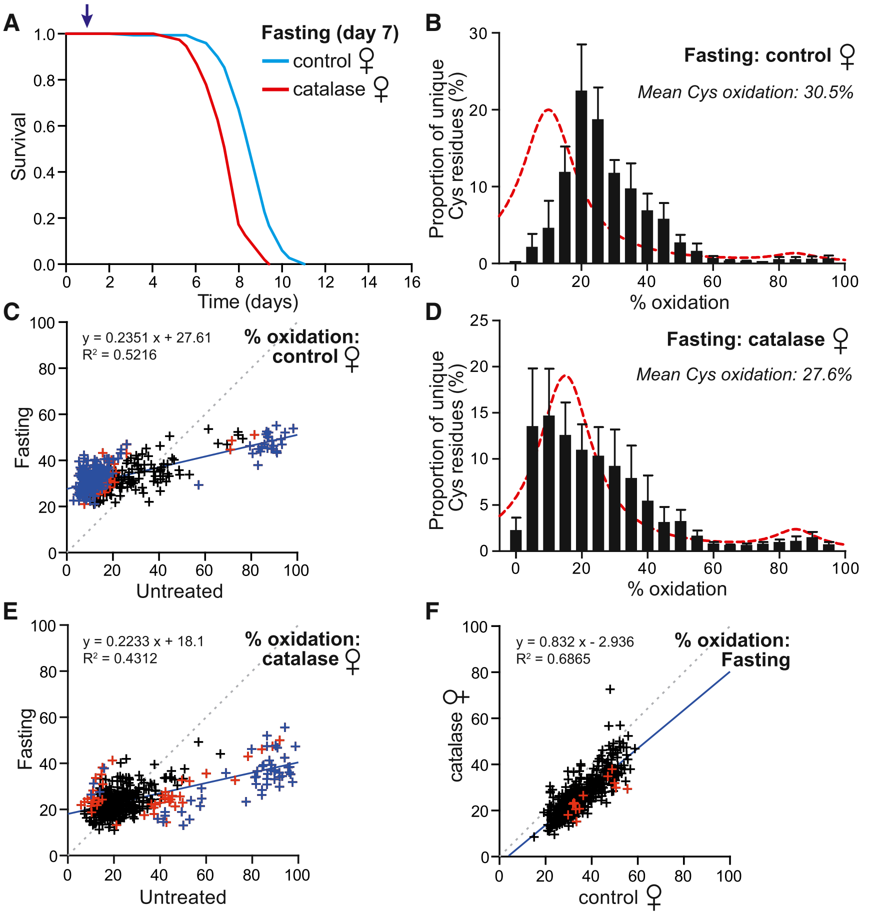

Figure 5. Effect of Fasting on Protein Cysteine-Residue Oxidation and Survival

(A) Survival of young (7 days) control and catalase-overexpressing flies during fasting. Arrow indicates where cohorts are sampled (24-hr treatment).

(B) Distribution of cysteine peptides plotted against redox states of the cysteine residues for control flies after 24-hr fasting. Shown is the mean for the relative numbers of cysteine residues in each 5% quantile of the five biological replicates. The dashed line is the curve for the untreated control (cf. Figure 2B) cohort.

(C) Oxidation state of cysteine residues present in control flies upon 24-hr fasting compared to untreated cohorts. Dotted line slope = 1, while the continuous line is the best fit to the data. Each symbol represents a cysteine residue identified in at least three biological replicates of both the control untreated as well as the fasted cohort. Red symbols identify cysteine residues (n = 252) with $p < 0.05$ (non-paired, two-tailed Student's t test), while blue symbols (n = 200) indicate a high-stringency significance (Benjamini-Hochberg test). Total unique peptides = 387.

(D) Distribution of cysteine peptides plotted against redox states of the cysteine residues for catalase-overexpressing flies after 24-hr fasting. Shown is the mean for the relative numbers of cysteine residues in each 5% quantile of the five biological replicates. Dashed line is the distribution for untreated catalase-overexpressing flies on control food.

(E) Oxidation state of cysteine residues in catalase-overexpressing flies upon 24 hr fasting against untreated cohorts. Dotted line slope = 1, while the continuous line is the best fit to the data. Each symbol represents a cysteine residue identified in at least three biological replicates of both the untreated and fasted cohorts. Red symbols identify cysteine residues (n = 96) with $p < 0.05$ (non-paired, two-tailed Student's t test). Blue symbols (n = 51) indicate high-stringency significance assessed (Benjamini-Hochberg test). Total unique peptides = 440.

(F) Oxidation state of cysteine residues present upon 24-hr fasting in catalase-overexpressing flies plotted against control flies. Dotted line slope = 1, while the continuous line is best fit to the data. Each symbol represents a cysteine residue that was identified in at least three biological replicates of both the fasted control and catalase-overexpressing flies. Red symbols identify cysteine residues (n = 13) $p < 0.05$ (non-paired, two-tailed Student's t test). Total unique peptides = 601. See also Figures S5–S8.

Effect of Fasting on Cysteine Residue Redox State

To explore the effects of fasting on cysteine residue redox state, we fasted flies for 24 hr. Because the flies survived 7–10 days of fasting (Figure 5A), any redox events within 24 hr are an early adaptive response. Starting from young (7 days) control flies, fasting led to a substantial oxidation of cysteine residues (30.5%; Figure 5B). Comparing the redox state of individual cysteines after 24 hr fasting showed that there was a dramatic difference compared to fed flies (Figure 5C). This was due to oxidation of those cysteine residues that were largely reduced in fed, untreated controls, along with the reduction of cysteine residues that were oxidized in fed, untreated controls (Table S5).

The shifts in cysteine residue redox state during fasting could reflect changes in a particular cellular compartment. To investigate this, we looked at how cysteine residues on four complexes that span three different membranes responded to fasting. The cytoplasmic cysteine residues of the plasma membrane Na^+/K^+-ATPase became more oxidized during fasting (29.3% ± 1.2% versus 9.7% ± 1.3%), while the residues facing the extracellular environment became more reduced (46% ± 3.6% versus 80.4% ± 2.3%; Figure S5). Mitochondrial matrix cysteine residues on cytochrome *bc*-1 complex and cytochrome oxidase became more oxidized during fasting (37.1% ± 3.8% versus 9.5% ± 3.1%), whereas three cysteine residues that were observed as disulfides in the protein structures became more reduced (52.7% ± 0.6% versus 93.5% ± 3.2%; Figure S6). Finally, cytoplasmic cysteine residues of the sarcoplasmic/ER Ca^{2+}-ATPase (SERCA) became more oxidized during fasting (31.2% ± 4.1% versus 17.7% ± 5.6%), while a cysteine residue that forms part of a disulfide in the ER lumen became more reduced (Figure S7). Taken together, it is clear that the redox changes observed during fasting were not confined to one compartment.

Because the cysteine-residue alterations suggested that redox changes occur during fasting, we next looked at the effect of catalase. Catalase overexpression slightly decreased survival in response to fasting compared to controls (Figure 4A). Most interestingly, catalase overexpression also reduced cysteine

residues after 24 hr fasting (Figure 4D), and the weighted arithmetic mean of the cysteine-residue redox state (27.6%) was lower than for fasted controls. By plotting the redox state of individual cysteine residues under fasted conditions against fed, it was clear that catalase protected cysteine residues from oxidation during fasting, suggesting a role for H_2O_2 (Figures 4C, 4E, and 5F). In contrast, the reduction of oxidized cysteine residues was catalase insensitive, suggesting that this is H_2O_2 independent (Figures 4C, 5E, and 5F). Interestingly, fasting altered cysteine redox state to a greater extent than PQ treatment (Figure S8), although the patterns were qualitatively similar. Overall, these data are consistent with dramatic redox changes occurring rapidly upon fasting that lead to the cysteine-residue oxidation.

DISCUSSION

We assessed how cysteine-residue redox state changes within fruit flies in two situations: aging and fasting. The former is associated with physiological decline, while fasting for short periods (~24 hr) leads to major metabolic changes. However, in both cases, the mechanisms are obscure and the role of redox changes to cysteine residues was not known. To address this, we used OxICAT to assess reversible redox changes in cysteine residues, enabling us to both assess the redox state of hundreds of cysteine residues simultaneously while also identifying the residues. To our surprise, we found that aging has no effect on cysteine-residue redox state. In stark contrast, fasting led to a dramatic reversible oxidation of protein thiols. These findings suggest that modulation of the redox state of cysteine residues is an early critical stage in the organism's response to fasting.

The OxICAT approach gives a reasonable snapshot of the redox state of cysteine residues within living flies. From this, we can infer that the majority of cysteine residues are predominantly present as the free thiol (~90% reduced), with a small number having undergone reversible oxidation so that they are ~80%–90% oxidized. Many of the cysteine residues in the oxidized population are internal protein disulfides as they are in this form in the structures investigated (Figures S5–S7) or are on secreted proteins where we expect internal disulfides. However, the OxICAT approach cannot determine the nature of the modification, and many other reversible cysteine residue modifications are possible. Furthermore, this approach will not be able to pick up irreversibly oxidized or alkylated cysteine residues (e.g., sulfinic acids or thioethers), although there was no substantial loss of intensity as would be expected with widespread irreversible modification in PQ treatment and fasting. This distribution agrees with other redox proteomic studies, but it is possible that in vivo the cysteine residues are on average more reduced and that some oxidation occurs during preparation. Even if this is the case, the validity of relative changes in redox state remains.

The investigation of the effects of two exogenous forms of oxidative stress on protein cysteine redox state by OxICAT, H_2O_2 and PQ, provided intriguing and surprising results. The first was that even toxic levels of H_2O_2 did not alter protein thiol redox state. The interaction of H_2O_2 with protein thiols is an emerging area of redox signaling. Recently, it has become clear that this signaling is quite selective, consistent with the view that H_2O_2 reacts too slowly to affect many proteins but instead modifies highly reactive proteins such as peroxiredoxins (Prx) that then relay the change by thiol-disulfide exchange with target proteins (D'Autréaux and Toledano, 2007; Sobotta et al., 2015). This suggests, surprisingly, that even large amounts of H_2O_2 may have little impact on overall protein thiols, which are maintained in a reduced state even if the organism has undergone a fatal oxidative insult. However, we cannot exclude alternative possibilities, such as that the effect of H_2O_2 on the gut has a life-shortening impact that is not reflected in the bulk redox state, or that the cysteine-residue redox state recovers but the "damage" has been done, setting in motion the life-limiting processes.

In contrast to H_2O_2, the redox cycler PQ led to extensive oxidation of initially reduced cysteine residues, while at the same time reducing a substantial number of reversibly oxidized cysteine residues. This may in part be due to appetite suppression or inhibition of food consumption by PQ (Ja et al., 2007), which may mimic the increase in cysteine residue oxidation during fasting. In any case, these results contrast with the tacit assumption that different methods of increasing oxidative damage operate through broadly similar pathways. Therefore, it is clear that these two forms of oxidative stress cannot be used interchangeably nor their effects interpreted as being on the same pathway in studies of aging or oxidative damage.

There is a large body of evidence showing a correlation between oxidative damage and aging. Cysteine redox state has not been measured during aging in a multicellular organism. Despite the lack of evidence, there has been the unstated assumption that protein thiols would become more oxidized upon aging in parallel with other markers of oxidative damage. Surprisingly, when we measured cysteine-residue redox state there was no change with age. This suggests that the changes in oxidative damage that correlate with aging are not associated with changes in cysteine residues.

Fasting was markedly different from aging, as we found a dramatic increase in protein thiol oxidation after 24 hr, although the flies were perfectly viable for several days of fasting. The many changes that occur during fasting presumably arise from effects on the pentose phosphate shunt, NADP-dependent isocitrate dehydrogenase, and the NADH/NADPH transhydrogenase that supply electrons to maintain GSH and protein thiols reduced (Webster et al., 2014). However, the cysteine redox state and survival were both affected by catalase, suggesting that there was a component of H_2O_2 signaling involved. This could occur through upregulation of autophagy as fasting is known to induce autophagy, and this process is regulated by redox pathways (Aquilano et al., 2014). Thus, the dramatic shift in the redox state of cysteine residues during fasting may be associated with the activation of autophagy that provides nutrients to prolong survival.

This work extends our understanding of redox changes in major life processes—aging and fasting—in surprising ways and shows that redox processes are more subtle and complicated than suspected. It also opens up new technical approaches to investigate these changes in flies. Our results will surprise many in the aging field who have tacitly assumed that all forms of oxidative stress increase with age. There are also

considerable implication for our understanding of the mechanistic details by which fasting and DR impact on health and lifespan. Our findings now redirect the field toward investigating the evanescent changes in protein redox state in response to diet and fasting, and future work will investigate the nature of the reversible modifications, their significance, and the cysteine residues affected. It will be particularly interesting to see whether these are associated with the dramatic and reversible shift in mortality with DR in flies (Mair et al., 2003; Robertson and Mitchell, 2013).

EXPERIMENTAL PROCEDURES

Fly Husbandry

All experiments were performed with *white Dahomey* as wild-type background. The UAS-cat (Bloomington #24621) and da-GAL4 lines were backcrossed into the *white Dahomey* background for ten generations. Control (UAS-cat/+) and catalase-overexpressing (da-GAL4 > UAS-cat) females were used for experiments. For stress experiments, flies were maintained on standard sugar-yeast-agar food (SYA) for 7 days, then transferred to PQ medium, H_2O_2 medium, or fasting medium. Flies were collected by transferring to pre-chilled microtubes and flash freezing in liquid nitrogen, then stored at -80°C. Further details are provided in the Supplemental Experimental Procedures.

Protein Isolation and OxICAT Peptide Preparation

To measure the redox state of protein cysteine residues using OxICAT, we used cohorts of ten female flies and rapidly froze them in liquid N_2 (Figure 1B) and separated the frozen heads and thoraces from the abdomens on dry ice. Protein isolation, cysteine-residue labeling, peptide preparation, and protein thiol assays are described in the Supplemental Experimental Procedures.

LC-MS/MS Analysis of Peptides

Liquid chromatography-tandem mass spectrometry (LC-MS/MS) analysis of the OxICAT-labeled peptides was carried out using an Orbitrap LTQ XL (Thermo) after chromatography on a nanoscale reverse-phase column (see Figure 1B). Each sample was run twice as a technical replicate, and five biological replicates were processed per experiment. Data analysis is described in the Supplemental Experimental Procedures.

ACCESSION NUMBERS

The mass spectrometry proteomics data have been deposited to the ProteomeXchange Consortium (Vizcaíno et al., 2014) via the PRIDE partner repository with the dataset identifier PXD002195.

SUPPLEMENTAL INFORMATION

Supplemental Information includes Supplemental Experimental Procedures, eight figures, and six tables and can be found with this article online at http://dx.doi.org/10.1016/j.celrep.2015.05.033.

AUTHOR CONTRIBUTIONS

K.E.M. performed the majority of the experiments and data analysis and drafted the manuscript with M.P.M. A.M.J. developed the procedures used and assisted with data analysis and manuscript preparation. H.M.C. carried out most of the fly work and assisted with manuscript preparation. M.E.H. helped with the analysis of the mass spectrometry data. E.T.C. helped optimize aspects of the OxICAT approach. S.D. carried out the mass spectrometric experiments under the supervision of I.M.F. L.P. and M.P.M. were the grant holders and provided leadership throughout. M.P.M. and L.P. directed the project. M.P.M. oversaw manuscript preparation.

ACKNOWLEDGMENTS

This work was supported by the Medical Research Council UK (MC-A070-5PS30) and the Biotechnology and Biological Sciences Research Council UK (BB/D020786/1). We are grateful to Alan Robinson for assistance with data analysis algorithms.

Received: March 24, 2015
Revised: April 26, 2015
Accepted: May 19, 2015
Published: June 18, 2015

REFERENCES

Aquilano, K., Baldelli, S., and Ciriolo, M.R. (2014). Glutathione: new roles in redox signaling for an old antioxidant. Front Pharmacol *5*, 196.

Baker, M.A., Weinberg, A., Hetherington, L., Villaverde, A.I., and Velkov, T. (2015). Analysis of protein thiol changes occurring during rat sperm epididymal maturation. Biol. Reprod. *92*, 11.

Brandes, N., Reichmann, D., Tienson, H., Leichert, L.I., and Jakob, U. (2011). Using quantitative redox proteomics to dissect the yeast redoxome. J. Biol. Chem. *286*, 41893–41903.

Chintapalli, V.R., Wang, J., and Dow, J.A. (2007). Using FlyAtlas to identify better *Drosophila melanogaster* models of human disease. Nat. Genet. *39*, 715–720.

Cochemé, H.M., Quin, C., McQuaker, S.J., Cabreiro, F., Logan, A., Prime, T.A., Abakumova, I., Patel, J.V., Fearnley, I.M., James, A.M., et al. (2011). Measurement of H_2O_2 within living *Drosophila* during aging using a ratiometric mass spectrometry probe targeted to the mitochondrial matrix. Cell Metab. *13*, 340–350.

D'Autréaux, B., and Toledano, M.B. (2007). ROS as signalling molecules: mechanisms that generate specificity in ROS homeostasis. Nat. Rev. Mol. Cell Biol. *8*, 813–824.

Fontana, L., and Partridge, L. (2015). Promoting health and longevity through diet: from model organisms to humans. Cell *161*, 106–118.

Go, Y.M., and Jones, D.P. (2013). Thiol/disulfide redox states in signaling and sensing. Crit. Rev. Biochem. Mol. Biol. *48*, 173–181.

Go, Y.M., Duong, D.M., Peng, J., and Jones, D.P. (2011). Protein cysteines map to functional networks according to steady-state level of oxidation. J Proteomics Bioinform *4*, 196–209.

Held, J.M., and Gibson, B.W. (2012). Regulatory control or oxidative damage? Proteomic approaches to interrogate the role of cysteine oxidation status in biological processes. Mol. Cell. Proteomics *11*, 013037.

Ja, W.W., Carvalho, G.B., Mak, E.M., de la Rosa, N.N., Fang, A.Y., Liong, J.C., Brummel, T., and Benzer, S. (2007). Prandiology of *Drosophila* and the CAFE assay. Proc. Natl. Acad. Sci. USA *104*, 8253–8256.

Jacobson, J., Lambert, A.J., Portero-Otín, M., Pamplona, R., Magwere, T., Miwa, S., Driege, Y., Brand, M.D., and Partridge, L. (2010). Biomarkers of aging in *Drosophila*. Aging Cell *9*, 466–477.

Knoefler, D., Thamsen, M., Koniczek, M., Niemuth, N.J., Diederich, A.K., and Jakob, U. (2012). Quantitative *in vivo* redox sensors uncover oxidative stress as an early event in life. Mol. Cell *47*, 767–776.

Le Bourg, É. (2013). Fasting can protect young and middle-aged *Drosophila melanogaster* flies against a severe cold stress. Biogerontology *14*, 513–529.

Leichert, L.I., Gehrke, F., Gudiseva, H.V., Blackwell, T., Ilbert, M., Walker, A.K., Strahler, J.R., Andrews, P.C., and Jakob, U. (2008). Quantifying changes in the thiol redox proteome upon oxidative stress in vivo. Proc. Natl. Acad. Sci. USA *105*, 8197–8202.

Magherini, F., Carpentieri, A., Amoresano, A., Gamberi, T., De Filippo, C., Rizzetto, L., Biagini, M., Pucci, P., and Modesti, A. (2009). Different carbon sources affect lifespan and protein redox state during *Saccharomyces cerevisiae* chronological ageing. Cell. Mol. Life Sci. *66*, 933–947.

Mair, W., Goymer, P., Pletcher, S.D., and Partridge, L. (2003). Demography of dietary restriction and death in *Drosophila*. Science *301*, 1731–1733.

Murphy, M.P. (2012). Mitochondrial thiols in antioxidant protection and redox signaling: distinct roles for glutathionylation and other thiol modifications. Antioxid. Redox Signal. *16*, 476–495.

Requejo, R., Hurd, T.R., Costa, N.J., and Murphy, M.P. (2010). Cysteine residues exposed on protein surfaces are the dominant intramitochondrial thiol and may protect against oxidative damage. FEBS J. *277*, 1465–1480.

Robertson, L.T., and Mitchell, J.R. (2013). Benefits of short-term dietary restriction in mammals. Exp. Gerontol. *48*, 1043–1048.

Shinoda, T., Ogawa, H., Cornelius, F., and Toyoshima, C. (2009). Crystal structure of the sodium-potassium pump at 2.4 A resolution. Nature *459*, 446–450.

Sobotta, M.C., Liou, W., Stöcker, S., Talwar, D., Oehler, M., Ruppert, T., Scharf, A.N., and Dick, T.P. (2015). Peroxiredoxin-2 and STAT3 form a redox relay for H_2O_2 signaling. Nat. Chem. Biol. *11*, 64–70.

Vizcaíno, J.A., Deutsch, E.W., Wang, R., Csordas, A., Reisinger, F., Ríos, D., Dianes, J.A., Sun, Z., Farrah, T., Bandeira, N., et al. (2014). ProteomeXchange provides globally coordinated proteomics data submission and dissemination. Nat. Biotechnol. *32*, 223–226.

Webster, B.R., Scott, I., Traba, J., Han, K., and Sack, M.N. (2014). Regulation of autophagy and mitophagy by nutrient availability and acetylation. Biochim. Biophys. Acta *1841*, 525–534.

Cell Reports

Supplemental Information

Fasting, but Not Aging, Dramatically Alters the Redox Status of Cysteine Residues on Proteins in *Drosophila melanogaster*

Katja E. Menger, Andrew M. James, Helena M. Cochemé, Michael Harbour, Edward T. Chouchani, Shujing Ding, Ian M. Fearnley, Linda Partridge, and Michael P. Murphy

SUPPLEMENTAL INFORMATION

SUPPLEMENTAL EXPERIMENTAL PROCEDURES

Fly Husbandry

All experiments were performed with *white Dahomey* as the wild-type background (negative for the endosymbiont *Wolbachia*), maintained in large population cages with outbreeding and overlapping generations at 25˚C on a 12 h light/12 h dark cycle. The UAS-cat (Bloomington #24621) and da-GAL4 (Wodarz et al., 1995) lines were back-crossed into the *white Dahomey* background for 10 generations. Flies were raised on standard sugar-yeast-agar medium (SYA) consisting of: 5% (w/v) sucrose (granulated sugar, Tate & Lyle), 10% (w/v) autolysed yeast (903312, MP Biomedicals), 1.5% (w/v) agar (Sigma), supplemented with 3% (v/v) nipagin and 0.3% (v/v) propionic acid as mould inhibitors, added once the food had cooled down to 60˚C (Grandison et al., 2009). Control (UAS-cat/+) and catalase over-expressing (da-GAL4 > UAS-cat) female flies were used for experiments. The control UAS-cat/+ flies have wild-type levels of catalase (mRNA and protein) and are equivalent to *white Dahomey*, whereas the ubiquitous catalase over-expressors showed ~6-fold up-regulation of catalase levels (parallel manuscript in preparation). All flies were reared at standard larval density in 200 ml bottles and eclosing adults were collected over a 12 h period. Flies were mated for 48 h before sorting into females, maintained at a density of 15 per vial and transferred to new food every 2-3 d. All experiments were incubated at 25˚C, 65% humidity on a 12 h light/12 h dark cycle.

For stress experiments, flies were maintained on standard SYA for 7 d, then transferred to paraquat (PQ) medium (20 mM PQ in SYA), H_2O_2 medium (5% H_2O_2 in 5% (w/v) sugar, 1.5% (w/v) agar), or fasting medium (1.5% (w/v) agar). Survival was scored by regularly counting the number of dead flies in each vial. In parallel, live flies were sampled after 24 h stress exposure. Flies were collected by transferring to pre-chilled microtubes and flash freezing in liquid nitrogen, and then stored at -80˚C until the time of experiment.

Protein Isolation and OxICAT Peptide Preparation

To measure the redox state of protein thiols within flies using OxICAT, we used cohorts of ten female flies, to allow for biological variation, and rapidly froze them in liquid N_2 (Figure 1B). We then separated the frozen heads and thoraces from the abdomens on dry ice (Cocheme et al., 2012) to avoid variation in egg production with age. Protein isolation is usually achieved by addition of 10-20% trichloroacetic acid (TCA) to prevent artifactual thiol oxidation and disulphide shuffling, while also precipitating proteins (Held and Gibson, 2012; Leichert et al., 2008; Zander et al., 1998). However, this was not feasible in flies due to the difficulty of separating the chitin exoskeleton from the precipitated protein. To overcome this, we homogenised frozen fly heads and thoraces in 100% (w/v) TCA to stabilise thiols and solubilise proteins (Rajalingam et al., 2009), which can then be separated from the insoluble exoskeleton. Heads and thoraces of ten flies in replicates of 5 were homogenised with 10 x 5 s pulses using a pestle to fit the microtubes in conjunction with a cordless pestle motor (VWR) in 200 μl 100% (w/v) ice-cold TCA. The homogenate was incubated on ice for 5 min, and chitin and other insoluble components were pelleted at 16000 g for 5 min. A tenth of the supernatant, equivalent to the protein present in the head and thorax of one fly, was transferred to a fresh microtube and the TCA concentration was reduced to 20% (w/v) by addition of H_2O to initiate protein precipitation. The homogenate was incubated on ice for 30 min and then pelleted for 30 min at 16000 g at 4˚C. This protein precipitation was achieved with minimal protein losses or distortion of the protein complement (Figure S1A). The pellet was washed with 10% and 5% (w/v) TCA and then resuspended in 80 μl denaturing alkylating buffer (DAB; 6 M urea, 2% (w/v) SDS, 200 mM Tris-HCl, 10 mM EDTA, 100 μM DTPA, 10 μM neocuprine). The contents of one vial of light ICAT reagent (AB SCIEX, USA 4339036) in 20 μl acetonitrile (ACN) was added to label reduced cysteine residues at 37˚C and 1400 rpm on an Eppendorf Thermomixer for 2 h. Proteins were then precipitated with 5 vols. of ice-cold acetone, incubated at -20˚C for 2 h, and pelleted at 4˚C and 16000 g for 30 min. The amount of protein to be processed for OxICAT analysis (~30-40 μg protein) was optimised to ensure saturation of thiol labelling by the light ICAT reagent (Figure S1B). The pellet was washed twice with 90%

ice-cold acetone and then solubilised in 80 µl DAB. The reducing agent *tris*(2-carboxyethyl)phosphine (TCEP) (1 mM, final concentration, 2 µl of a 50 mM stock, provided with the ICAT kit) was added, reducing previously reversibly oxidised cysteine residues, which were then labelled with one vial of heavy ICAT reagent (AB SCIEX, USA 4339036) dissolved in 20 µl ACN. Proteins were incubated at 37˚C and 1400 rpm on an Eppendorf Thermomixer for 2 h, and then precipitated with 5 vol ice-cold acetone, stored at -20˚C for 2 h and pelleted at 4˚C at 16000g for 30 min. The pellet was washed twice with 90% ice-cold acetone and then resuspended in denaturing buffer and 1 vol trypsin (resuspended in H_2O) was added (both provided by the ICAT kit, AB SCIEX, USA 4339036). Proteins were digested at 37˚C and 1400 rpm on an Eppendorf Thermomixer overnight. Digested peptides were enriched for cysteine-containing peptides first on a cation exchange cartridge and then subsequently on an avidin affinity cartridge (both provided with the ICAT kit, AB SCIEX, USA 4339036). Briefly, trypsinolysis is terminated by addition of 4 ml cation exchange buffer-*load* (10 mM KH_2PO_4, 25% ACN, pH 3.0) and the sample loaded onto the pre-equilibrated cation exchange cartridge. The flow-through was retained for potential trouble-shooting and the cartridge washed with 1 ml cation exchange buffer-*load*, with the flow through collected into the same tube as the loading flow through. The sample was eluted using 500 µl of cation exchange buffer-*elute* (10 mM KH_2PO_4, 25% ACN, 350 mM KCl, pH 3.0). The pH of the eluted sample was adjusted through the addition of 500 µl affinity buffer-*load* (2x PBS, pH 7.2 [1x PBS = 10 mM NaH_2PO_4, 150 mM NaCl]) and loaded onto a pre-equlibrated affinity cartridge. The flow through was collected for potential trouble-shooting and the cartridge washed with an additional 500 µl affinity buffer-*load* combined with the initial flow-through. To remove additional salts 1ml affinity buffer-*wash I* (1x PBS, pH 7.2) was added slowly and the flow-through discarded. Unlabelled peptides were eluted from the cartridge with 1 ml affinity buffer-*wash II* (50 mM ammonium bicarbonate [NH_4HCO_3]/ 20% methanol, pH 8.3) and the flow-through collected. ICAT labelled peptides are eluted after washing the cartridge with 1 ml H_2O using 800 µl affinity buffer-*elute*, (30% (v/v) ACN, 0.4% (w/v) trifluoroacetic acid (TFA), with the first 50 µl eluate being discarded. The eluted peptides were dried down overnight in a Speed vac and the biotin moiety of the ICAT label was removed by

incubation with the cleaving reagents provided in the ICAT kit (2 h at 37˚C and 1400 rpm on an Eppendorf Thermomixer). The isolated peptides were then dried down in a speed vac.

Mass Spectrometric Data Analysis

Raw files for each LC-MS/MS run were analysed using MaxQuant software to determine the ratio of heavy over light OxICAT-labelled peptides in our experiments (Cox and Mann, 2008). Raw files from the two technical replicates obtained during two LC-MS/MS runs of the same biological replicate were grouped to provide a dataset for that biological sample. As possible modifications, besides the labelling of cysteine residues with light or heavy ICAT label, only methionine oxidation was included and two possible missed cleavages were allowed in the MaxQuant search parameters (listed in Table S6). As a reference sequence database, a FASTA file containing all protein sequences associated with *D. melanogaster* was downloaded from www.uniprot.org. (July 2011). This reference sequence database was used by the search engine Andromeda associated with MaxQuant to identify peptides detected during the LC-MS/MS run. In addition, the reference sequence database was used to create a list of all cysteine-containing tryptic peptides within the *D. melanogaster* proteome. To do this, an *in silico* tryptic digest of the complete proteome was performed using a modified proteogest script (Cagney et al., 2003), generating a file that listed all cysteine-containing tryptic peptides, with none, one or two missed cleavages, the cysteine residue number and the Uniprot accession number of the protein (Cagney et al., 2003). The proteogest script modification was done by Dr Alan Robinson, MRC Mitochondrial Biology Unit. Ratios of heavy over light ICAT labelled peptides listed in the evidence file obtained from MaxQuant were converted to % of the cysteine residue that had been reversibly oxidised. Using the SQL database software pgAdmin3, the peptide list generated by proteogest was used together with the modified evidence file to combine intensity from all peptide signals containing the cysteine residue of interest (i.e. miscleaved, methionine oxidation, different z values). This generated a mean % reversible oxidation for each unique cysteine residue identified within the biological replicates. We used the $\log_{10}$ intensity to generate averages of the abundance of the peptides with unique cysteine residues across 3 to 5 biological

replicates and across all amino acid sequences associated with the cysteine residue identified. The threshold of $\log_{10}$ intensity ~5-6 for peptide pair identification is due to the limit for detection by the mass spectrometer and is variable because the detection of individual peptides depends on sequence, abundance and co-eluting peptides.

The % reversible oxidation and the summed eXtracted Ion Current (XIC: defined as the summed eXtracted Ion Current of all isotopic clusters associated with the identified peptide sequence, averaged across the biological replicates, also used for the $\log_{10}$ intensity) remained associated during information processing. Information on the subcellular localisation of the proteins identified and the biological processes they participated in was obtained using MitoMiner (http://mitominer.mrc-mbu.cam.ac.uk/) (Smith and Robinson, 2009).

For bar charts, the data for distribution of proportion of cysteine residues against % oxidation were generated by counting the number of cysteine residues in each 5% quantile for each individual biological replicate and then averaging the relative values for each quantile over the 5 biological replicates. The fitted red curves are the data from related experiments fitted manually using a Lorentizian equation to the distribution that is re-plotted to facilitate assessment of changes.

For plots of the % oxidation of a given cysteine under one condition against its % oxidation under another condition, those peptides for which there was a shift in % oxidation assessed by an uncorrected Student's t test were plotted in red. While many of these differences will be real because of the large dataset there will also be a significant number of false positives. Therefore this gives a low-stringency visual indication of shifts in % oxidation between conditions as well as to allow other studies to definitively corroborate them by orthogonal techniques. To assess those shifts in % oxidation that can be more strongly interpreted as a shift for that cysteine residue solely from our data, we used the Benjamini-Hochberg procedure (Benjamini and Hochberg, 1995; Hochberg and Benjamini, 1990) to correct for multiple comparisons and these data are shown in blue. It has to be noted that every cysteine residue that is found to change significantly according to the Benjamini-Hochberg procedure will also have passed the level of significance in the Student's t-test.

The program pgAdmin3 was used to link existing transcriptional abundance datasets (Chintapalli et al., 2007) with the list of cysteines observed by OxICAT. Briefly, Uniprot tags (Affymetrix; Drosophila_2.na33.annot.csv) were annotated to whole fly microarray data (FlyAtlas; 20090519.txt) using probe identifications common between the two datasets. Common Uniprot tags and sequence data were then used to annotate the transcriptional information in this dataset to a theoretical trypsin digest of the whole fly proteome as well as the subset of cysteines we observed by OxICAT. This provided expression levels for each cysteine observed by OxICAT that could be compared to that of the complete fly proteome.

Protein Thiol Assays

To measure bulk protein thiols, the heads and thoraces from 20 flies were homogenised using a pestle fitted to a microtube and a cordless pestle motor (VWR) in 100 µl 100% (w/v) ice-cold TCA. The homogenate was incubated on ice for 5 min, and then chitin and other insoluble components were pelleted at 16000 g and 4˚C for 5 min. The supernatant was transferred to a fresh tube and the TCA concentration was reduced to 20% (w/v) through the addition of H_2O and the sample was incubated on ice for 30 min. The protein precipitate was pelleted by centrifugation at 16000 g and 4˚C for 30 min. The supernatant was removed and the pellet washed with 500 µl 10 % and 5 % (w/v) TCA. The pellet was resuspended in 250 µl sample buffer (20 mM Tris, 10 mM EDTA, pH 7.3 with 1 % (w/v) n-dodecyl-maltoside (DDM)), incubated on ice for 30 min and the sample was split into aliquots of 75 µl and 150 µl. The 150 µl aliquot was reduced by treating with 1 mM dithiothreitol (DTT), then split further into two 75 µl aliquots, and SDS (final concentration 2% (w/v)) was added to one of them. All aliquots were then incubated at RT for 10 min. To remove small thiol-containing molecules all aliquots were passed twice through MicroBioSpin6 columns (6 kDa; BioRad), pre-equilibrated with sample buffer. Then 10 µl of the sample or GSH standards were mixed with 160 µl assay buffer (80 mM NaH_2PO_4, 1 mM EDTA, pH 8) ± 200 µM 5,5'-dithiobis-(2-nitrobenzoic acid) (DTNB). The samples were incubated at RT for 30 min and difference in absorbance between the ± DTNB samples and standards was measured at λ_{412}. The thiol concentration of the protein samples was determined from the GSH standard curve. All

measurements were done in triplicate. In parallel the protein concentration of each aliquot was determined using the BCA assay. This allows the calculation of thiols per mg protein for either the reduced exposed protein thiols (not treated with DTT or SDS), the total exposed thiols (treated with DTT but not with SDS) or the total thiols (treated with DTT and SDS) (Requejo et al., 2010).

SUPPLEMENTAL REFERENCES

Benjamini, Y., and Hochberg, Y. (1995). Controlling the false discovery rate: a practical and powerful approach to multiple testing. J. Royal Stat. Soc., Series B *57*, 289-300.

Cagney, G., Amiri, S., Premawaradena, T., Lindo, M., and Emili, A. (2003). *In silico* proteome analysis to facilitate proteomics experiments using mass spectrometry. Proteome Sci. *1*, 5.

Chintapalli, V.R., Wang, J., and Dow, J.A. (2007). Using FlyAtlas to identify better *Drosophila melanogaster* models of human disease. Nat. Genet. *39*, 715-720.

Cochemé, H.M., Logan, A., Prime, T.A., Abakumova, I., Quin, C., McQuaker, S.J., Patel, J.V., Fearnley, I.M., James, A.M., Porteous, C.M., *et al.* (2012). Using the mitochondria-targeted ratiometric mass spectrometry probe MitoB to measure H_2O_2 in living *Drosophila*. Nat. Prot. *7*, 946-958.

Cox, J., and Mann, M. (2008). MaxQuant enables high peptide identification rates, individualized p.p.b.-range mass accuracies and proteome-wide protein quantification. Nat. Biotech. *26*, 1367-1372.

Grandison, R.C., Wong, R., Bass, T.M., Partridge, L., and Piper, M.D. (2009). Effect of a standardised dietary restriction protocol on multiple laboratory strains of *Drosophila melanogaster*. PLoS One *4*, e4067.

Held, J.M., and Gibson, B.W. (2012). Regulatory control or oxidative damage? Proteomic approaches to interrogate the role of cysteine oxidation status in biological processes. Mol. Cell. Proteom. *11*, R111 013037.

Hochberg, Y., and Benjamini, Y. (1990). More powerful procedures for multiple significance testing. Stat. Med*.* *9*, 811-818.

Leichert, L.I., Gehrke, F., Gudiseva, H.V., Blackwell, T., Ilbert, M., Walker, A.K., Strahler, J.R., Andrews, P.C., and Jakob, U. (2008). Quantifying changes in the thiol redox proteome upon oxidative stress *in vivo*. Proc. Natl. Acad. Sci. USA *105*, 8197-8202.

Rajalingam, D., Loftis, C., Xu, J.J., and Kumar, T.K. (2009). Trichloroacetic acid-induced protein precipitation involves the reversible association of a stable partially structured intermediate. Protein Sci. *18*, 980-993.

Requejo, R., Hurd, T.R., Costa, N.J., and Murphy, M.P. (2010). Cysteine residues exposed on protein surfaces are the dominant intramitochondrial thiol and may protect against oxidative damage. FEBS J. *277*, 1465-1480.

Smith, A.C., and Robinson, A.J. (2009). MitoMiner: an integrated database for the storage and analysis of mitochondrial proteomics data Mol. Cell Proteom.

Wodarz, A., Hinz, U., Engelbert, M., and Knust, E. (1995). Expression of crumbs confers apical character on plasma membrane domains of ectodermal epithelia of *Drosophila*. Cell *82*, 67-76.

Zander, T., Phadke, N.D., and Bardwell, J.C. (1998). Disulfide bond catalysts in *Escherichia coli*. Meth. Enzymol. *290*, 59-74.

Figure S1

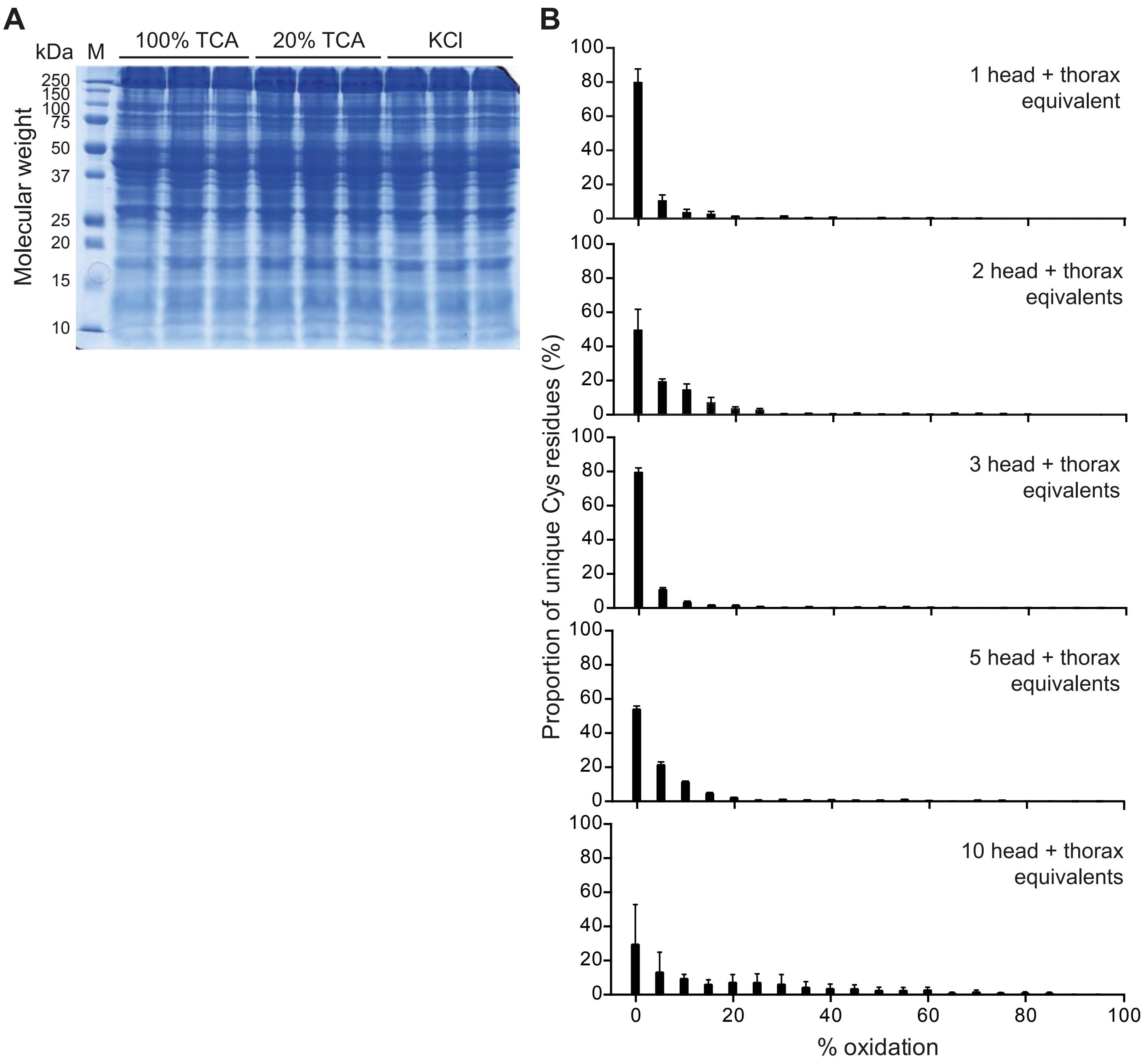

A
kDa
M
100% TCA
20% TCA
KCl
Molecular weight
250
150
100
75
50
37
25
20
15
10
B
1 head + thorax equivalent
2 head + thorax eqivalents
3 head + thorax eqivalents
5 head + thorax equivalents
10 head + thorax equivalents
Proportion of unique Cys residues (%)
% oxidation
0
20
40
60
80
100

Figure S1. Assessment of Protein Yield During OxICAT Experiments

(A) Determination of protein yield upon TCA precipitation of fly homogenates. Cohorts of young (7 d) female control flies were split into nine groups of 10 heads and thoraces. Samples were then homogenised in triplicate in either 100% (w/v) TCA, 20% (w/v) TCA, or KCl buffer (120 mM KCl, 3 mM Hepes-KOH, 1 mM EGTA, pH 7.5). The 100% TCA sample was incubated on ice for 5 min and insoluble components were pelleted by centrifugation at 16000 g, 4ºC for 5 min. The supernatant was transferred to a fresh tube and the TCA concentration lowered to 20% through the addition of H_2O. Insoluble components in the KCl buffer were pelleted by centrifugation at 16000 g, 4ºC for 5 min, and the supernatant transferred to a fresh tube where 100% TCA was added to result in a final concentration of 20% TCA. Homogenisation of the sample in 20% TCA caused protein precipitation straight away so the pellet was dissolved in loading buffer but chitin was largely retained in the bottom. All samples were incubated on ice for 30 min and the protein pelleted by centrifugation at 16000 g, 4ºC for 30 min. The pellets were washed with 500 µl 10% TCA followed by a 5% TCA wash. Proteins were resuspended in 100 µl SDS loading buffer without bromophenol blue and the protein concentration determined with the BCA assay. The protein concentration in 100% TCA, 20% TCA and KCl was 2.37 ± 0.15 mg/ml, 3.02 ± 0.04 mg/ml and 2.82 ± 0.04 mg/ml, respectively (n = 3). The 100% TCA showed a 16-21% lower amount of protein than the other methods, probably due to difficulty of eliminating keratin carryover in the 20% TCA and KCl experiments. After bromophenol was added equal amounts of 40 µg protein in 20 µl were loaded in triplicate onto a pre-cast 12% gel. The gel was run at 110 V at RT until the dye front reached the bottom. The gel was stained with coomassie at RT for 30 min and then destained, showing very similar patterns of protein distribution.

(B) Optimisation of the number of flies needed. In order to determine how efficient the ICAT labelling is with different amounts of protein a preliminary labelling with different levels of fly protein was performed. For this 10 heads and thoraces were homogenised in 100% (w/v) TCA and incubated on ice for 5 min. Insoluble components like chitin were pelleted by centrifugation and the equivalent of (top to bottom) one, two, three, five, or ten heads and thoraces was taken from the supernatant and transferred to a fresh tube with the appropriate amount of H_2O to lower the TCA concentration to 20% (w/v) TCA. The protein was pelleted and then solubilised in denaturing buffer with the reducing agent TCEP present. This reduces all previously oxidised cysteine residues. Full labelling should therefore be achieved where the majority of thiols are labelled with light ICAT reagent. After reaction with the light ICAT label, proteins were precipitated with acetone and subsequently solubilised in denaturing buffer with reducing agent TCEP and heavy ICAT label present to label all non-labelled cysteine residues. To remove excess label the proteins were precipitated by acetone and then digested by trypsin. Enrichment of the ICAT labelled peptides with an avidin column was followed by LC-MS/MS analysis. The fact that the labelling with light ICAT reagent decreases with increasing number of flies indicates that there is not enough light ICAT reagent to saturate all available thiols and therefore the protein equivalent of one fly head and thorax was used but maintaining the praxis of homogenising ten heads and thoraxes to preserve biological variability.

SFig 1 is linked to Experimental procedures.

Figure S2

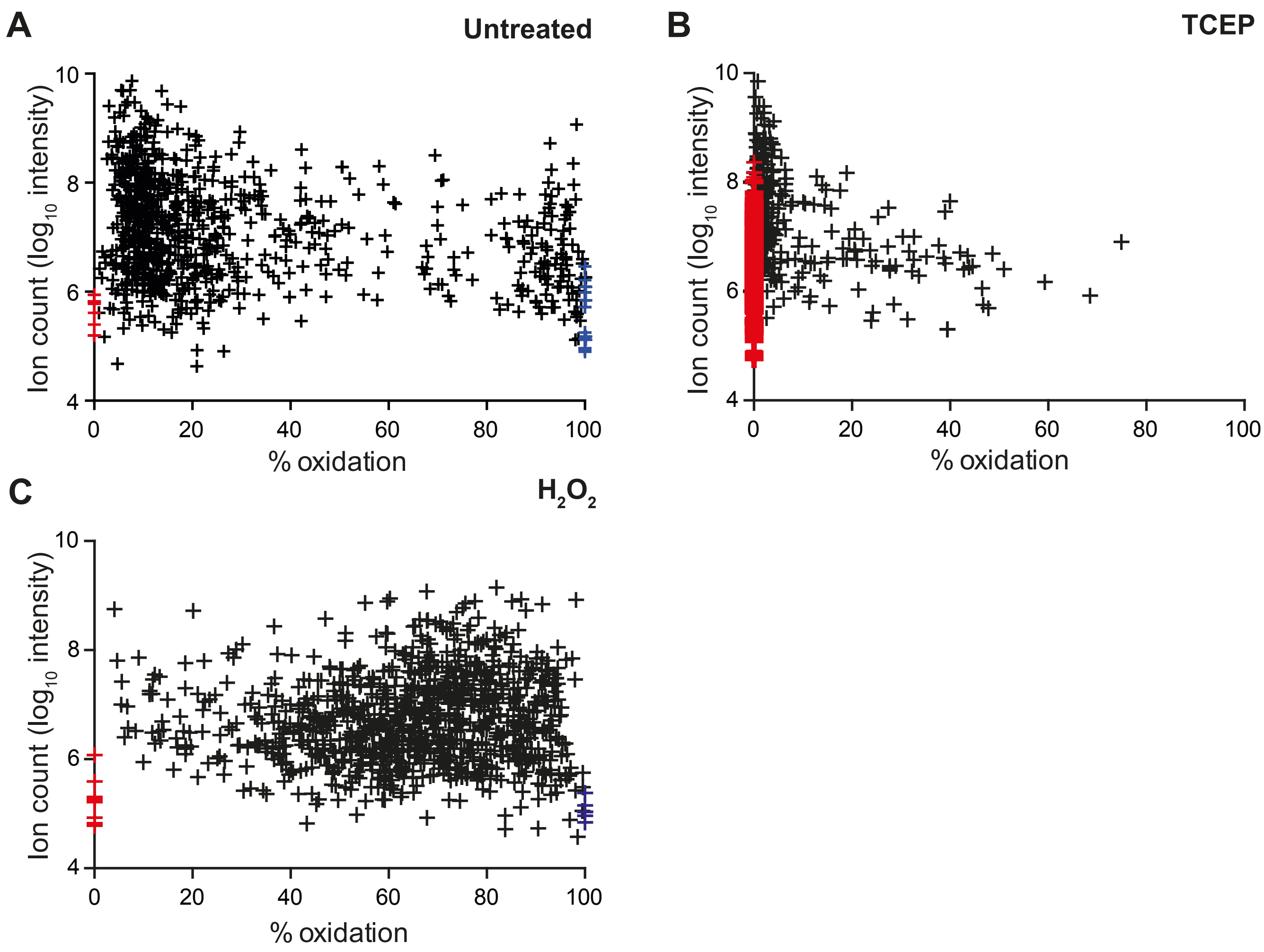

Figure S2. OxICAT Analysis of Young (7 d) Female Control *D. melanogaster*

(A) The data from Fig 2A are re-plotted to include peptides for which only the heavy labelled peptide (blue = 100% oxidised; n = 14) or light labelled peptide (red = 100% reduced; n = 6) was observed. For this the ion count for particular peptides is plotted against the % oxidation of the cysteine residue. Total unique peptides = 862.

(B) The fly homogenate prepared in the same way as in Figure 2A was treated with 1 mM TCEP in the presence of light ICAT regent (for 2 h at 37ºC) to reduce all reversible thiols, and then processed for OxICAT as usual. Red = 100% reduced; n = 434. Total unique peptides = 748.

(C) The fly homogenate prepared in the same way as in Fig 2A was treated with 5 mM H_2O_2 for 10 min to increase the oxidation of protein thiols, and then processed for OxICAT as usual. Blue = 100% oxidised; n = 6. Red = 100% reduced; n = 12. Total unique peptides = 1016.

SFig 2 is linked to Fig 2.

Figure S3

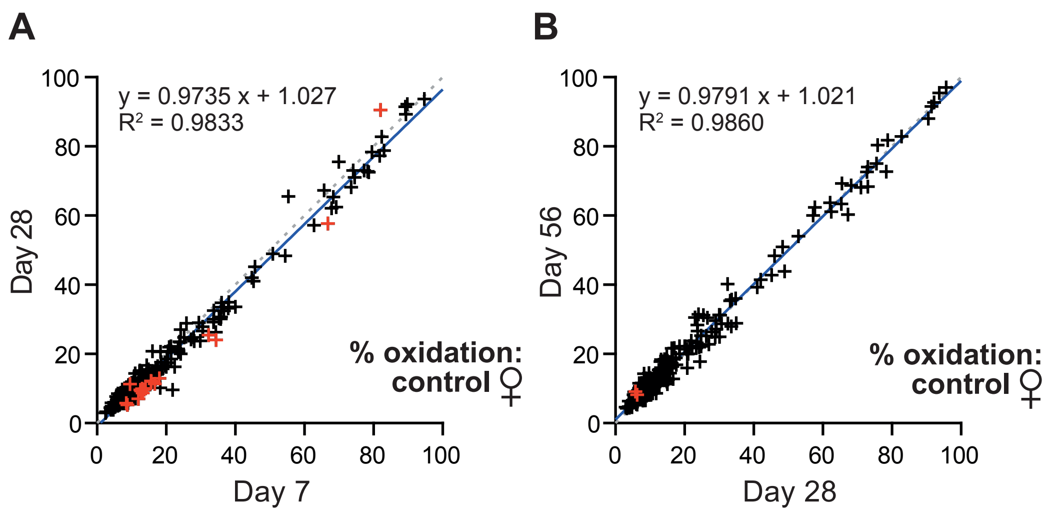

Figure S3. No Change in Protein Cysteine Residue Redox State with Age in Control *D. melanogaster*

(A) Oxidation state of cysteine residues present in control middle aged (28 d) flies plotted against young (7 d) flies. The dotted line has a slope of 1, while the continuous line is the least squares best-fit line to the data, with the equation for the line and Pearson's correlation coefficient indicated. Red symbols (n = 18) indicate a low stringency significance with a p-value smaller than 0.05 assessed by a two-tailed, non-paired Student's t-test. No data points had a high stringency significance assessed by a Benjamini-Hochberg test. Total unique peptides = 268.

(B) Oxidation state of cysteine residues present in control old (56 d) flies plotted against middle aged (28 d) flies. The dotted line has a slope of 1, while the continuous line is the least squares best-fit line to the data, with the equation for the line and Pearson's correlation coefficient indicated. Red symbols (n = 2) indicate a low stringency significance with a p-value smaller than 0.05 assessed by a two-tailed, non-paired Student's t-test. No data points had a high stringency significance assessed by a Benjamini-Hochberg test. Total unique peptides = 276.

SFig 3 is linked to Fig 3.

Figure S4

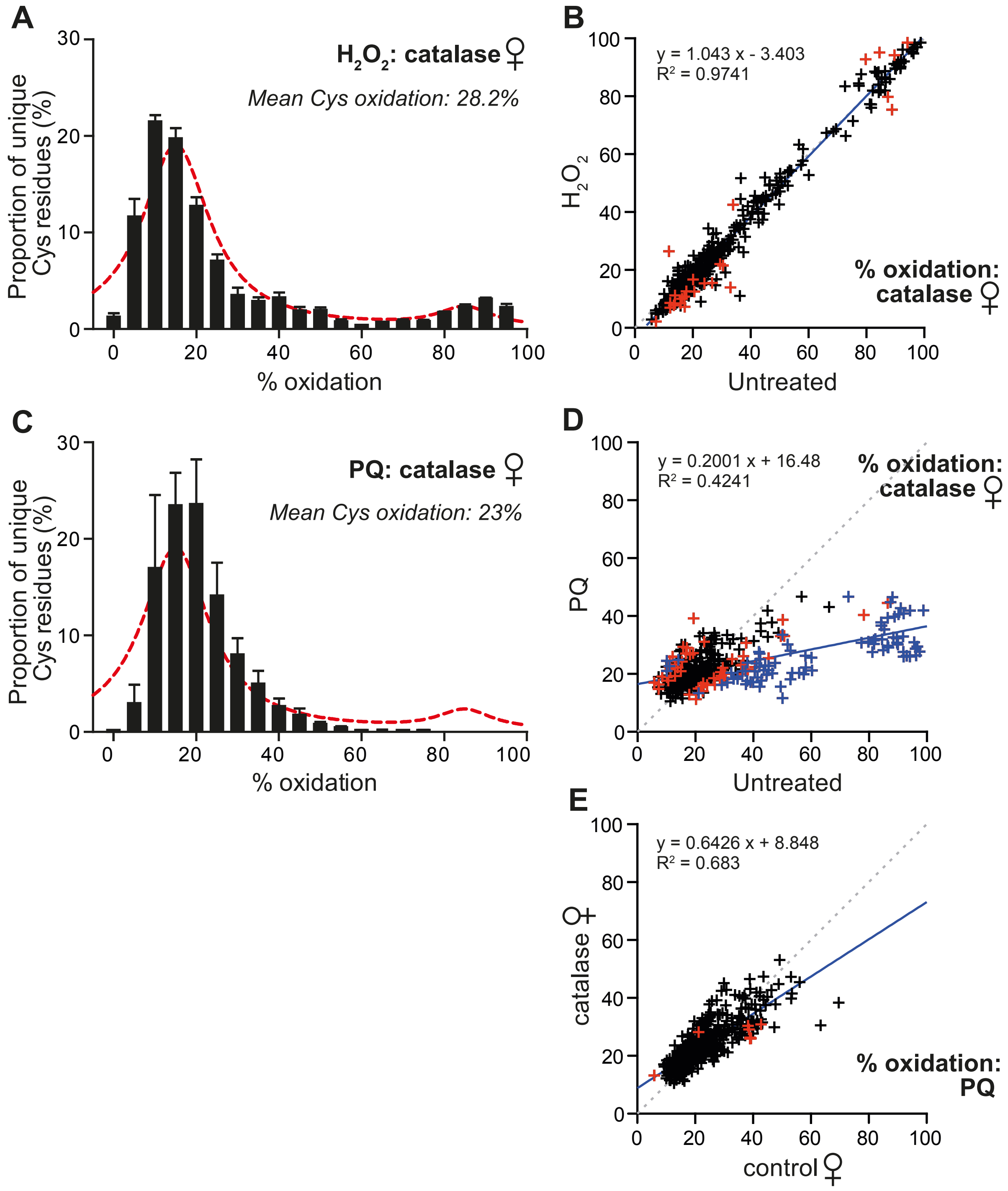

A
H_2O_2: catalase ♀
Mean Cys oxidation: 28.2%
Proportion of unique Cys residues (%)
% oxidation
B
y = 1.043 x - 3.403
R² = 0.9741
H_2O_2
% oxidation: catalase ♀
Untreated
C
PQ: catalase ♀
Mean Cys oxidation: 23%
Proportion of unique Cys residues (%)
% oxidation
D
y = 0.2001 x + 16.48
R² = 0.4241
% oxidation: catalase ♀
PQ
Untreated
E
y = 0.6426 x + 8.848
R² = 0.683
catalase ♀
% oxidation: PQ
control ♀

Figure S4. Effect of H_2O_2 and PQ Treatment on Cysteine Residue Redox State in Catalase Over-expressing Flies

(A) Distribution of total cysteine residue oxidation levels in catalase over-expressing flies exposed to dietary H_2O_2 (24 h treatment). Plotted is the proportion of the total number of peptides containing unique cysteine residues in each 5% quantile of % oxidation (mean ± SEM). The weighted arithmetic mean of the protein cysteine residue oxidation is indicated. The dashed red line is the manually fitted distribution using a Lorentizian equation for the untreated catalase over-expressing cohort.

(B) Oxidation state of cysteine residues present in catalase over-expressing flies exposed to H_2O_2 plotted against untreated flies. The dotted line has a slope of 1, while the continuous line is the least squares best-fit line to the data, with the equation for the line and Pearson's correlation coefficient indicated. The red symbols (n = 26) indicate cysteine residues with a p value smaller than 0.05 following a non-paired, two-tailed Student's t-test. No data points had a high stringency significance assessed by a Benjamini-Hochberg test. Total unique peptides = 485.

(C) Distribution of total protein thiol oxidation levels in young (7 d) catalase over-expressing flies exposed to PQ. Plotted is the proportion of the total number of peptides containing unique cysteine residues in each 5% quantile of % oxidation (mean ± SEM). The weighted arithmetic mean of the protein thiol oxidation is indicated. The dashed red line is the manually fitted distribution using a Lorentizian equation for the untreated catalase over-expressing cohort.

(D) Oxidation state of cysteine residues present in catalase over-expressing flies exposed to PQ plotted against untreated flies. The dotted line has a slope of 1, while the continuous line is the least squares best-fit line to the data, with the equation for the line and Pearson's correlation coefficient indicated. The red symbols (n = 139) indicate cysteine residues found to be significantly different using the less stringent non-paired two-tailed Student's t-test with a p value < 0.05. Blue symbols (n = 85) indicate cysteine residues found to be significantly different using the more stringent Benjamini-Hochberg-test. Total unique peptides = 429.

(E) Oxidation state of cysteine residues upon PQ treatment of catalase over-expressing flies plotted against control flies. The dotted line has a slope of 1, while the continuous line is the least squares best-fit line to the data, with the equation for the line and Pearson's correlation coefficient indicated. The red symbols (n = 8) indicate cysteine residues with a p value smaller than 0.05 following a non-paired, two-tailed Student's t-test. No data points had a high stringency significance assessed by a Benjamini-Hochberg test. Total unique peptides = 534.

SFig 4 is linked to Fig 4.

Figure S5

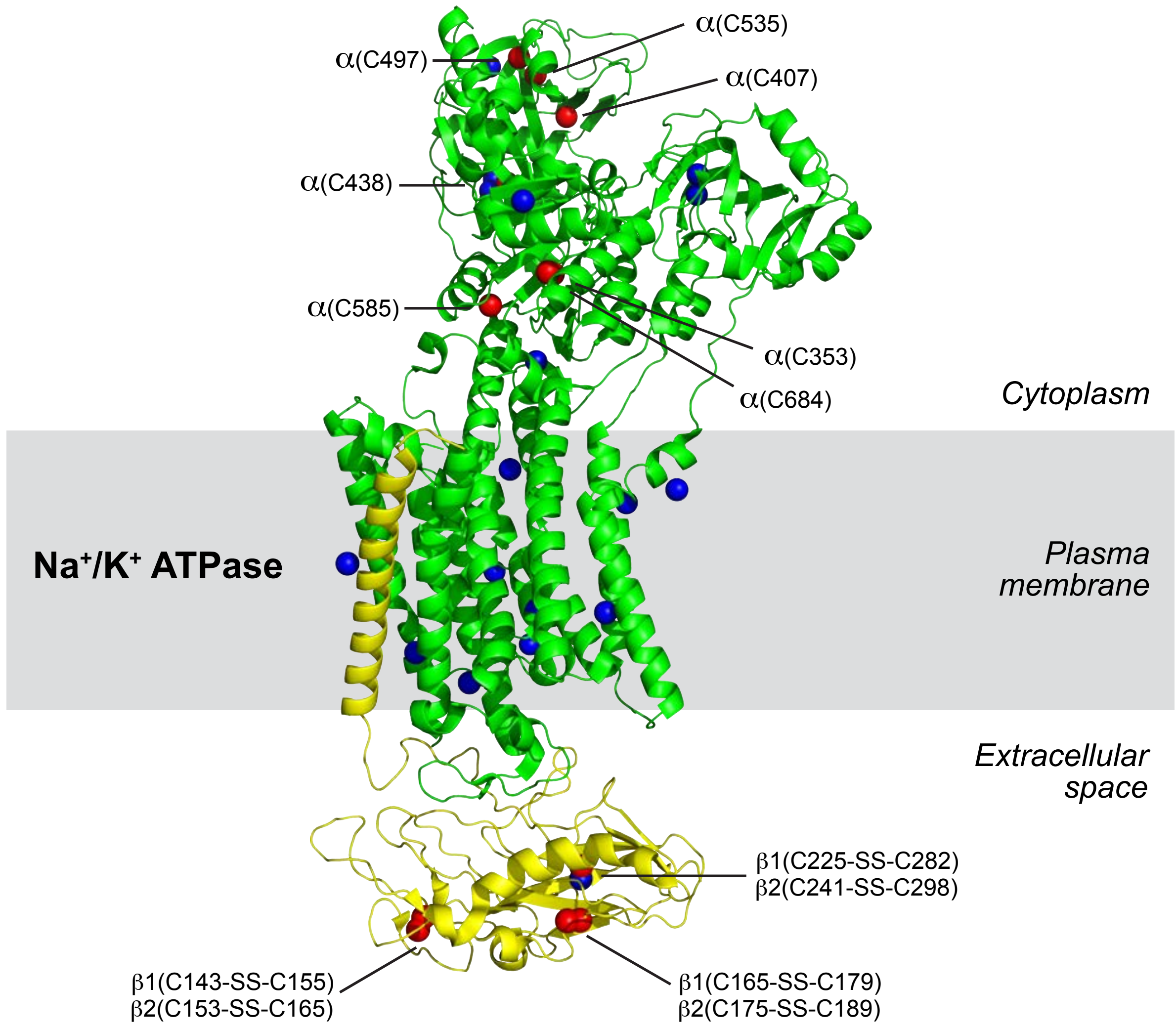

Subunit (Uniprot)	Cys residue	Control (% oxidation) Untreated	Control H_2O_2	Control PQ	Control Fasting	Catalase (% oxidation) Untreated	Catalase H_2O_2	Catalase PQ	Catalase Fasting
α(E1JIR4)	240	4.1 (5)	4.6 (5)	15.3 (5)	28.7 (5)	8.5 (5)	6.3 (5)	19.2 (5)	24.6 (5)
α(E1JIR4)	353	8.6 (5)	10.6 (5)	17.3 (5)	26.2 (5)	14.4 (5)	13.8 (5)	18.4 (5)	22.9 (5)
α(E1JIR4)	407	6.2 (5)	7.0 (5)	10.9 (5)	27.2 (5)	18.6 (5)	11.0 (5)	17.3 (5)	16.1 (5)
α(E1JIR4)	438	14.4 (5)	15.3 (5)	15.3 (5)	28.1 (4)	14.9 (5)	20.8 (5)	17.2 (4)	25.8 (5)
α(E1JIR4)	497	5.0 (5)	4.6 (4)	13.0 (5)	33.4 (5)	7.3 (5)	5.0 (3)	14.8 (5)	23.3 (5)
α(E1JIR4)	535	14.2 (5)	15.7 (5)	19.2 (4)	35.9 (5)	14.7 (5)	15.1 (5)	18.5 (2)	21.7 (4)
α(E1JIR4)	585	8.7 (1)	8.5 (3)					22.4 (2)	33.9 (1)
α(E1JIR4)	684	4.6 (5)	5.9 (5)	13.5 (5)	25.3 (5)	12.2 (5)	7.0 (5)	15.6 (5)	21.2 (5)
β1(Q24046)	143,155,165		81.3 (3)		30.4 (1)	77.1 (2)	69.7 (4)	18.0 (2)	15.8 (1)
β1(Q24046)	179			32.8 (5)	39.0 (5)		79.1 (1)	29.9 (1)	24.5 (2)
β1(Q24046)	282		88.0 (2)	39.9 (3)	75.8 (1)	87.0 (2)	80.3 (3)		
β2(Q24048)	8	20.2 (5)	18.2 (5)	32.4 (2)	29.8 (3)	23.9 (5)	15.1 (5)	27.7 (4)	33.6 (5)
β2(Q24048)	153			36.1 (5)	42.6 (4)		83.2 (1)	26.8 (2)	27.7 (2)
β2(Q24048)	153,165	81.1 (1)	78.7 (4)	48.9 (3)	55.2 (2)	87.4 (3)	79.8 (2)	44.8 (4)	41.3 (4)
β2(Q24048)	165	82.5 (1)	82.2 (1)	33.0 (5)	46.4 (5)	77.3 (3)	84.5 (4)	30.8 (4)	23.4 (3)
β2(Q24048)	175	83.6 (2)	76.1 (3)	32.8 (4)	30.0 (1)			29.0 (4)	30.9 (3)
β2(Q24048)	189	71.3 (1)	70.1 (5)		41.7 (3)	69.6 (4)	68.7 (5)	26.5 (2)	
β2(Q24048)	298	83.5 (1)	83.4 (3)	38.6 (1)	56.8 (3)	86.3 (2)	72.6 (2)	47.0 (2)	37.9 (4)
β3(Q7JS69)	34	23.3 (2)	35.8 (2)	19.4 (5)	29.9 (5)	26.3 (2)		20.6 (3)	17.6 (4)

Figure S5. The Redox Changes Observed During Stress Occur Across the Plasma Membrane

Plasma membrane Na^+/K^+ ATPase. *D. melanogaster* Na^+/K^+ ATPase contains two subunits, an α and a β subunit, with the β subunit having three isoforms, β1, β2 and β3. The monomeric structure of Na^+/K^+ ATPase from *S. acanthias* (2ZXE) contains two subunits: subunit α (green) is 77% homologous to the α subunit of *D. melanogaster* and subunit β1 (yellow) is 31%, 26% and 30% homologous to the β1, β2 and β3 subunits, respectively, of *D. melanogaster*. The sulphur atom of cysteine residues on the *S. acanthias* structure that are observed by OxICAT are shown as red spheres and numbered according to the *D. melanogaster* sequence. The sulphur atom of cysteines that are not detected by OxICAT are blue spheres. If they are present as a disulphide, their cysteine partner is also labelled. The table shows the oxidation state of each cysteine under different conditions with the number of times it was observed in parentheses.

SFig 5 is linked to Fig 5.

Figure S6

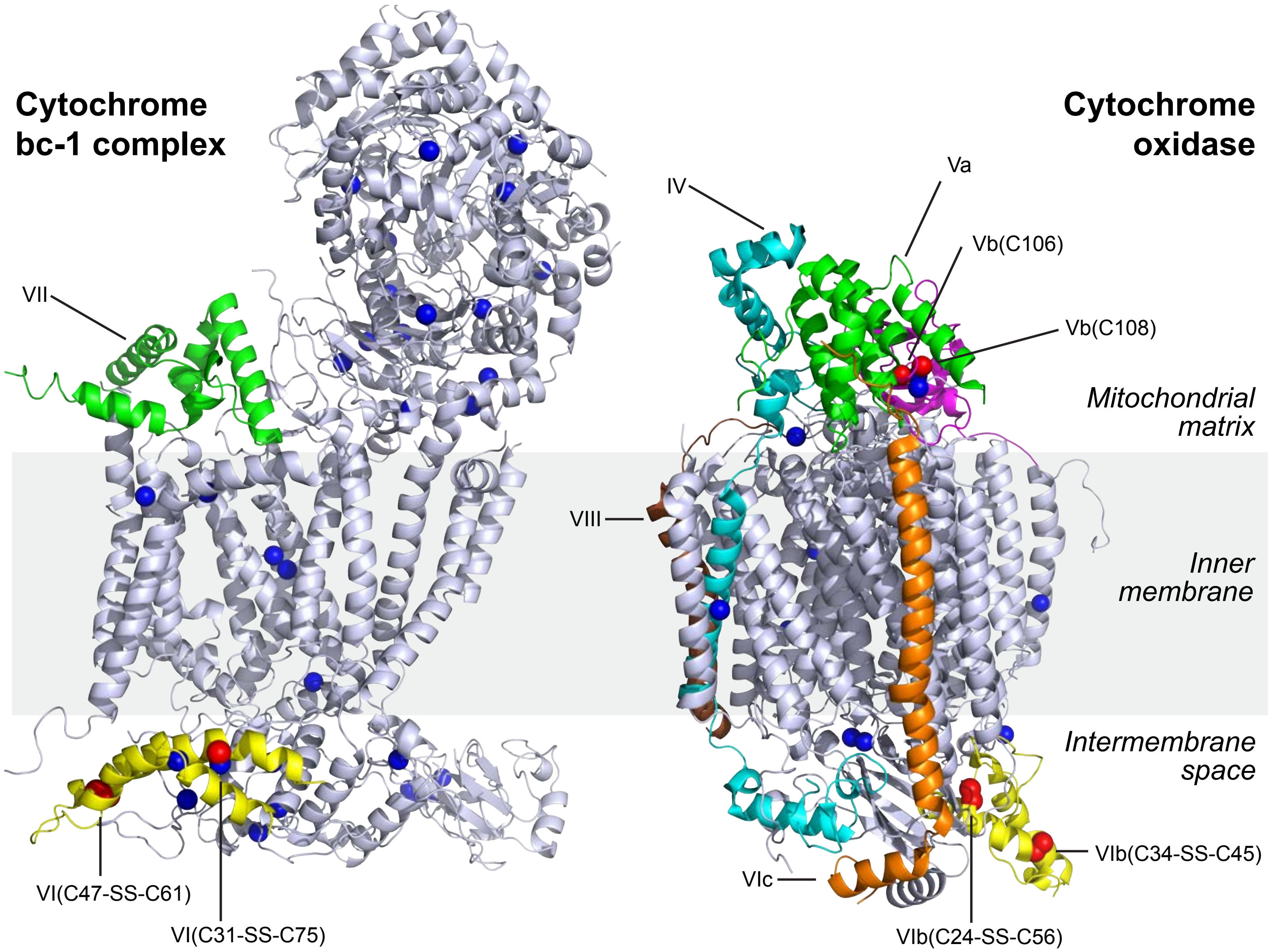

	Subunit (Uniprot)	Cys residue	Control (% oxidation)				Catalase (% oxidation)			
			Untreated	H_2O_2	PQ	Fasting	Untreated	H_2O_2	PQ	Fasting
**Cytochrome bc-1 complex**	VI (A8Y535)	47	94.7 (4)	94.2 (5)	37.1 (5)	52.1 (5)	91.9 (5)	92.7 (5)	40.9 (5)	50.0 (5)
	VI (A8Y535)	61 75		95.4 (2)			92.5 (3)	96.1 (4)		
	VII (Q9VXI6)	36	7.2 (5)	7.8 (5)	21.3 (5)	37.6 (5)	10.9 (5)	9.7 (4)	22.5 (4)	28.2 (4)
**Cytochrome oxidase**	IV (Q9VIQ8)	51	5.7 (5)	6.7 (5)	17.3 (5)	30.3 (5)	13.0 (5)	15.6 (5)	23.2 (5)	19.9 (5)
	IV (Q9VIQ8)	100			38.1 (4)	29.7 (3)			57.5 (2)	37.0 (4)
	Va (Q94514)	100	15.7 (3)	18.6 (4)	22.5 (5)	43.3 (5)	21.5 (5)	18.4 (5)	33.0 (5)	32.4 (4)
	Va (Q94514)	100 104	18.4 (5)	14.7 (5)	27.5 (4)		19.3 (5)	17.6 (5)	39.2 (3)	41.3 (4)
	Vb (Q9VMB9)	106 108	26.0 (3)	29.4 (4)			46.2 (1)			
	VIb (Q8IQW2)	45	87.5 (4)	89.6 (5)	42.3 (5)	52.1 (5)	90.2 (5)	89.5 (5)	40.7 (5)	47.7 (5)
	VIb (Q8IQW2)	24 34	93.4 (2)	86.1 (4)			72.6 (2)	79.9 (1)	50.0 (1)	78.6 (1)
	VIb (Q8IQW2)	24	98.3 (4)	97.4 (5)	42.5 (5)	53.9 (5)	98.8 (5)	98.6 (5)	42.0 (5)	47.4 (5)
	VIb (Q8IQW2)	56			43.5 (4)				42.7 (1)	44.1 (3)
	Vic (Q9VMS1)	77	6.6 (5)	4.9 (5)			6.6 (3)	4.4 (2)	28.9 (2)	59.3 (1)
	VIII (Q9VP19)	47		23.1 (4)			26.7 (3)	22.1 (1)		

Figure S6. The Redox Changes Observed During Stress Occur Within Mitochondria

Bos taurus cytochrome bc1 complex (1BE3) spans the mitochondrial inner membrane and contains 11 subunits. In the OxICAT experiments *D. melanogaster* cysteine-containing peptides were observed from subunits VI (yellow) and VII (green) which are 38% and 56% homologous to the bovine sequences. The structure of *B. taurus* cytochrome oxidase (3ASO) also spans the mitochondrial inner membrane and contains 13 subunits with cysteine-containing peptides observed from subunits IV (cyan), Va (green), Vb (magenta), VIb (yellow), VIc (orange) and VIII (brown) which are 34%, 53%, 44%, 56%, 37% and 23% homologous to their respective subunits in *D. melanogaster*. The sulphur atom of cysteine residues on the *B. taurus* structure that are observed by OxICAT are shown as red spheres and numbered according to the *D. melanogaster* sequence. The sulphur atom of cysteines that are not detected by OxICAT are blue spheres. If they are present as a disulphide, their cysteine partner is also labelled. The table shows the oxidation state of each cysteine under different conditions with the number of times it was observed in parentheses.

SFig 6 is linked to Fig 5.

Figure S7

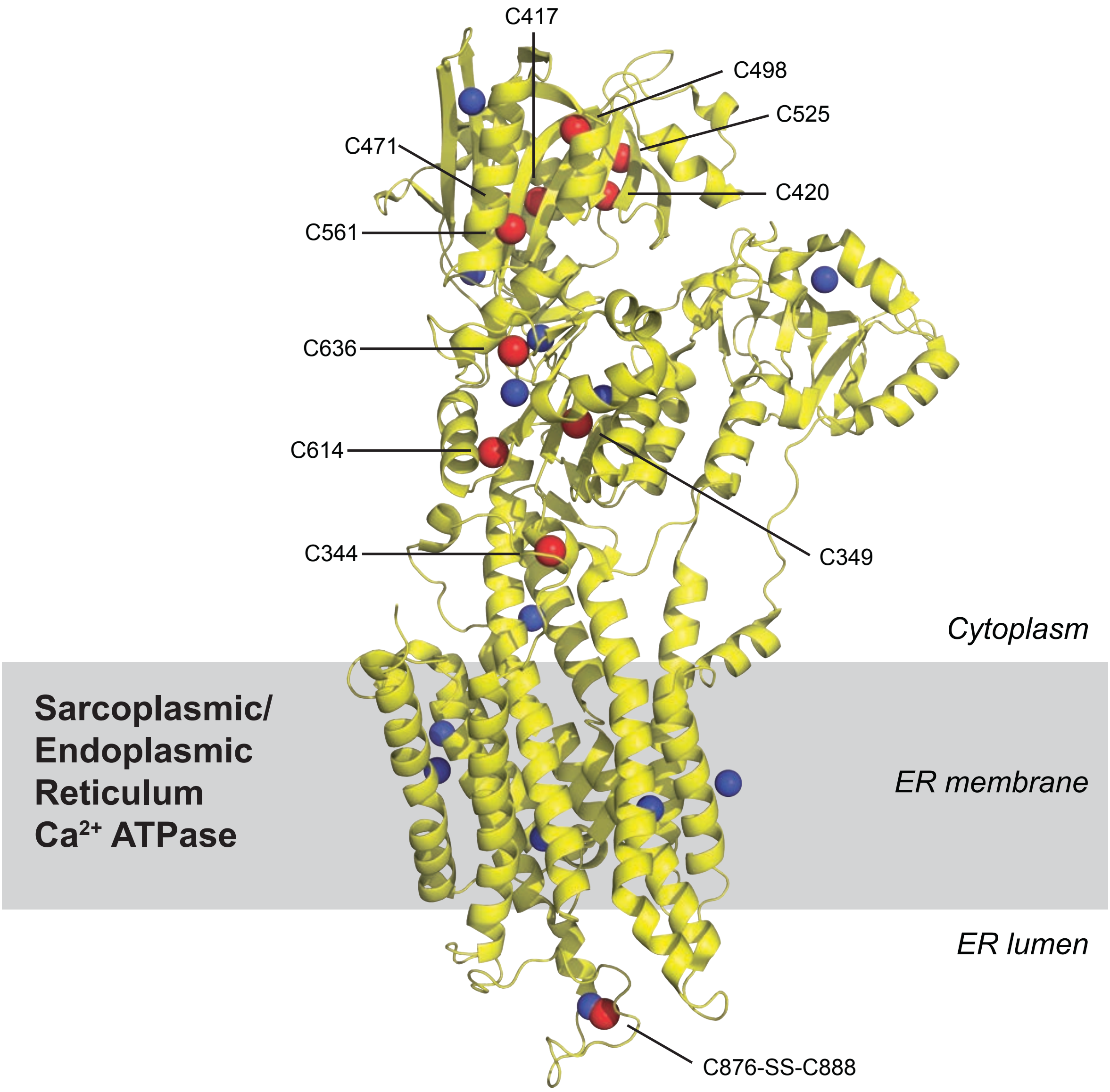

Subunit (Uniprot)	Cys residue	Control (% oxidation)				Catalase (% oxidation)			
		Untreated	H_2O_2	PQ	Fasting	Untreated	H_2O_2	PQ	Fasting
(P22700)	344 349	27.8 (4)	19.7 (5)	23.0 (5)	41.0 (5)	23.0 (5)	23.9 (5)	31.1 (5)	32.2 (5)
(P22700)	417 420	30.8 (4)	32.5 (4)	24.4 (5)	38.2 (4)	35.9 (5)	34.6 (5)	32.3 (4)	31.4 (5)
(P22700)	471	6.1 (5)	6.9 (5)	14.4 (5)	26.7 (5)	10.6 (5)	9.1 (5)	17.0 (5)	22.8 (5)
(P22700)	498	18.2 (5)	13.7 (5)	13.7 (5)	27.0 (5)	17.7 (5)	13.8 (5)	17.5 (5)	23.9 (5)
(P22700)	525	6.3 (4)	8.5 (4)			12.1 (3)	10.3 (3)	20.4 (1)	18.1 (1)
(P22700)	561	17.8 (2)	20.5 (4)	17.0 (5)	31.8 (5)	20.2 (4)	16.7 (4)	21.7 (5)	23.3 (4)
(P22700)	614	2.7 (1)						11.3 (1)	
(P22700)	636	5.3 (5)	7.5 (5)	9.4 (5)	22.6 (5)	9.4 (4)	6.3 (5)	16.3 (5)	15.1 (5)
(P22700)	876	68.2 (1)	62.2 (1)	26.5 (4)	44.7 (5)	71.3 (2)	68.3 (1)	27.8 (4)	30.2 (4)

Figure S7. The Redox Changes Observed During Stress Occur Within the ER

Oryctolagus cuniculus sarcoplasmic/endoplasmic reticulum Ca^{2+} ATPase (SERCA) spans the ER membrane and contains a single subunit (3W5C, yellow) and is 71% homologous to *D. melanogaster*. The sulphur atom of cysteine residues on the *O. cuniculus* structure that are observed by OxICAT are shown as red spheres and numbered according to the *D. melanogaster* sequence. The sulphur atom of cysteines that are not detected by OxICAT are blue spheres. If they are present as a disulphide, their cysteine partner is also labelled. The table shows the oxidation state of each cysteine under different conditions with the number of times it was observed in parentheses.

SFig 7 is linked to Fig 5.

Figure S8

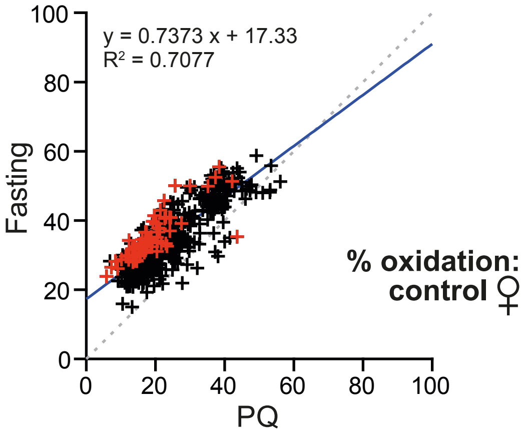

Figure S8. Comparison Between Effect of Fasting and PQ on Cysteine Residue Oxidation in Young (d 7) Control *D. melanogaster*

Oxidation state of cysteine residues present in control flies upon fasting plotted against PQ-treated flies. The dotted line has a slope of 1, while the continuous line is the least squares best-fit line to the data, with the equation for the line and Pearson's correlation coefficient indicated. The red symbols (n = 69) indicate cysteine residues with a p value smaller than 0.05 following a non-paired, two-tailed Student's t-test. No data points had a high stringency significance assessed by a Benjamini-Hochberg test. Total unique peptides = 616.

SFig 8 is linked to Fig 5.
